# General Approach to Enantiopure 1-Aminopyrrolizidines: Application to the Asymmetric Synthesis of the Loline Alkaloids

**DOI:** 10.1021/acs.joc.3c00047

**Published:** 2023-06-13

**Authors:** Stephen G. Davies, Ai M. Fletcher, Sean M. Linsdall, Paul M. Roberts, James E. Thomson

**Affiliations:** Department of Chemistry, Chemistry Research Laboratory, University of Oxford, Mansfield Road, Oxford OX1 3TA, U.K.

## Abstract

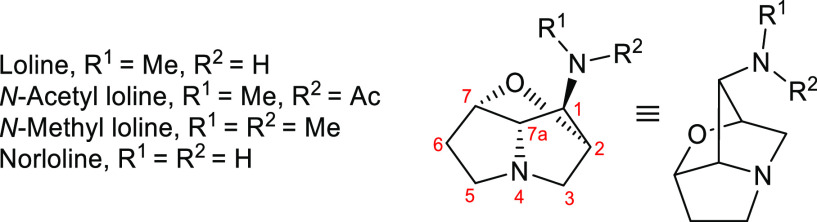

The synthesis of a range of loline alkaloids is reported. The C(7) and C(7a) stereogenic centers for the targets were formed by the established conjugate addition of lithium (*S*)-*N*-benzyl-*N*-(α-methylbenzyl)amide to *tert*-butyl 5-benzyloxypent-2-enoate, ensuing enolate oxidation to give an α-hydroxy-β-amino ester, and then formal exchange of the resultant amino and hydroxyl functionalities (via the intermediacy of the corresponding aziridinium ion) to give an α-amino-β-hydroxy ester. Subsequent transformation gave a 3-hydroxyprolinal derivative which was converted to the corresponding *N*-*tert*-butylsulfinylimine. Mannich-type reaction with the enolate derived from *O*-Boc protected methyl glycolate then formed the remaining C(1) and C(2) stereogenic centers for the targets. The 2,7-ether bridge was formed by a displacement reaction, completing construction of the loline alkaloid core. Facile manipulations then gave a range of loline alkaloids, including loline itself.

## Introduction

**Figure 1:**
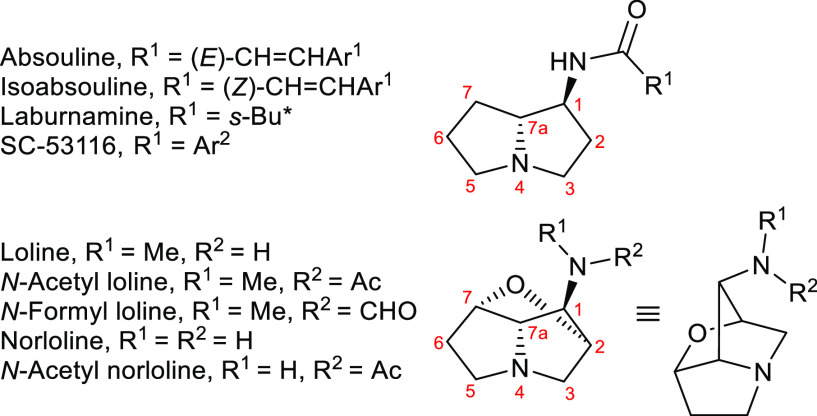
Representative 1-aminopyrrolizidine alkaloids and synthetic analogues. Ar^1^ = 4-methoxyphenyl. Ar^2^ = 2-methoxy-4-amino-5-chlorophenyl. *The question of configuration at the stereogenic center within the *s*-Bu side chain of laburnamine remains unaddressed.

1-Aminopyrrolizidine alkaloids—e.g., absoluline^1^ (Figure 1)—are a small but important family, all of which display interesting biological activity. Meanwhile, the synthetic analogue SC-53116^2^ (Figure 1) has been established as a potent and selective antagonist for 5-HT_4_ serotonin receptors. The 1-aminopyrrolizidine core is also present within the structures of the insecticidal loline alkaloids^3^—e.g., loline^4^ (Figure 1), the titular compound—which possess additional molecular complexity due to the presence of an ether bridge between C(2) and C(7) of the pyrrolizidine ring.

**Figure 2:**
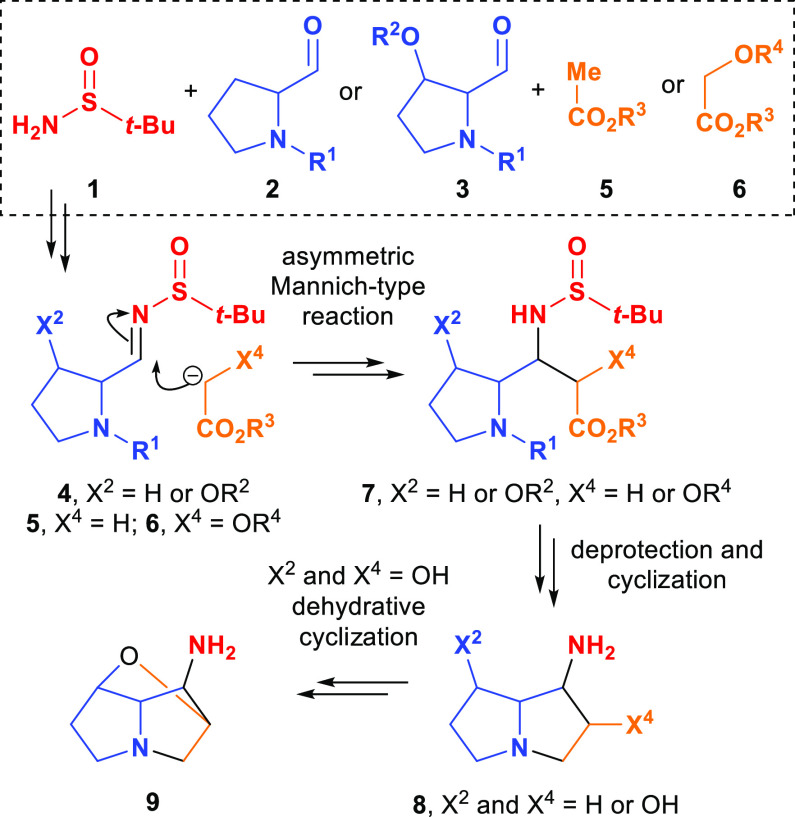
Proposed key sequence in the synthesis of 1-aminopyrrolizidine alkaloids (absouline-type or loline-type, and non-natural hydroxylated analogues).

No doubt as a result of their interesting structures and associated biological activities, there has been interest in the development of syntheses of compounds containing the 1-aminopyrrolizidine moiety, with absouline in particular proving a relatively popular target.^5^ Investigations directed toward the synthesis of the loline alkaloids are in contrast relatively limited,^6^ especially considering that they have been known for well over a century. The apparently simple structures of these alkaloids belie the relative challenge in their synthesis, which has often necessitated a high step count (particularly when the targets are required in an enantiomerically pure form) and in some cases has precluded synthetic endeavors.^7^ We envisaged the development of a flexible route to enable access to any of the members of the 1-aminopyrrolizidine alkaloid family (absouline-type or loline-type) and their analogues, including non-natural polyhydroxylated derivatives, based upon applications of an asymmetric Mannich-type reaction of an *N*-*tert*-butylsulfinylimine **4** with the enolate of either an acetate ester **5**(8) or an *O*-protected glycolate ester **6**(9) (as required).^10^ The requisite *N*-*tert*-butylsulfinylimines **4** would be derived from the condensation of either enantiomer of *N*-*tert*-butylsulfinylamide **1** with a suitably protected prolinal derivative **2** or 3-hydroxyprolinal derivative **3**.^11^ The former, **2**, would be readily available from either enantiomer of proline itself, whilst the latter, **3**, would be available in either enantiomeric form via adaptation of our synthesis of the stereoisomers of 3-hydroxyproline.^12^ Condensation of either enantiomer of *N*-*tert*-butylsulfinylamide **1** with a single enantiomer of the chiral aldehydes **2** and **3** would lead to epimeric (at sulfur) *N*-*tert*-butylsulfinylimines **4**. Literature precedent suggests that the stereochemical outcome of the Mannich-type reaction of such epimeric *N*-*tert*-butylsulfinylimines can be predominantly dictated by the configuration of the stereogenic center at the sulfur atom in both epimers, and so the reaction of both was anticipated to give rise to synthetically useful levels of diastereoselectivity.^13,14^ Thus, a range of stereoisomeric forms of the adducts **7** should be available via this approach. It was then proposed that these adducts **7** could undergo a deprotection and cyclization sequence of reactions to give a range of 1-aminopyrrolizidines **8**, encompassing the core of the absouline-type alkaloids (X^2^ = X^4^ = H) and various non-natural hydroxylated analogues thereof (X^2^ and/or X^4^ = OH). In the presence of an appropriate stereochemical disposition of hydroxy substituents (X^2^ and X^4^ = OH), a formal dehydrative cyclization may provide **9**,^15^ representing the core of the loline alkaloids (Figure 2). Herein, we describe our initial studies in this area that culminate in the synthesis of several members of the loline alkaloid family, including loline itself.

## Results and Discussion

**Scheme 1:**
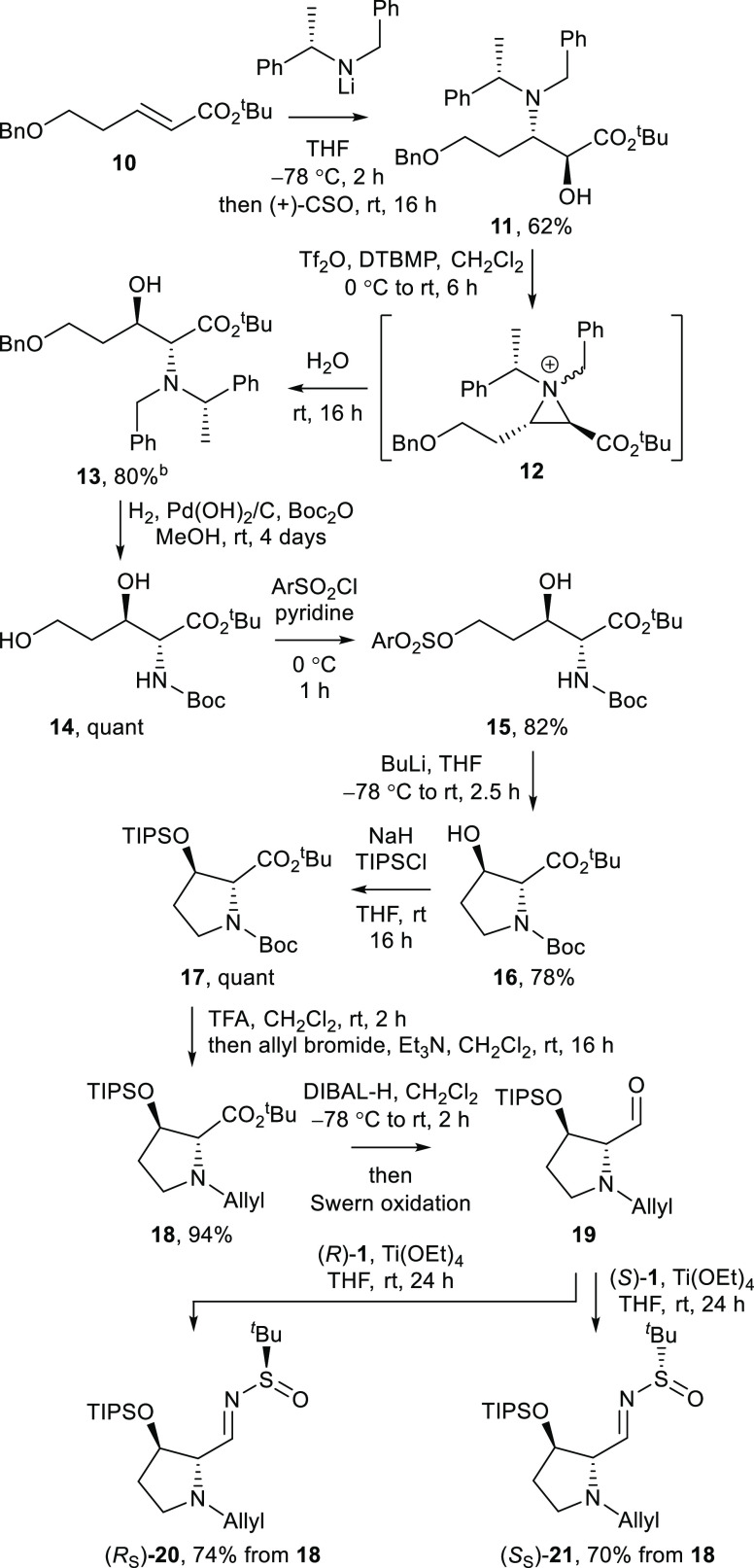
Preparation of *N*-*tert*-Butylsulfinylimines (*R_S_*)-**20** and (*S_S_*)-**21**^a^ DTBMP = 2,6-di-*tert*-butyl-4-methylphenol. Ar = 2-naphthyl. Combined yield after three iterations of the reaction.

In order to prepare the requisite *N*-*tert*-butylsulfinylimine required for the proposed asymmetric Mannich-type reaction toward the loline alkaloid core, the protected 3-hydroxyprolinate derivative **16** was first prepared in an analogous fashion to that previously described for the enantiomer,^12^ in five steps from α,β-unsaturated ester **10**. Briefly, the conjugate addition of lithium (*S*)-*N*-benzyl-*N*-(α-methylbenzyl)amide to **10** followed by in situ enolate oxidation with (+)-10-camphorsulfonyloxaziridine (CSO) gave α-hydroxy-β-amino ester **11** in 62% yield. Subsequent (formal) exchange of the amino and hydroxy functionalities via the intermediacy of the corresponding aziridinium ion **12** furnished α-amino-β-hydroxy ester **13** in 80% combined yield after three iterations of the reaction.^16^ Hydrogenolysis of **13** with in situ *N*-Boc protection gave **14** in quantitative yield, with **14** then undergoing chemoselective activation of the terminal hydroxy group upon treatment with 2-naphthalenesulfonyl chloride, giving **15** in 82% yield. Base-induced cyclization of **15** provided the 3-hydroxyprolinate derivative **16** in 78% yield. The identity, including relative configuration, of **16** was unambiguously established by single-crystal X-ray diffraction analysis (Figure S1, Supporting Information). The Flack *x* parameter^17^ for the crystal structure was −0.009(8), which is therefore also consistent with the assigned absolute (*R*,*R*)-configuration. A series of *N*- and *O*-protecting group manipulations of **16** was next performed, giving **18** (proceeding via **17**) in 94% yield over three steps. The *N*-allyl and *O*-triisopropylsilyl protecting group combination was chosen in order to avoid ensuing chemoselectivity issues: a series of selective *N*- and *O*-deprotections would subsequently be required to enable the requisite cyclization reactions in the construction of the loline alkaloid framework. Conversion of **18** to the requisite *N*-*tert*-butylsulfinylimine was planned by initial formation of the corresponding aldehyde **19** followed by condensation with the requisite enantiomer of *tert*-butylsulfinamide **1**. Previous reports concerning the asymmetric Mannich-type reaction of glycolate derivatives^9,14,18^ suggested that *N*-*tert*-butylsulfinylimine (*S_S_*)-**21** [derived from (*S*)-*tert*-butylsulfinamide **1**] would be required to give the correct C(1)–C(7a) relative configuration (pyrrolizidine numbering) required for the loline alkaloids. It was, however, of more broad interest to perform the condensation of both enantiomers of **1** with aldehyde **19** and thus, under standard conditions for this conversion, *tert*-butylsulfinimines (*R_S_*)-**20** and (*S_S_*)-**21** were formed as single diastereoisomers (epimeric at the sulfur atom) which were isolated in 74 and 70% yields (from **18**), respectively. The configuration of the newly formed double bond within (*R_S_*)-**20** and (*S_S_*)-**21** was assigned as (*E*) in both cases on the basis of the well-established outcome of this condensation process^10^ (Scheme 1).

**Scheme 2:**
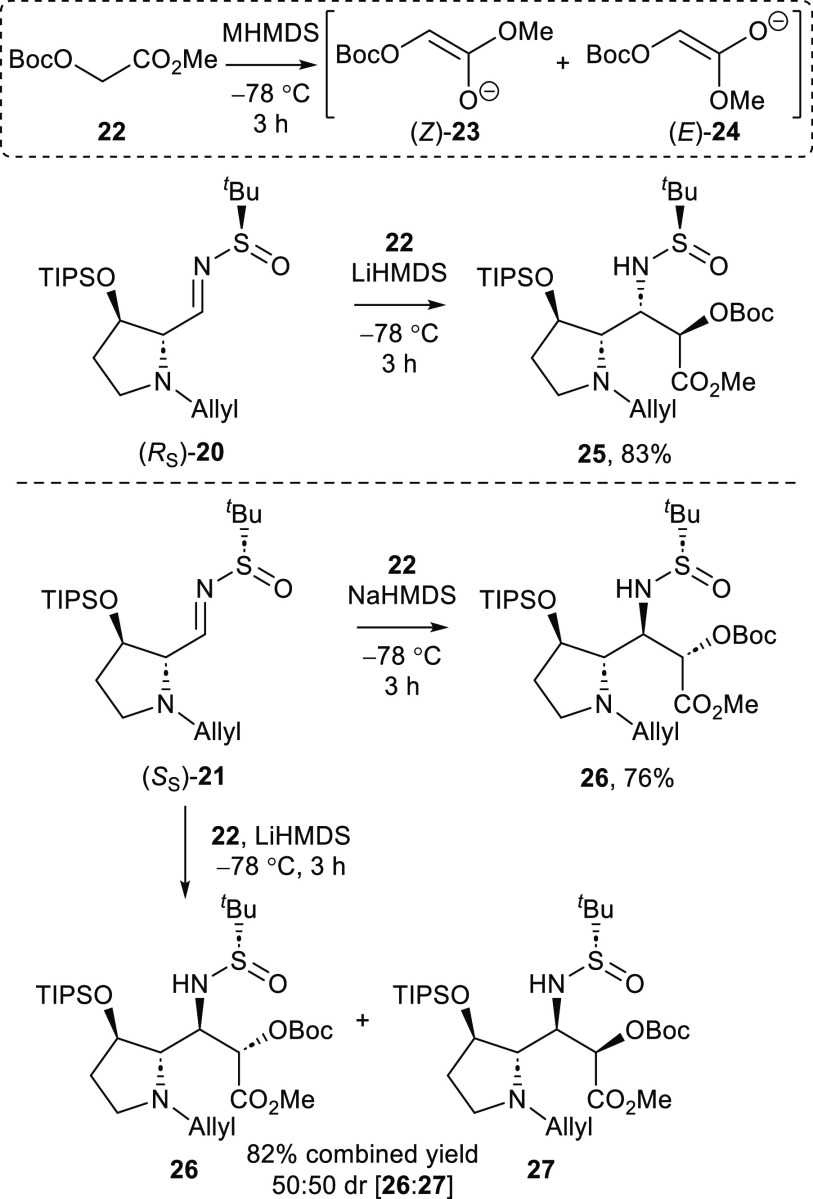
Asymmetric Mannich-type Reactions of *N*-*tert*-Butylsulfinylimines (*R_S_*)-**20** and (*S_S_*)-**21**

With the requisite *N*-*tert*-butylsulfinylimines (*R_S_*)-**20** and (*S_S_*)-**21** in hand, the key asymmetric Mannich-type reaction was investigated. In all of the previously reported application of this reaction,^9,14,18^ the optimal conditions have been found to involve deprotonation of an *O*-Boc protected glycolate ester (5 equiv) with LiHMDS or LDA (5 equiv), followed by addition to the requisite *N*-*tert*-butylsulfinylimine (1 equiv). Following this precedent, deprotonation of *O*-Boc methyl glycolate **22** with LiHMDS and subsequent reaction with *N*-*tert*-butylsulfinylimine (*R_S_*)-**20** delivered the adduct **25** as a single diastereoisomer (>95:5 dr) that was isolated in 83% yield (Scheme 2). In contrast, addition to (*S_S_*)-**21** under the same conditions gave a 50:50 mixture of diastereoisomeric adducts **26** and **27**, which proved inseparable upon chromatography and were thus isolated in 82% combined yield (Scheme 2). The absolute configurations within **25–27** were all unambiguously assigned by a combination of single-crystal X-ray diffraction analyses and ^1^H-^1^H NMR NOE analyses following their conversion to cyclized derivatives (see the Supporting Information for full details). In an effort to increase the diastereoselectivity of the asymmetric Mannich-type reaction, the use of NaHMDS (which has not been reported as a base to promote this reaction prior to this study) as an alternative base was considered. In the event, deprotonation of *O*-Boc methyl glycolate **22** with NaHMDS and addition to *N*-*tert*-butylsulfinylimine (*S_S_*)-**21** produced the adduct **26** as a single diastereoisomer (>95:5 dr) that was isolated in 76% yield (Scheme 2). The stereochemical outcomes of these asymmetric Mannich-type reactions [of *N*-*tert*-butylsulfinylimine (*R_S_*)-**20** with *O*-Boc methyl glycolate **22** promoted by LiHMDS and *N*-*tert*-butylsulfinylimine (*S_S_*)-**21** with *O*-Boc methyl glycolate **22** promoted by NaHMDS] are thus in complete accordance with the established diastereoselectivity of this reaction^9,14,18^ and indicate, in each case, that the reaction is under the dominant stereocontrol of the *N*-*tert*-butylsulfinyl substituent, also consistent with previous studies.^14^

The diastereoselectivity of this asymmetric Mannich-type reaction may be influenced by both the diastereoselectivity of the enolization of *O*-Boc methyl glycolate **22**(19) and the diastereofacialselectivity of addition of the intermediate (*Z*)-enolate **23**(20) and/or the (*E*)-enolate **24**(20) to the *N*-*tert*-butylsulfinylimines (*R_S_*)-**20** and (*S_S_*)-**21**. However, none of the previous reports concerning this reaction^9,14,18^ have provided insight into the diastereoselectivity of the requisite enolization process of *O*-Boc methyl glycolate, although several transition state models assuming addition of the corresponding (*E*)-enolate^20^ to the requisite *N*-*tert*-butylsulfinylimine(s) have been proposed. As *O*-silylation of the intermediate enolates has proven to be a valuable tool to interrogate the ratio of enolates formed under a range of conditions,^21^ this approach was used to investigate the diastereoselectivity of the enolization of *O*-Boc methyl glycolate **22** under the conditions employed herein. When *O*-Boc methyl glycolate **22** was subjected to deprotonation with LiHMDS followed by the addition of TMSCl,^19^ a 90:10 mixture of silylenolethers corresponding to trapping of the (*Z*)-enolate **23**(20) and (*E*)-enolate **24**,^20^ respectively, was observed. In contrast, enolization of *O*-Boc methyl glycolate **22** with NaHMDS followed by treatment with TMSCl under the same conditions^19^ implied that a 75:25 mixture of (*Z*)-enolate **23**(20) and (*E*)-enolate **24**,^20^ respectively, was present. Given these results, a simple mechanism involving irreversible enolization of *O*-Boc methyl glycolate **22** to give the (*E*)-enolate **24** followed by irreversible addition to the requisite *N*-*tert*-butylsulfinylimine is unable to account for the often very high (>95:5 dr) diastereoselectivities of this reaction class, as exemplified by the reaction of *O*-Boc methyl glycolate **22** with *N*-*tert*-butylsulfinylimine (*R_S_*)-**20** promoted by LiHMDS. On the contrary, a mechanism involving preferential kinetic (irreversible) formation of the (*Z*)-enolate **23**(19) followed by its addition to the requisite *N*-*tert*-butylsulfinylimine can rationalize the observed outcome, as can a mechanism involving interconversion of the (*Z*)-enolate **23**(19) and the (*E*)-enolate **24**(19) under the reaction conditions followed by the addition of either or both of the enolates to the requisite *N*-*tert*-butylsulfinylimine (Curtin–Hammett control); the latter may be of particular relevance in the case of enolization with NaHMDS.^21^ Further investigation is therefore required to ascertain the precise mechanistic origin of the observed diastereoselectivity in these reactions although, in any case, the observation that the use of NaHMDS offers improved overall diastereoselectivity is noteworthy and may prove a valuable insight for future applications of this reaction.

**Scheme 3:**
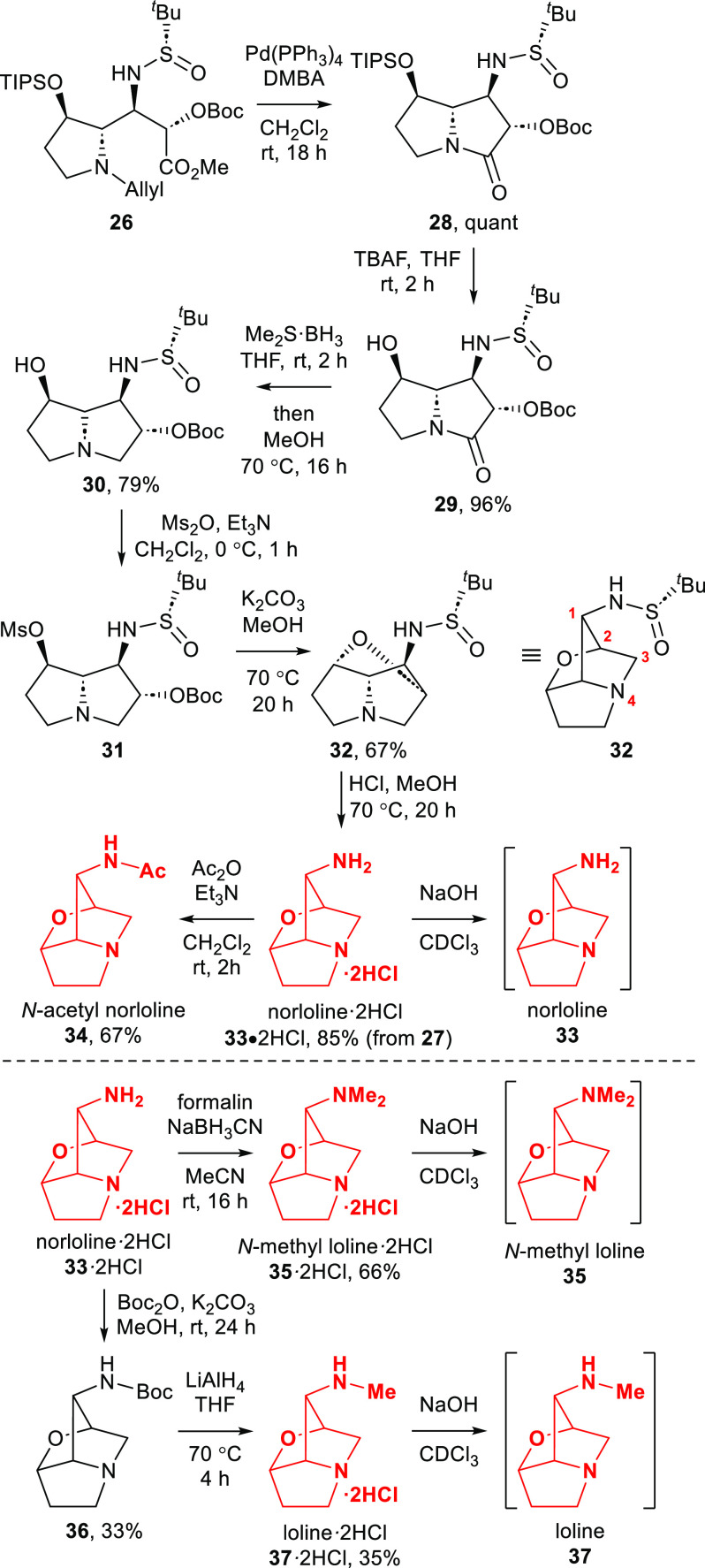
Preparation of Loline Alkaloids

With the required adduct **26** available as a single diastereoisomer, elaboration to the loline alkaloids was investigated (Scheme 3). Treatment of **26** with Pd(PPh_3_)_4_ and 1,3-dimethylbarbituric acid (DMBA)^22^ gave pyrrolizidinone **28** in quantitative yield. *O*-Desilylation of **28** using TBAF gave pyrrolizidinone **29** in 96% yield. The identity (including absolute configuration) of **29** was unambiguously confirmed by single-crystal X-ray diffraction analysis (Figure S2, Supporting Information). Subsequent reduction of **29** with Me_2_S·BH_3_ complex gave the borane adduct **30**·BH_3_ in 51% isolated yield after chromatography, which was unambiguously identified by single-crystal X-ray diffraction analysis (Figure S3, Supporting Information). This was quantitatively decomplexed by refluxing in MeOH to give the free pyrrolizidine **30**. However, **30** was obtained in 79% yield from **29** when the borane adduct **30**·BH_3_ was refluxed in MeOH without prior purification. The free pyrrolizidine **30** also proved amenable to analysis by single-crystal X-ray diffraction, providing further confirmation of both structure and absolute configuration (Figure S4, Supporting Information). The ether bridge was formed upon initial reaction of **30** with Ms_2_O and Et_3_N to give mesylate **31**, followed by immediate treatment with K_2_CO_3_ in MeOH at 70 °C, which resulted in the formation of *N*-*tert*-butylsulfinyl loline **32** in 67% yield. The presence of the ether bridge within **32** was evidenced by a strong correlation between C(2)*H* and *C*(7), and reciprocally between C(7)*H* and *C*(2) in its ^1^H-^13^C HMBC NMR spectrum. *N*-*tert*-Butylsulfinyl loline **32** decomposed to a complex mixture of unidentifiable products upon standing at rt. overnight, although its immediate treatment (without purification) with HCl in MeOH at 70 °C resulted in the removal of the *N*-*tert*-butylsulfinyl group to give norloline dihydrochloride **33**·2HCl in 85% yield from **30**. The identity (including absolute configuration) of norloline dihydrochloride **33**·2HCl was confirmed by single-crystal X-ray diffraction analysis (Figure S5, Supporting Information). Elaboration of **33**·2HCl to a range of the loline alkaloids was readily achieved using well-established procedures. Acetylation of **33**·2HCl gave *N*-acetyl loline **34** in 67% yield, whilst treatment of **33**·2HCl with 37% aqueous formaldehyde solution (formalin) and NaBH_3_CN in MeCN gave *N*-methyl loline **35**, which was isolated as the dihydrochloride **35**·2HCl in 66% yield. Mono-N-methylation of **33**·2HCl was achieved upon its initial treatment with Boc_2_O to give *N*-Boc norloline **36** in 33% yield, followed by subjecting to LiAlH_4_ to give loline **37**, which was isolated as the dihydrochloride **37**·2HCl in 35% yield. The alkaloid free bases norloline **33**, *N*-methyl loline **35**, and loline **37** were generated in situ in CDCl_3_ (in order to avoid previously reported handling issues)^23^ upon neutralization of the corresponding dihydrochloride salts with NaOH. The resultant solutions were then immediately subjected to NMR spectroscopic analysis. The ^1^H and ^13^C NMR data for norloline dihydrochloride **33**·2HCl, norline free base **33**, *N*-acetyl loline **34**, *N*-methyl loline dihydrochloride **35**·2HCl, *N*-methyl loline free base **35**, loline dihydrochloride **37**·2HCl, and loline free base **37** showed excellent agreement with those previously reported.^24^

## Conclusions

In conclusion, the synthesis of a range of loline alkaloids has been delineated. The pivotal reaction in the synthesis involves an asymmetric Mannich-type reaction of the enolate of *O*-Boc-protected methyl glycolate with an *N*-*tert*-butylsulfinylimine derived from the condensation of enantiopure *tert*-butylsulfinamide with a 3-hydroxyprolinal derivative. The synthesis should prove to be flexible for the preparation of a range of other compounds containing the 1-aminopyrrolizidine core, such as absouline-type alkaloids and non-natural hydroxylated analogues, simply by varying reaction partners in the asymmetric Mannich-type reaction.

## Data Availability

The data underlying this study are available in the published article and its Supporting Information.

## Supporting Information Available

Experimental details; characterization data; and copies of ^1^H and ^13^C NMR spectra (PDF)
