# Supplementary material for: General Approach to Enantiopure 1-Aminopyrrolizidines: Application to the Asymmetric Synthesis of the Loline Alkaloids
Source: J Org Chem. 2023 Jun 13;88(13):8093–8. doi: 10.1021/acs.joc.3c00047 (PMC10337030; doi:10.1021/acs.joc.3c00047)
Supplement: Supplementary file 1 — jo3c00047_si_001.pdf [file jo3c00047_si_001.pdf]

Supporting Information for

General Approach to Enantiopure 1-Aminopyrrolizidines: Application to the Asymmetric Synthesis of the Loline Alkaloids

Stephen G. Davies,* Ai M. Fletcher, Sean M. Linsdall,

Paul M. Roberts, and James E. Thomson

Department of Chemistry, Chemistry Research Laboratory,

University of Oxford, Mansfield Road, Oxford OX1 3TA, U.K.

steve.davies@chem.ox.ac.uk

Table of Contents **Page(s)**

1. Stereochemical Assignment of Adducts **25–27** **S2–S3**
2. Experimental **S4–S21**
3. X-ray Crystal Structures **S22–S30**
4. Copies of ^{1}H and ^{13}C NMR Spectra **S31–S55**
5. Data Comparison for the Loline Alkaloids **S56–S57**

1. Stereochemical Assignment of Adducts 25–27

In order to establish the configurations of the adducts **25**, **26** and **27** they were cyclised to the corresponding pyrrolizidinone derivatives in an effort to generate crystalline derivatives and/or enable examination of nOe correlations in the more conformationally rigid cyclic systems, and ultimately to facilitate elaboration to the loline alkaloids and their derivatives. Treatment of **25** [derived from *N*-*tert*-butylsulfinylimine (R_S)-**20**] with $Pd(PPh_3)_4$ and DMBA gave an ~70:30 mixture of *N*-deallylated pyrrolidine **38** and pyrrolizidinone **39**, the corresponding cyclized derivative, which were isolated in 45% and 17% yield, respectively, as single diastereoisomers in each case (Scheme S1). The relative configuration within **39** was established unambiguously by single crystal X-ray diffraction analysis (Figure S6). The absolute (1*S*,2*R*,7*R*,7a*S*,R_S)-configuration therefore followed from the known (*R*)-configuration of the sulfur atom. A Flack *x* parameter[15] of +0.007(16) for the crystal structure of **39** was consistent with this absolute configuration assignment. Thus, the absolute configurations within **25** and **38** could be assigned.

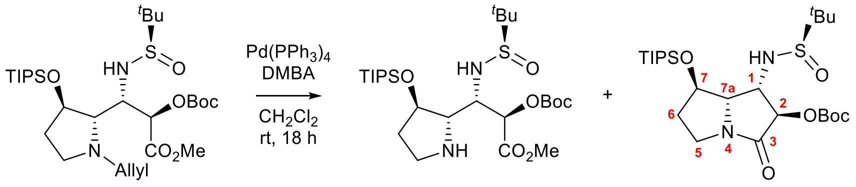

25 **38**, 45% **39**, 17%

Scheme S1.

Meanwhile, similar treatment of **26** [derived from *N*-*tert*-butylsulfinylimine (S_S)-**21**] with $Pd(PPh_3)_4$ and DMBA gave pyrrolizidinone **28** in quantitative yield. Subsequent *O*-desilylation of **28** using TBAF gave pyrrolizidinone **29** in 95% yield (Scheme S2). The identity of **29** was unambiguously confirmed by single crystal X-ray diffraction analysis (Figure S2). The absolute (1*R*,2*S*,7*R*,7a*S*,S_S)-configuration followed from the known (*S*)-configuration of the sulfur atom. A Flack *x* parameter[15] of –0.011(17) for the crystal structure of **29** was also consistent with this absolute configuration assignment. Thus, the absolute configurations within **26** and **28** could be assigned.

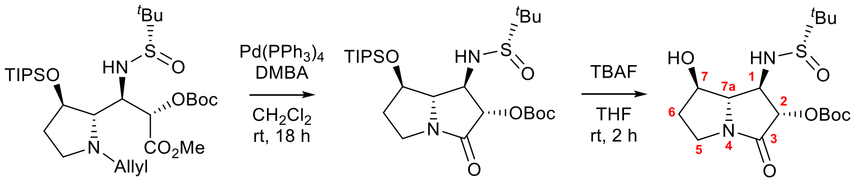

26 **28**, quant **29**, 95%

Scheme S2.

Following the same approach, the approximate 50:50 mixture of **26** and **27** [derived from *N*-*tert*-butylsulfinylimine (S_S)-**21**] was treated with $Pd(PPh_3)_4$ (polymer supported) and DMBA, which gave an approximate 50:50 mixture of the corresponding pyrrolizidinones **28** and **40**. Purification via chromatography

gave **28** and **40** in 35% and 46% yield, respectively. Treatment of pyrrolizidinone **40** with TBAF under the same conditions used successfully for chemoselective *O*-desilylation of **28** gave amino alcohol **41**—in which deprotection of both the *N-tert*-butylsulfinyl and *O*-TIPS groups had occurred—in 52% isolated yield (Scheme S3). The relative configurations at C(1) and C(7) of **41** were assigned on the basis of a NOESY analysis in C_6D_6, where a strong correlation could be seen between C(1)*H* and C(7)*H*, suggesting a *cis*-relationship between these two protons. It was not possible to establish the relative configurations at either C(2) or C(7a) from NOESY analysis due to overlapping proton resonances in the ^{1}H NMR spectrum. However, given that pyrrolizidinones **28**, **40** and **41** are all ultimately derived from *N-tert*-butylsulfinylimine (S_S)-**21**, they must share the same configurations at the common C(7) and C(7a) stereogenic centers. Given the results of the NOESY analysis applied to **41**, the relative configurations of C(1), C(7) and C(7a) in **28**, **40** and **41** must be the same and therefore **28** and **40** must be related as epimers at C(2). Thus, the absolute configurations of **27**, **40** and **41** were assigned accordingly.

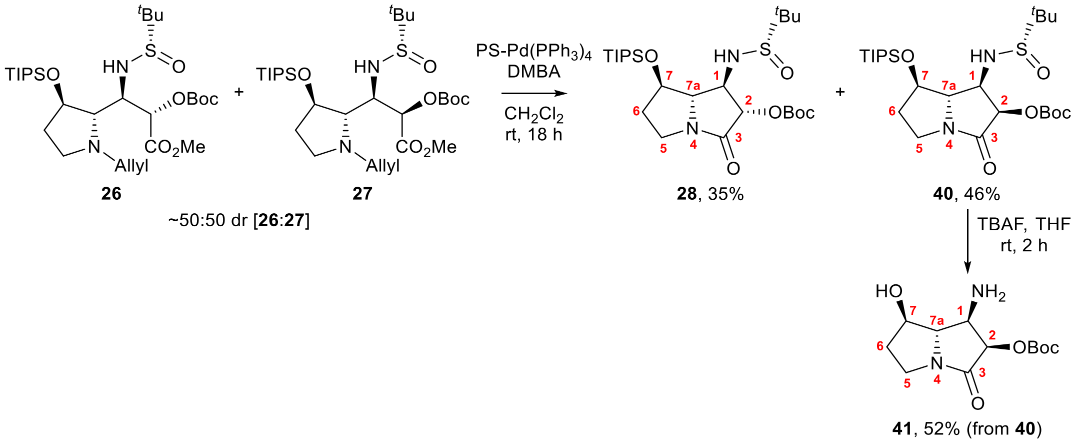

Scheme S3.

2. Experimental

General Experimental Details

Reactions involving moisture-sensitive reagents were carried out under a nitrogen atmosphere using standard vacuum line techniques and glassware that was flame-dried and cooled under nitrogen before use. Solvents were dried according to the procedure outlined by Grubbs and co-workers.[1] Water was purified by an Elix® UV-10 system. Organic layers were dried over $MgSO_4$ unless otherwise stated. Thin layer chromatography was performed on aluminium plates coated with 60 F_{254} silica. Flash column chromatography was performed on Kieselgel 60 silica on a glass column.

Melting points were recorded on a Gallenkamp Hot Stage apparatus. Optical rotations were recorded on a Perkin-Elmer 241 polarimeter with a water-jacketed 10 cm cell. Specific rotations are reported in 10^{-1} deg cm^2 g^{-1} and concentrations in g/100 mL. IR spectra were recorded on a Bruker Tensor 27 FT-IR spectrometer using an ATR module. Selected characteristic peaks are reported in cm^{-1}. NMR spectra were recorded on Bruker Avance spectrometers in the deuterated solvent stated. The field was locked by external referencing to the relevant deuteron resonance. Low-resolution mass spectra were recorded on either a VG MassLab 20-250 or a Micromass Platform 1 spectrometer. Accurate mass measurements were run on either a Bruker MicroTOF internally calibrated with polyalanine, or a Micromass GCT instrument fitted with a Scientific Glass Instruments BPX5 column (15 m × 0.25 mm) using amyl acetate as a lock mass.

All compounds were formed, isolated and used as single diastereoisomers (>95:5 dr), as judged by ^{1}H NMR spectroscopic analysis of the crude reaction mixture and purified product in all cases, unless otherwise indicated.

Experimental Data

***tert*-Butyl (2*S*,3*S*,α*S*)-2-hydroxy-3-[*N*-benzyl-*N*-(α-methylbenzyl)amino]-5-benzyloxypentanoate 11**

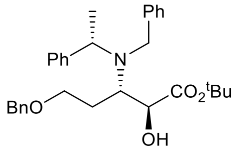

^{n}BuLi (2.5 M in hexanes, 118 mL, 296 mmol) was added dropwise to a stirred solution of (*S*)-*N*-benyl-*N*-(α-methylbenzyl)amine (64.4 g, 305 mmol, >99% ee) in THF (663 mL) at −78 °C, and the resultant mixture was stirred at −78 °C for 30 min. A solution of **10** (50.0 g, 191 mmol) in THF (532 mL) was added via cannula and the resultant mixture was stirred at −78 °C for 2 h. (+)-10-Camphorsulfonyloxaziridine (75.0 g, 325 mmol)

[1] Pangborn, A. B.; Giardello, M. A.; Grubbs, R. H.; Rosen, R. K.; Timmers, F. J. *Organometallics* **1996**, *15*, 1518.

was added and the reaction mixture was allowed to warm to rt and stirred at rt for 12 h. Satd aq NH_4Cl (250 mL) was added, and the resultant mixture was concentrated *in vacuo*. The residue was partitioned between CH_2Cl_2 (250 mL) and 10% aq citric acid (250 mL), the aqueous layer was extracted with CH_2Cl_2 (2 × 250 mL), and the combined organics were washed sequentially with satd aq $NaHCO_3$ (250 mL) and brine (250 mL), then dried, filtered and concentrated *in vacuo.* Purification via flash column chromatography (eluent 30–40 °C petrol/Et_2O, 10:1 increased to 30–40 °C petrol/Et_2O, 7.5:1) gave **11** as a colourless oil (57.4 g, 62%); $[\alpha]_D^{25}$ +30.6 (*c* 1.0 in $CHCl_3$); ν_{max} 3497, 1718; ^1H NMR ($CDCl_3$, 400 MHz) 7.45–7.20 (m, 15H); 4.50 (d, 1H, *J* 11.5), 4.44 (d, 1H, *J* 11.5), 4.29 (d, 1H, *J* 15.6), 3.93 (q, 1H *J* 7.1), 3.76–3.69 (m, 2H), 3.68 (d, 1H, *J* 15.6), 3.66–3.59 (m, 2H), 2.94 (d, 1H, *J* 5.8), 1.89–1.75 (m, 1H), 1.50–1.42 (m, 1H), 1.38 (s, 9H), 1.33 (d, 3H, d, *J* 7.1); $^{13}C\{^1H\}$ NMR ($CDCl_3$, 100 MHz) 174.5, 142.8, 142.4, 138.9, 128.4, 128.4, 128.4, 128.3, 128.2, 127.9, 127.6, 127.2, 126.6, 82.7, 73.0, 71.6, 67.6, 58.1, 55.4, 51.0, 28.1, 28.0, 19.5; HRMS (ESI^+) $C_{31}H_{40}NO_4^+$ ($[M+H]^+$) requires 490.2952; found 490.2951.

***tert*-Butyl (2*R*,3*R*,α*S*)-2-[*N*-benzyl-*N*-(α-methylbenzyl)]-3-hydroxy-5-benzyloxypentanoate 13**

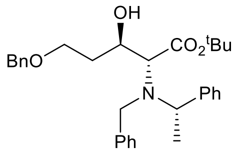

Run 1: Tf_2O (49.7 g, 176 mmol) was added to a stirred solution of **11** (57.4 g, 117 mmol) and DTBMP (72.0 g, 351 mmol) in CH_2Cl_2 (585 mL) at 0 °C, and the resultant mixture was stirred at 0 °C for 1 h then at rt for 5 h. H_2O (115 mL) was added and the resultant mixture was stirred at rt for 24 h. H_2O (1 L) was added and the reaction mixture was extracted with CH_2Cl_2 (3 × 500 mL). The combined organics were dried, filtered and concentrated *in vacuo*. The residue was suspended in Et_2O (750 mL), and the resultant suspension was filtered and concentrated *in vacuo*. Purification via flash column chromatography (eluent 30–40 °C petrol/Et_2O, 5:1) gave **11** as a colourless oil (32.2 g, 56%). Further elution gave **13** as a colourless oil (25.2 g, 44%); $[\alpha]_D^{25}$ +36.0 (*c* 1.0 in $CHCl_3$); ν_{max} 3503, 1724; ^1H NMR ($CDCl_3$, 400 MHz) 7.39–7.18 (m, 15H), 4.40 (d, 1H, *J* 11.9), 4.35 (d, 1H, *J* 11.9), 4.21 (d, 1H, *J* 14.3), 4.11–3.98 (m, 2H,), 3.89 (d, 1H, *J* 14.3), 3.43–3.29 (m, 2H), 3.21 (d, 1H, *J* 9.1), 3.07 (d, 1H, *J* 4.6), 2.01 (dddd, 1H, *J* 15.0, 7.2, 4.9, 2.3), 1.59 (s, 9H), 1.44 (d, 3H, *J* 6.9), 1.32 (dddd, 1H, *J* 15.0, 8.8, 6.7, 4.6); $^{13}C\{^1H\}$ NMR ($CDCl_3$, 100 MHz) 173.3, 143.5, 140.6, 138.2, 129.2, 128.5, 128.4, 128.3, 128.2, 127.7, 127.1, 127.0, 81.3, 73.2, 70.6, 68.7, 63.8, 56.9, 52.1, 33.0, 28.4, 13.4; HRMS (ESI^+) $C_{31}H_{40}NO_4^+$ ($[M+H]^+$) requires 490.2952; found 490.2949.

Run 2: Tf_2O (27.6 g, 98.1 mmol) was added to a stirred solution of **11** (32.0 g, 65.4 mmol) and DTBMP (40.3 g, 196 mmol) in CH_2Cl_2 (327 mL) at 0 °C, and the resultant mixture was stirred at 0 °C for 1 h then at rt for 5 h. H_2O (64.0 mL) was added and the resultant mixture was stirred at rt for 24 h. H_2O (500 mL) was added and the reaction mixture was extracted with CH_2Cl_2 (3 × 500 mL). The combined organics were dried, filtered and concentrated *in vacuo*. The residue was suspended in Et_2O (500 mL), and the resultant suspension was filtered and concentrated *in vacuo*. Purification via flash column chromatography (eluent 30–40 °C petrol/Et_2O, 5:1) gave **11** as a colourless oil (17.6 g, 55%).[2] Further elution gave **13** as a colourless oil (14.1 g, 44%).[2]

Run 3: Tf_2O (15.2 g, 53.9 mmol) was added to a stirred solution of **11** (17.6 g, 35.9 mmol) and DTBMP (22.1 g, 108 mmol) in CH_2Cl_2 (179 mL) at 0 °C, and the resultant mixture was stirred at 0 °C for 1 h then at rt for 5 h. H_2O (35.0 mL) was added and the resultant mixture was stirred at rt for 24 h. H_2O (250 mL) was added and the reaction mixture was extracted with CH_2Cl_2 (3 × 250 mL). The combined organics were dried, filtered and concentrated *in vacuo*. The residue was suspended in Et_2O (250 mL), and the resultant suspension was filtered and concentrated *in vacuo*. Purification via flash column chromatography (eluent 30–40 °C petrol/Et_2O, 5:1) gave **11** as a colourless oil (9.55g, 54%).[3] Further elution gave **13** as a colourless oil (6.80 g, 39%).[3]

***tert*-Butyl (2*R*,3*R*)-2-[(*N*-*tert*-butoxycarbonyl)amino]-3,5-dihydroxypentanoate 14**

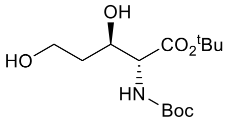

$Pd(OH)_2/C$ (8.80 g, 20% w/w) was added to a degassed solution of **13** (44.0 g, 90.0 mmol) and Boc_2O (39.0 g, 180 mmol) in MeOH (300 mL) and the resultant mixture was placed under an atmosphere of H_2 (5 atm) and stirred at rt for 4 days. The reaction mixture was filtered through Celite® (eluent MeOH) and concentrated *in vacuo*. Purification via flash column chromatography (eluent 30–40 °C petrol/Et_2O, 1:1 increased to Et_2O) gave **14** as a white solid (31.1 g, quant); $[\alpha]_D^{25}$ −30.0 (*c* 1.0 in MeOH); ν_{max} 3390, 1697; ^1H NMR (400 MHz, MeOH-d_4) 4.12 (d, 1H, *J* 5.3), 4.02 (ddd, 1H *J* 9.0, 5.2, 3.7), 3.80–3.70 (m, 2H), 1.86–1.71 (m, 2H), 1.53 (s, 9H), 1.50 (s, 9H); $^{13}C\{^1H\}$ NMR (100 MHz, MeOH-d_4) 171.5, 157.9, 82.9, 80.7, 70.3, 60.9, 59.8, 37.1, 28.7, 28.3; HRMS (ESI$^+$) $C_{14}H_{27}NNaO_6^+$ ($[M+Na]^+$) requires 328.1731; found 328.1730.

[2] After Run 2, the amount of recovered **11** (from Run 1, plus Run 2) was thus 31% and the overall yield of **13** (from Run 1, plus Run 2) was thus 68% based on the original amount of the starting material **11**.
[3] After Run 3, the amount of recovered **11** (from Run 1, plus Run 2, plus Run 3) was thus 17% and the overall yield of **13** (from Run 1, plus Run 2, plus Run 3) was thus 80% based on the original amount of the starting material **11**.

***tert*-Butyl (2*R*,3*R*)-2-(*N*-*tert*-butoxycarbonylamino)-3-hydroxy-5-(naphthalen-2′-ylsulfonyloxy)pentanoate 15**

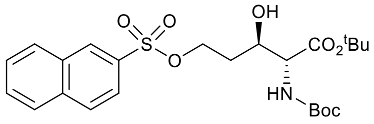

2-NapSO_2Cl (33.4 g, 147 mmol) was added to a stirred solution of **14** (30.0 g, 98.2 mmol) in pyridine (196 mL) at 0 °C, and the resultant mixture was stirred at 0 °C for 1 h. EtOAc (500 mL) was added and the resultant mixture was washed with satd aq $CuSO_4$ (3 × 400 mL). The combined aqueous extracts were extracted with EtOAc (2 × 400 mL) then the combined organics were washed sequentially with H_2O (5 × 400 mL), satd aq $NaHCO_3$ (3 × 400 mL) and brine (2 × 400 mL), then dried, filtered and concentrated *in vacuo*. Purification via flash column chromatography (eluent 30–40 °C petrol/Et_2O, 1:1) gave **15** as a colourless oil (37.0 g, 82%); $[\alpha]_D^{25}$ +12.8 (*c* 1.0 in MeOH); ν_{max} 3396, 1738, 1701; ^{1}H NMR ($CDCl_3$, 400 MHz) 8.48 (d, 1H, *J* 1.8), 8.00–7.82 (m, 4H), 7.70–7.58 (m, 2H), 5.44 (d, 1H, *J* 7.3), 4.32–4.19 (m, 3H), 4.08 (dt, 1H, *J* 9.3, 4.2), 3.57 (br s, 1H), 1.84–1.70 (m, 2H), 1.43 (s, 9H), 1.41 (s, 9H); $^{13}C\{^1H\}$ NMR ($CDCl_3$, 100 MHz) 169.0, 156.7, 135.4, 132.9, 132.1, 129.8, 129.8, 129.4, 128.1, 127.9, 122.6, 83.4, 80.7, 69.4, 67.7, 59.0, 32.3, 28.2, 27.9; HRMS (ESI$^+$) $C_{24}H_{34}NO_8S^+$ ([M+H]$^+$) requires 496.2000; found 496.1995.

(2*R*,3*R*)-1,2-Bis-*tert*-butoxycarbonyl-3-pyrrolidinol 16

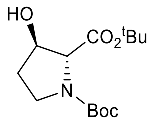

^{n}BuLi (2.5 M in hexanes, 32.4 mL, 81.0 mmol) was added dropwise to a stirred solution of **15** (36.5 g, 73.7 mmol) in THF (737 mL) at −78 °C, and the resultant mixture was stirred at −78 °C for 30 min then rt for 2 h. Satd aq NH_4Cl (200 mL) was added and the resultant mixture was extracted with CH_2Cl_2 (3 × 250 mL). The combined organics were washed with brine (250 mL), then dried, filtered and concentrated *in vacuo*. Purification via flash column chromatography (eluent 30–40 °C petrol/Et_2O, 1:2) gave **16** as a white solid (16.5 g, 78%); mp 100–102 °C; $[\alpha]_D^{25}$ +20.3 (*c* 1.0 in $CHCl_3$); ν_{max} 3431, 1739, 1702, 1678; ^{1}H NMR (PhMe-*d*$_8$, 363 K, 500 MHz) 4.18–4.10 (m, 2H), 3.52–3.42 (m, 2H), 1.97–1.81 (m, 2H), 1.64–1.50 (m, 1H), 1.42 (s, 9H), 1.35 (s, 9H); $^{13}C\{^1H\}$ NMR (PhMe-*d*$_8$, 363 K, 125 MHz) 170.5, 137.7, 81.0, 79.5, 75.6, 69.7, 45.0, 32.9, 28.7, 28.3; HRMS (ESI$^+$) $C_{14}H_{26}NO_5^+$ ([M+H]$^+$) requires 288.1805; found 288.1811.

(2*R*,3*R*)-1,2-Bis-*tert*-butoxycarbonyl-3-triisopropylsilyloxypyrrolidine 17

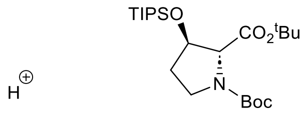

A solution of **16** (4.86 g, 16.9 mmol) in THF (40.0 mL) was added dropwise to a suspension of NaH (60% dispersion in mineral oil, 676 mg, 16.9 mmol) in THF (45.0 mL) at rt, and the resultant mixture was stirred at rt for 15 min. TIPSCl (7.20 mL, 33.8 mmol) was added and the resultant mixture was stirred at rt for 16 h. H_2O (100 mL) was added and the reaction mixture was extracted with CH_2Cl_2 (3 × 100 mL). The combined organics were washed with brine (100 mL), then dried, filtered and concentrated *in vacuo*. Purification via flash column chromatography (eluent 30–40 °C petrol/Et_2O, 20:1) gave **17** as a colourless oil (7.78 g, quant); $[\alpha]_D^{25}$ +20.3 (*c* 1.0 in $CHCl_3$); ν_{max} 1740, 1706; ^1H NMR (400 MHz, $CDCl_3$, rotameric) 4.46–4.42 (m, 1H), 4.17 (s, 0.35H), 4.04 (s, 0.65H), 3.68–3.47 (m, 2H), 2.04–1.92 (m, 1H), 1.87–1.81 (m, 1H), 1.48–1.43 (m, 18H), 1.09–1.04 (m, 21H); $^{13}C\{^1H\}$ NMR (100 MHz, $CDCl_3$, rotameric) 12.1, 17.9, 17.9, 28.0, 28.2, 28.4, 28.4, 33.0, 33.8, 44.4, 44.8, 69.3, 69.6, 75.1, 76.1, 79.6, 81.2, 135.6, 170.2; HRMS (ESI^+) $C_{23}H_{46}NO_5Si^+$ ($[M+H]^+$) requires 444.3140; found 444.3142.

(2*R*,3*R*)-1-Allyl-2-*tert*-butoxycarbonyl-3-triisopropylsilyloxypyrrolidine 18

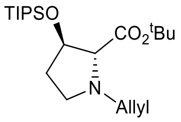

Step 1. TFA (4.81 mL, 62.8 mmol) was added dropwise to a stirred solution of **17** (2.79 g, 6.28 mmol) in CH_2Cl_2 (31.4 mL) and the reaction mixture was stirred at rt for 2 h. H_2O (100 mL) was added and the resultant mixture was basified with 2.0 M aq NaOH (100 mL) and extracted with CH_2Cl_2 (3 × 100 mL). The combined organics were washed with brine (100 mL), then dried, filtered and concentrated *in vacuo* to give a colourless oil (2.01 g).

Step 2. Et_3N (4.40 mL, 31.4 mmol) was added to the residue from the previous step (2.01 g) in CH_2Cl_2 (31.4 mL) at rt, and the resultant mixture was stirred at rt for 15 min. Allyl bromide (1.35 mL, 15.7 mmol) was added and the resultant mixture was stirred at rt for 16 h. H_2O (100 mL) was added and the reaction mixture was extracted with CH_2Cl_2 (3 × 100 mL). The combined organics were washed with brine (100 mL), then dried, filtered and concentrated *in vacuo* to give **18** as a white residue (2.28 g, 94%); ^1H NMR ($CDCl_3$, 400 MHz) 5.96–5.86 (m, 1H), 5.21–5.16 (m, 1H), 5.10–5.06 (m, 1H), 4.46 (dt, 1H, *J* 5.6, 2.0), 3.38–3.23 (m, 2H), 3.13–3.01 (m, 2H), 2.75–2.69 (m, 1H), 2.08−1.99 (m, 1H), 1.77–1.71 (m, 1H), 1.45 (s, 9H), 1.07–1.05 (m, 21H).

(2*S*,3*R*,*R*S,*E*)-*N*-[(1-allyl-3-triisopropylsilyloxypyrrolidin-2-yl)methylene]-*tert*-butylsulfinamide (*R*S)-20

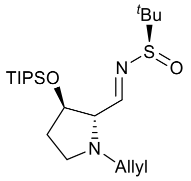

Step 1. DIBAL-H (1.0 M in CH_2Cl_2, 20.9 mL, 20.9 mmol) was added dropwise to a solution of **18** (1.59 g, 4.14 mmol) from the previous step in CH_2Cl_2 (26.1 mL) at −78 °C and the resultant mixture was stirred at −78 °C for 15 min. The reaction mixture was allowed to warm to rt and stirred at rt for 2 h. Satd aq NH_4Cl (20 mL) and satd aq Rochelle salt (50 mL) were added and the resultant mixture was stirred at rt for 2 h. The reaction mixture was filtered through Celite® (eluent CH_2Cl_2) and concentrated *in vacuo* to give a colourless oil (1.22 g).

Step 2. DMSO (1.49 mL, 20.9 mmol) was added dropwise to a stirred solution of $(COCl)_2$ (0.840 mL, 10.4 mmol) in CH_2Cl_2 (10.3 mL) and the resultant mixture was stirred at −78 °C for 20 min. A solution of the residue from the previous step (1.22 g) in CH_2Cl_2 (16.0 mL) was added dropwise and the reaction mixture was stirred at −78 °C for 30 min. Et_3N (4.40 mL, 31.3 mmol) was added and the reaction mixture was stirred at −78 °C for 30 min. The reaction mixture was allowed to warm to rt and stirred at rt for 16 h. H_2O (100 mL) was added and the resultant mixture was extracted with CH_2Cl_2 (3 × 150 mL). The combined organics were washed with brine (50 mL), dried, filtered and concentrated *in vacuo* to give **19** as a yellow residue.

Step 3. (*R*)-*tert*-Butylsulfinamide (1.14 g, 9.40 mmol) and $Ti(OEt)_4$ (2.38 g, 10.4 mmol) were added to a solution of **19** from the previous step in THF (26.1 mL) and the resultant mixture was stirred at rt for 24 h. H_2O (15 mL) was added and the resultant suspension was filtered through Celite® (eluent CH_2Cl_2). The combined filtrate was concentrated *in vacuo*. The residue was partitioned between CH_2Cl_2 (50 mL) and 10% aq citric acid (50 mL), and the organic layer was extracted with 10% aq citric acid (50 mL). The combined aqueous layers were extracted with CH_2Cl_2 (2 × 50 mL), and the combined organics were washed sequentially with satd aq $NaHCO_3$ (2 × 50 mL) and brine (50 mL), then dried, filtered and concentrated *in vacuo* to give (*R*S)-**20** as an orange oil (1.28 g, 74% from **18**); ^{1}H NMR ($CDCl_3$, 400 MHz) 7.89 (d, 1H, *J* 5.8), 5.85 (dddd, 1H, *J* 17.1, 10.1, 7.0, 6.0), 5.21–5.15 (m, 1H), 5.11–5.07 (m, 1H), 4.46 (dt, 1H, *J* 5.7, 2.7), 4.29–4.16 (m, 1H), 3.37–3.29 (m, 2H), 3.17–3.05 (m, 2H), 2.73–2.67 (m, 1H), 1.88–1.78 (m, 1H), 1.20 (s, 9H), 1.07–1.01 (m, 21H).

(2*S*,3*R*,*S*S,*E*)-*N*-[(1-allyl-3-triisopropylsilyloxypyrrolidin-2-yl)methylene]-*tert*-butylsulfinamide (*S*S)-21

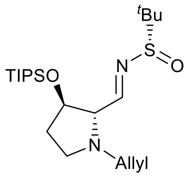

Step 1. DIBAL-H (1.0 M in CH_2Cl_2, 25.1 mL, 25.1 mmol) was added dropwise to a solution of **18** (2.28 g, 5.95 mmol) in CH_2Cl_2 (31.4 mL) at −78 °C and the resultant mixture was stirred at −78 °C for 15 min. The reaction mixture was allowed to warm to rt and stirred at rt for 2 h. Satd aq NH_4Cl (20 mL) and satd aq Rochelle salt (50 mL) were added and the resultant mixture was stirred at rt for 2 h. The reaction mixture was filtered through Celite® (eluent CH_2Cl_2) and concentrated *in vacuo* to give a colourless oil (1.79 g).

Step 2. DMSO (1.80 mL, 25.1 mmol) was added dropwise to a stirred solution of $(COCl)_2$ (1.02 mL, 12.6 mmol) in CH_2Cl_2 (20.4 mL) and the resultant mixture was stirred at −78 °C for 20 min. A solution of the residue from the previous step (1.79 g) in CH_2Cl_2 (11.0 mL) was added dropwise and the reaction mixture was stirred at −78 °C for 30 min. Et_3N (5.30 mL, 37.7 mmol) was added and the reaction mixture was stirred at −78 °C for 30 min. The reaction mixture was allowed to warm to rt and stirred at rt for 16 h. H_2O (100 mL) was added and the resultant mixture was extracted with CH_2Cl_2 (3 × 150 mL). The combined organics were washed with brine (50 mL), dried, filtered and concentrated *in vacuo* to give **19** as a yellow oil (1.95 g).

Step 3. (*S*)-*tert*-Butylsulfinamide (1.37 g, 11.3 mmol) and $Ti(OEt)_4$ (2.88 g, 12.6 mmol) were added to a solution of **19** (1.95 g) from the previous step in THF (31.4 mL) and the resultant mixture was stirred at rt for 24 h. H_2O (15 mL) was added and the resultant suspension was filtered through Celite® (eluent CH_2Cl_2). The combined filtrate was concentrated *in vacuo*. The residue was partitioned between CH_2Cl_2 (50 mL) and 10% aq citric acid (50 mL), and the organic layer was extracted with 10% aq citric acid (50 mL). The combined aqueous layers were extracted with CH_2Cl_2 (2 × 50 mL), and the combined organics were washed sequentially with satd aq $NaHCO_3$ (2 × 50 mL) and brine (50 mL), then dried, filtered and concentrated *in vacuo* to give (*S*S)-**21** as an orange oil (1.73 g, 70% from **18**); ^1H NMR ($CDCl_3$, 400 MHz) 7.91 (d, 1H, *J* 5.0), 5.88 (dddd, 1H, *J* 17.2, 10.1, 7.1, 5.9), 5.21–5.20 (m, 1H), 5.16–5.07 (m, 1H), 4.43 (dt, 1H, *J* 5.1, 2.3), 4.29–4.16 (m, 1H), 3.46–3.39 (m, 2H), 3.19–3.08 (m, 2H), 1.95 (dddd, 1H, *J* 12.8, 11.0, 7.3, 5.5), 1.87–1.77 (m, 1H), 1.20 (s, 9H), 1.08–1.03 (m, 21H).

O-tert-Butoxycarbonyl methyl glycolate 22

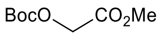

DMAP (1.35 g, 11.1 mmol) and Boc_2O (36.0 g, 166 mmol) were added to a solution of methyl glycolate (10.0 g, 111 mmol) in MeCN (444 mL) and the resultant mixture was stirred at rt for 3 days. Brine (100 mL) was added and the resultant mixture was extracted with Et_2O (2 × 400 mL). The combined organics were dried, filtered and concentrated *in vacuo*. Purification via flash column chromatography (eluent 30–40 °C petrol/Et_2O, 10:1 to 0:1) gave **22** as a colourless oil (20.1 g, 99%); ^{1}H NMR ($CDCl_3$, 400 MHz) 4.57 (s, 2H), 3.78 (s, 3H), 1.50 (s, 9H).

Enolate Trapping Experiments

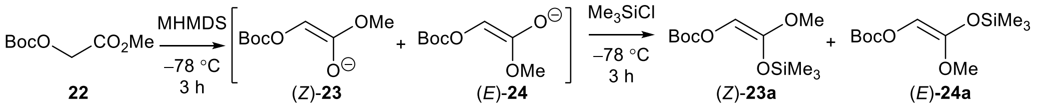

Method A. TMSCl (90 μL, 0.70 mmol) was added to LiHMDS (1.0 M in THF, 0.75 mL, 0.75 mmol) at −78 °C and the resultant solution was stirred at −78 °C for 5 min. A solution of **22** (95 mg, 0.500 mmol) in THF (1.8 mL) was added and the resultant solution was stirred at −78 °C for 2 h. The reaction mixture was stirred at rt for 30 mins then 30–40 °C petrol (3 mL) was added. The resultant suspension was filtered through Celite® (eluent CH_2Cl_2) and concentrated *in vacuo* to give a 90:10 mixture of (*Z*)-**23a** and (*E*)-**24a**; ^{1}H NMR ($CDCl_3$, 500 MHz) [selected peaks] 3.52 (s, 2.7H), 3.62 (s, 0.3H), 5.98 (s, 0.9H), 6.00 (s, 0.1H).

Method B. TMSCl (90 μL, 0.70 mmol) was added to NaHMDS (2.0 M in THF, 0.38 mL, 0.75 mmol) at −78 °C and the resultant solution was stirred at −78 °C for 5 min. A solution of **22** (95 mg, 0.500 mmol) in THF (1.8 mL) was added and the resultant solution was stirred at −78 °C for 2 h. The reaction mixture was stirred at rt for 30 mins then 30–40 °C petrol (3 mL) was added. The resultant suspension was filtered through Celite® (eluent CH_2Cl_2) and concentrated *in vacuo* to give a 75:25 mixture of (*Z*)-**23a** and (*E*)-**24a**; ^{1}H NMR ($CDCl_3$, 500 MHz) 6.00 (s, 0.25H), 5.98 (s, 0.75H), 3.62 (s, 0.75H), 3.52 (s, 2.25H).

Methyl (2*R*,3*S*,2′*S*,3′*R*,*R*S)-2-(*tert*-butoxycarbonyl)oxy-3-(*tert*-butylsulfinyl)amino-3-(1′-allyl-3′-triisopropylsilyloxypyrrolidin-2′-yl)propanoate 25

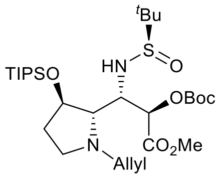

LiHMDS (1 M in THF, 17.8 mL, 17.8 mmol) was added to a solution of **22** (1.48 g, 17.8 mmol) in THF (64.0 mL) at −78 °C and the resultant mixture was stirred at −78 °C for 1 h. A solution of (*R*S)-**20** (1.48 g, 3.57 mmol) in THF (15.8 mL) was added dropwise and the reaction mixture was stirred at −78 °C for 3 h. Satd aq NH_4Cl (50 mL) was added and the resultant mixture was extracted with CH_2Cl_2 (3 × 250 mL). The combined organics were washed with brine (250 mL), dried, filtered and concentrated *in vacuo*. Purification via flash column chromatography (eluent 30–40 °C petrol/Et_2O, 2:1 to 1:1) gave **25** as a yellow oil (1.79 g, 83%); $[\alpha]_D^{25}$ −63.1 (*c* 1.0 in $CHCl_3$); ν_{max} 1749; ^{1}H NMR ($CDCl_3$, 400 MHz) 5.84 (dddd, 1H, *J* 17.3, 10.2, 7.2, 5.4), 5.21–5.03 (m, 3H), 4.87 (d, 1H, *J* 4.0), 4.33 (dt, 1H, *J* 4.4, 2.0), 3.72 (s, 3H), 3.59 (dt, 1H, *J* 5.6, 4.0), 3.47 (ddt, 1H, *J* 14.1, 5.4, 1.7), 3.21 (dt, 1H, *J* 14.1, 7.2, 1.1), 3.03 (ddd, 1H, *J* 9.4, 7.4, 2.9), 2.93–2.89 (m, 1H), 2.73 (ddd, 1H, *J* 10.3, 9.4, 6.1), 1.90 (dddd, 1H, *J* 12.8, 10.3, 7.4, 4.4), 1.70 (ddd, 1H, *J* 12.8, 6.1, 2.8), 1.45 (s, 9H), 1.18 (s, 9H), 1.09–0.99 (m, 21H); $^{13}C\{^1H\}$ NMR ($CDCl_3$, 100 MHz) 168.6, 152.8, 136.0, 116.6, 83.0, 77.8, 76.2, 72.8, 59.1, 56.8, 56.0, 52.2, 51.6, 34.3, 27.6, 22.7, 18.1, 18.1, 12.1; HRMS (ESI$^+$) $C_{29}H_{57}N_2O_7SSi^+$ ($[M+H]^+$) requires 605.3650; found 605.3640.

Methyl (2*S*,3*R*,2′*S*,3′*R*,*S*S)-2-(*tert*-butoxycarbonyl)oxy-3-(*tert*-butylsulfinyl)amino-3-(1′-allyl-3′-triisopropylsilyloxypyrrolidin-2′-yl)propanoate 26

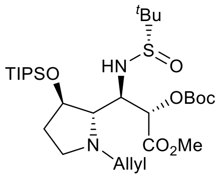

NaHMDS (2 M in THF, 8.8 mL, 17.6 mmol) was added to a solution of **22** (3.35 g, 17.6 mmol) in THF (64 mL) at −78 °C and the resultant mixture was stirred at −78 °C for 1 h. A solution of (*S*S)-**21** (1.46 g, 3.52 mmol) in THF (12.6 mL) was added dropwise and the reaction mixture was stirred at −78 °C for 3 h. Satd aq NH_4Cl (50 mL) was added and the resultant mixture was extracted with CH_2Cl_2 (3 × 250 mL). The combined organics were washed with brine (250 mL), dried, filtered and concentrated *in vacuo*. Purification via flash column chromatography (eluent 30–40 °C petrol/Et_2O, 2:1 to 1:1) gave **26** as a yellow oil (2.04 g, 76%); $[\alpha]_D^{25}$ +22.2 (*c* 1.0 in $CHCl_3$); ν_{max} 1753; ^{1}H NMR ($CDCl_3$, 400 MHz) 5.81 (dddd, 1H, *J* 17.4, 10.1, 7.5, 5.0), 5.49

(d, 1H, *J* 1.7), 5.14–5.09 (m, 1H), 5.02–4.99 (m, 1H), 4.79 (d, 1H, *J* 3.0), 3.70 (s, 3H), 3.40–3.30 (m, 3H), 3.19–3.13 (m, 1H), 3.07 (ddd, 1H, *J* 8.8, 7.6, 1.2), 2.77–2.70 (m, 2H), 1.97–1.82 (m, 2H), 1.48 (s, 9H), 1.16–1.02 (m, 21H) overlapping 1.14 (s, 9H); $^{13}C\{^1H\}$ NMR ($CDCl_3$, 100 MHz) 169.8, 152.6, 136.8, 116.2, 83.2, 75.1, 74.4, 74.1, 61.8, 61.1, 56.5, 52.2, 52.1, 33.9, 27.8, 22.8, 18.4, 18.2, 12.1; HRMS (ESI^+) $C_{29}H_{57}N_2O_7SSi^+$ ($[M+H]^+$) requires 605.3650; found 605.3663.

(1*R*,2*S*,7*R*,7a*S*,*S*S)-1-(*tert*-Butylsulfinyl)amino-2-(*tert*-butoxycarbonyl)oxy-7-triisopropylsilyloxypyrrolizin-3-one 28

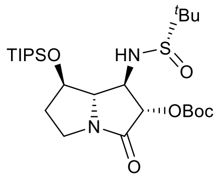

$Pd(PPh_3)_4$ (2.02 g, 1.75 mmol) was added to a degassed solution of **26** (4.23 g, 6.99 mmol) and DMBA (3.27 g, 20.1 mmol) in CH_2Cl_2 (70 mL) under an argon atmosphere. The resultant mixture was stirred in the dark at rt for 18 h, then filtered through Celite® (eluent CH_2Cl_2), and the filtrate concentrated *in vacuo*. Purification via flash column chromatography (eluent 30–40 °C petrol/acetone, 7.5:1, then 30–40 °C petrol/acetone, 5:1) gave **28** yellow oil (3.89 g, quant); $[\alpha]_D^{25}$ −18.9 (*c* 1.0 in $CHCl_3$); ν_{max} 1732; ^1H NMR ($CDCl_3$, 500 MHz) 5.41 (dd, 1H, *J* 8.9, 1.2), 4.43 (td, 1H, *J* 4.8, 3.5), 3.85 (ddd, 1H, *J* 11.8, 7.1, 5.1), 3.78 (ddd, 1H, *J* 8.9, 8.0, 4.8), 3.69 (d, 1H, *J* 4.8), 3.53 (dd, 1H, *J* 8.0, 3.5), 3.20 (dtd, 1H, *J* 11.8, 7.6, 1.3), 1.95–1.90 (m, 2H), 1.48 (s, 9H), 1.20 (s, 9H), 1.15–1.04 (m, 21H); $^{13}C\{^1H\}$ NMR ($CDCl_3$, 125 MHz) 168.2, 152.4, 83.3, 79.6, 74.9, 69.6, 62.4, 56.2, 42.0, 35.3, 27.7, 22.4, 18.2, 18.0, 12.1; HRMS (ESI^+) $C_{25}H_{49}N_2O_6SSi^+$ ($[M+H]^+$) requires 533.3075; found 533.3076.

(1*R*,2*S*,7*R*,7a*S*,*S*S)-1-(*tert*-Butylsulfinyl)amino-2-(*tert*-butoxycarbonyl)oxy-7-hydroxypyrrolizin-3-one 29

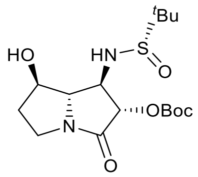

TBAF (1.0 M in THF, 3.30 mL, 3.30 mmol) was added to a solution of **28** (440 mg, 0.828 mmol) in THF (4.1 mL), and the resultant mixture was stirred at rt for 2 h. The resultant solution was concentrated *in vacuo*. Purification via flash column chromatography (eluent 30–40 °C petrol/acetone, 3:1 to 1:1) gave **29** as colourless crystals (299 mg, 96%); mp 184–188 °C; $[\alpha]_D^{25}$ −21.9 (*c* 1.0 in $CHCl_3$); ν_{max} 3237, 1750, 1716; ^1H

NMR ($CDCl_3$, 400 MHz) 5.40 (br d, 1H, *J* 10.0), 5.16 (d, 1H, *J* 1.5), 4.20 (br q, 1H, *J* 7.2), 3.88 (d, 1H, *J* 11.2), 3.72 (ddd, 1H, *J* 11.2, 10.0, 7.7), 3.63–3.54 (m, 1H), 3.41 (dd, 1H, *J* 7.7, 6.4), 3.31 (dddd, 1H, *J* 11.4, 9.6, 3.1, 1.4), 2.44 (dtd, 1H, *J* 13.3, 7.7, 3.1), 2.14–2.01 (m, 1H), 1.48 (s, 9H), 1.26 (s, 9H); $^{13}C\{^1H\}$ NMR ($CDCl_3$, 100 MHz) 165.6, 152.6, 83.6, 78.3, 74.5, 69.5, 66.2, 56.7, 40.2, 33.7, 27.6, 22.5; HRMS (ESI$^+$) $C_{16}H_{29}N_2O_6S^+$ ($[M+H]^+$) requires 377.1741; found 377.1737.

(1*R*,2*R*,4*S*,7*R*,7a*S*,*S*S)-1-(*tert*-Butylsulfinyl)amino-2-(*tert*-butoxycarbonyl)oxy-7-hydroxypyrrolizine borane complex 30·BH_3

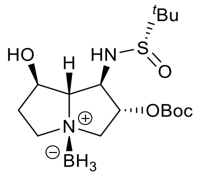

BH_3·SMe_2 (0.25 mL, 2.65 mmol) was added to a solution of **29** (100 mg, 0.265 mmol) in THF (2.7 mL) and the resultant mixture was stirred at rt for 16 h. MeOH (1 mL) was added and the resultant mixture was concentrated *in vacuo*. Purification via flash column chromatography (eluent 30–40 °C petrol/acetone, 3:1) gave **30**·BH_3 as a colourless crystalline solid (49 mg, 51%); mp 196–197 °C (dec); $[\alpha]_D^{25}$ −2.2 (*c* 1.0 in $CHCl_3$); ν_{max} 3260, 2380, 1747; ^1H NMR ($CDCl_3$, 400 MHz) 5.28 (dt, 1H, *J* 7.7, 6.4), 4.93 (br s, 1H), 4.65 (d, 1H, *J* 9.2), 4.41 (td, 1H, *J* 5.4, 3.6), 3.69–3.50 (m, 4H), 3.07–2.98 (m, 1H), 2.87 (dd, 1H, *J* 11.6, 7.8), 2.23–2.11 (m, 2H), 1.87 (br s, 3H), 1.45 (s, 9H), 1.24 (s, 9H); $^{13}C\{^1H\}$ NMR ($CDCl_3$, 100 MHz) 152.1, 84.0, 83.5, 78.2, 75.5, 65.5, 63.7, 63.1, 56.8, 32.5, 27.8, 22.8; ^{11}B NMR ($CDCl_3$, 160 MHz) −6.2; HRMS (ESI$^+$) $C_{16}H_{33}{}^{11}BN_2NaO_5S^+$ ($[M+Na]^+$) requires 398.2096; found 398.2093.

(1*R*,2*R*,7*R*,7a*S*,*S*S)-1-(*tert*-Butylsulfinyl)amino-2-(*tert*-butoxycarbonyl)oxy-7-hydroxypyrrolizine 30

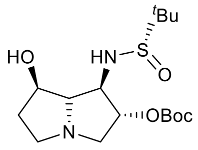

Method A. BH_3·THF solution (1.0 M in THF, 72.5 mL, 72.5 mmol) was added to a solution of **29** (2.73 g, 7.25 mmol) in THF (72.5 mL) and the resultant mixture was stirred at rt for 2 h. MeOH (145 mL) was added and the resultant mixture was heated at 70 °C for 16 h. The resultant solution was concentrated *in vacuo*. Purification via flash column chromatography (eluent $CHCl_3$/MeOH/35% aq NH_4OH, 20:1:0.1) gave **30** as colourless crystals (2.07 g, 79%); mp 161–164 °C; $[\alpha]_D^{25}$ +23.1(*c* 1.0 in $CHCl_3$); ν_{max} 3656, 1745; ^1H NMR ($CDCl_3$, 400 MHz) 5.11 (td, 1H, *J* 8.8, 6.6), 4.31 (dt, 1H, *J* 8.4, 5.9), 3.68 (d, 1H, *J* 10.2), 3.50–3.39 (m, 2H), 3.20 (ddd, 1H, *J* 9.8, 6.8, 3.0), 3.06 (dd, 1H, *J* 8.5, 5.3), 2.63–2.53 (m, 2H), 2.29–2.19 (m, 1H), 1.97 (dddd,

1H, *J* 12.5, 10.2, 8.4, 6.9), 1.45 (s, 9H), 1.22 (s, 9H); $^{13}C\{^{1}H\}$ NMR ($CDCl_3$, 100 MHz) 152.9, 82.9, 79.4, 76.2, 75.8, 66.2, 56.4, 56.0, 53.9, 35.2, 27.8, 22.7; HRMS (ESI^+) $C_{16}H_{31}N_2O_5S^+$ ($[M+H]^+$) requires 363.1948; found 363.1949.

Method B. A solution of **30**·BH_3 (18 mg, 50 μmol) in MeOH-d_4 (0.50 mL) was heated at 70 °C for 16 h. The resultant solution was concentrated *in vacuo* to give **30** as a white residue (17 mg, quant).

(1*R*,2*R*,7*S*,7a*S*,*S*S)-1-(*tert*-Butylsulfinyl)amino-2,7-epoxypyrrolizidine 32

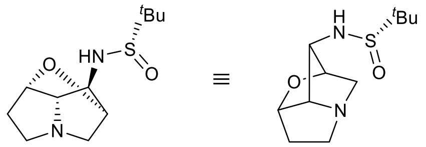

Step 1. Et_3N (0.460 mL, 3.31 mmol) was added to a solution of **30** (300 mg, 0.828 mmol) and Ms_2O (289 mg, 1.66 mmol) in CH_2Cl_2 (8.3 mL) and the resultant mixture was stirred at 0 °C for 1 h. H_2O (10 mL) was added and the resultant solution was extracted with CH_2Cl_2 (3 × 10 mL). The combined organics were washed with brine (30 mL), dried, filtered and concentrated *in vacuo* (at 0 °C) to give **31** as a yellow oil (528 mg).

Step 2. K_2CO_3 (686 mg, 4.97 mmol) was added to a solution of **31** (528 mg) from the previous step in MeOH (16.6 mL). The resultant suspension was heated at 70 °C for 20 h, then concentrated *in vacuo*. Purification via flash column chromatography (eluent $CHCl_3$/MeOH/35% aq NH_4OH, 20:1:0.1) gave **32** as a yellow oil (136 mg, 67%); ^1H NMR ($CDCl_3$, 400 MHz) 4.42 (d, 1H, *J* 2.2), 4.38 (dd, 1H, *J* 4.5, 1.9), 3.88 (dd, 1H, *J* 2.2, 1.0), 3.58–3.51 (m, 1H), 3.44–3.39 (m, 1H), 3.10 (ddd, 1H, *J* 12.8, 8.6, 3.8), 3.00 (ddd, 1H, *J* 12.8, 9.5, 7.1), 2.42 (d, 1H, *J* 11.5), 2.19 (br s, 1H), 2.06 (ddd, 1H, *J* 14.0, 8.6, 7.1), 1.94 (ddt, 1H, *J* 14.0, 9.5, 3.8), 1.26 (s, 9H).

(1*R*,2*R*,7*S*,7a*S*)-1-Amino-2,7-epoxypyrrolizidine dihydrochloride [norloline dihydrochloride] 33·2HCl

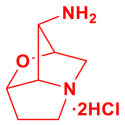

Step 1. Et_3N (1.25 mL, 9.00 mmol) was added to a solution of **30** (817 mg, 2.25 mmol) and Ms_2O (786 mg, 4.51 mmol) in CH_2Cl_2 (22.5 mL) and the resultant mixture was stirred at 0 °C for 1 h. H_2O (50 mL) was added and the resultant solution was extracted with CH_2Cl_2 (3 × 50 mL). The combined organics were washed with brine (100 mL), dried, filtered and concentrated *in vacuo* (at 0 °C) to give **31** as a yellow oil (1.56 g).

Step 2. K_2CO_3 (622 mg, 4.50 mmol) was added to a solution of **31** (427 mg) from the previous step in MeOH (15.0 mL) and heated to 70 °C in three sealed vials for 20 h. Silica gel (2 g) was added to each vial and the resultant mixtures were combined and concentrated *in vacuo*. Filtration of the combined resultant solid through a pad of silica gel (eluent $CHCl_3$/MeOH/35% aq NH_4OH, 10:1:0.1) gave **32** as an orange oil (680 mg).

Step 3. HCl (1.25 M in MeOH, 12.0 mL, 9.60 mmol) was added to **32** (227 mg) from the previous step and heated to 70 °C in three sealed vials for 20 h. The resultant mixtures were combined and concentrated *in* vacuo. H_2O (10 mL) was added and the resultant solution was extracted with CH_2Cl_2 (3 × 10 mL). The combined aqueous extracts were concentrated *in vacuo* to give **33**·2HCl as a brown solid (407 mg, 85%); mp 168–171 °C (dec); $[\alpha]_D^{25}$ +7.0 (*c* 1.0 in H_2O); ^{1}H NMR (D_2O, 400 MHz) 4.91–4.85 (m, 3H), 4.40 (d, 1H, *J* 2.6), 4.29 (dd, 1H, *J* 13.8, 1.2), 3.97–3.82 (m, 2H), 3.71 (d, 1H, *J* 13.8), 2.55 (dddd, 1H, *J* 15.1, 9.2, 6.0, 4.9), 2.49–2.39 (m, 1H); ^{13}C{^{1}H} NMR (D_2O, 100 MHz) 83.4, 75.1, 73.7, 64.4, 58.2, 58.2, 31.9; HRMS (ESI$^+$) $C_7H_{13}N_2O^+$ ($[M+H]^+$) requires 141.1022; found 141.1021.

(1*R*,2*R*,7*S*,7a*S*)-1-Amino-2,7-epoxypyrrolizidine [norloline] 33

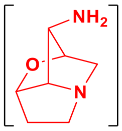

NaOH (88 mg, 2.20 mmol) was added to a suspension of **33**·2HCl (43 mg, 0.202 mmol) in $CDCl_3$ (2.0 mL) and the mixture was stirred at rt for 30 min. The resultant solution was filtered and analysed by ^{1}H and ^{13}C NMR spectroscopy; ^{1}H NMR ($CDCl_3$, 400 MHz) 4.40 (dd, 1H, *J* 4.5, 1.8), 3.83 (dd, 1H, *J* 2.6, 1.1), 3.60–3.55 (m, 1H), 3.48 (d, 1H, *J* 11.7), 3.09 (ddd, 1H, *J* 12.6, 8.4, 3.4), 3.03 (dd, 1H, *J* 1.8, 1.6), 2.92 (dddd, 1H, *J* 12.6, 9.5, 7.3, 0.8), 2.41 (dt, 1H, *J* 11.7, 1.6), 2.05 (ddd, 1H, *J* 14.2, 8.4, 7.3), 1.96 (dddd, 1H, *J* 14.2, 9.5, 4.5, 3.4); ^{13}C{^{1}H} NMR ($CDCl_3$, 100 MHz) 81.7, 76.2, 71.9, 60.8, 60.3, 54.6, 34.2.

(1*R*,2*R*,7*S*,7a*S*)-1-Acetamido-2,7-epoxypyrrolizidine [*N*-acetyl norloline] 34

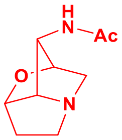

Et_3N (0.157 mL, 1.13 mmol) and Ac_2O (50.0 µL, 0.563 mmol) were added to a suspension of **30**·2HCl (43 mg, 0.202 mmol) in CH_2Cl_2 (2.8 mL) and the resultant mixture was stirred at rt for 2 h. The resultant solution was concentrated *in vacuo*. Purification via flash column chromatography (eluent $CHCl_3$/MeOH/35%

aq NH_4OH, 15:1:0.1) gave a yellow oil. 2.0 M aq NaOH (5 mL) was added and the resultant solution was extracted with $CHCl_3$ (5 × 5 mL). The combined organics were washed with brine (50 mL), dried, filtered and concentrated *in vacuo* to give **34** as a colourless oil (34 mg, 67%); $[\alpha]_D^{25}$ +40.0 (*c* 1.0 in $CHCl_3$), $[\alpha]_D^{20}$ +54.6 (*c* 0.5 in $CHCl_3$); ν_{max} 3434, 1661; ^1H NMR ($CDCl_3$, 400 MHz) 6.30 (br s, 1H), 4.46 (dd, 1H, *J* 4.6, 1.9), 4.47–4.41 (m, 1H), 4.19 (d, 1H, *J* 1.4), 3.32 (dd, 1H, *J* 11.9, 1.3), 3.16–3.12 (m, 1H), 3.10 (dd, 1H, *J* 8.6, 3.7), 2.93 (ddd, 1H, *J* 12.9, 9.4, 7.3), 2.45 (d, 1H, *J* 11.9), 2.10 (ddd, 1H, *J* 14.3, 8.6, 7.6), 2.06–2.01 (m, 1H), 1.99 (s, 3H); $^{13}C\{^1H\}$ NMR ($CDCl_3$, 100 MHz) 170.4, 80.9, 73.8, 69.6, 60.9, 57.6, 54.6, 33.8, 23.3; HRMS (ESI$^+$) $C_9H_{15}N_2O_2^+$ ([M+H]$^+$) requires 183.1128; found 183.1130.

(1*R*,2*R*,7*S*,7a*S*)-1-(*N*,*N*-Dimethylamino)-2,7-epoxypyrrolizidine·dihydrochloride [*N*-methyl loline dihydrochloride] 35·2HCl

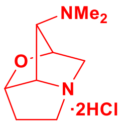

Formaldehyde (35% in H_2O, 0.26 mL, 3.00 mmol) was added to a solution of **33**·2HCl (40 mg, 0.188 mmol) in MeCN (6.3 mL) and the resultant mixture was stirred at 0 °C for 10 min. $NaBH_3CN$ (24 mg, 0.376 mmol) was added at 0 °C and the resultant mixture was stirred at rt for 16 h. The resultant solution was concentrated *in vacuo*. Purification via flash column chromatography (eluent $CHCl_3$/MeOH 5:1) followed by addition of HCl (1.25 M in MeOH, 12 mL) gave **35**·2HCl as a colourless oil (30 mg, 66%); $[\alpha]_D^{25}$ +7.2 (*c* 1.0 in H_2O); ν_{max} 3381; ^1H NMR (D_2O, 400 MHz) 5.03–4.96 (m, 2H), 4.89 (dd, 1H, *J* 4.8, 2.2), 4.50–4.45 (m, 1H), 4.29 (dd, 1H, *J* 14.2, 1.2), 3.96–3.78 (m, 2H), 3.73 (d, 1H, *J* 14.2), 3.11 (s, 6H), 2.52 (ddt, 1H, *J* 15.3, 9.9, 4.8), 2.41 (ddd, 1H, *J* 15.3, 8.9, 7.4); $^{13}C\{^1H\}$ NMR (D_2O, 100 MHz) 84.0, 74.1, 73.8, 72.2, 64.3, 58.6, 47.3, 31.8; HRMS (ESI$^+$) $C_9H_{17}N_2O^+$ ([M+H]$^+$) requires 169.1335; found 169.1336.

(1*R*,2*R*,7*S*,7a*S*)-1-(*N*,*N*-Dimethylamino)-2,7-epoxypyrrolizidine·[*N*-methyl loline] 35

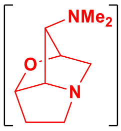

NaOH (100 mg, 2.50 mmol) was added to a suspension of **35**·2HCl (30 mg, 0.125 mmol) in $CDCl_3$ (2.0 mL) and the mixture was stirred at rt for 30 min. The resultant solution was filtered and analysed by ^1H and ^{13}C NMR spectroscopy; ^1H NMR ($CDCl_3$, 400 MHz) 4.42 (dd, 1H, *J* 4.5, 1.9), 4.00 (d, 1H, *J* 1.3), 3.54 (dt, 1H, *J* 11.0, 0.8), 3.18 (t, 1H, *J* 1.5), 3.06 (ddd, 1H, *J* 12.4, 8.6, 3.6), 2.99–2.91 (m, 1H), 2.70 (d, 1H, *J* 1.5),

2.36 (d, 1H, *J* 11.0), 2.29 (s, 6H), 2.03 (ddd, 1H, *J* 14.2, 8.6, 7.1), 1.96–1.88 (m, 1H); $^{13}C\{^{1}H\}$ NMR ($CDCl_3$, 100 MHz) 82.4, 74.7, 74.5, 69.7, 61.6, 54.7, 44.8, 33.8.

(1*R*,2*R*,7*S*,7a*S*)-1-(*tert*-Butoxycarbonyl)amino-2,7-epoxypyrrolizidine [*N*-Boc norloline] 36

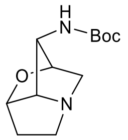

Boc_2O (102 mg, 0.469 mmol) and K_2CO_3 (129 mg, 0.940 mmol) were added to a suspension of **33**·2HCl (40 mg, 0.188 mmol) in MeOH (3.8 mL) and the mixture was stirred at rt for 24 h. The resultant solution was concentrated *in vacuo*. Purification via flash column chromatography (eluent $CHCl_3$/MeOH/35% aq NH_4OH, 20:1:0.1) gave **36** as a colourless oil (15 mg, 33%); $[\alpha]_D^{25}$ +24.3 (*c* 1.0 in $CHCl_3$); $[\alpha]_D^{25}$ +20.1 (*c* 0.35 in $CHCl_3$); ν_{max} 1706; ^{1}H NMR ($CDCl_3$, 400 MHz) 5.87 (br s, 1H), 4.44 (dd, 1H, *J* 4.5, 1.9), 4.21 (d, 1H, *J* 7.2), 4.16–4.11 (m, 1H), 3.35 (d, 1H, *J* 11.8), 3.14–3.03 (m, 2H), 2.92 (ddd, 1H, *J* 12.9, 9.4, 7.2), 2.40 (d, 1H, *J* 11.8), 2.12–1.91 (m, 2H), 1.43 (s, 9H); $^{13}C\{^{1}H\}$ NMR ($CDCl_3$, 100 MHz) 155.6, 81.1, 79.5, 74.1, 69.6, 61.0, 58.5, 54.6, 33.8, 28.4; HRMS (ESI^+) $C_{12}H_{21}N_2O_3^+$ ($[M+H]^+$) requires 241.1547; found 241.1546.

(1*R*,2*R*,7*S*,7a*S*)-1-[*N*-Methylamino]-2,7-epoxypyrrolizidine dihydrochloride [loline dihydrochloride] 37·2HCl

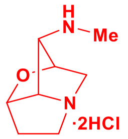

$LiAlH_4$ (2.0 M in THF, 0.19 mL, 0.375 mmol) was added to a solution of **36** (15 mg, 62.4 μmol) in THF (1.25 mL) and the resultant solution was heated at 70 °C for 4 h. 2.0 M aq NaOH (0.5 mL) was added and the resultant mixture was stirred at rt for 30 min. The resultant mixture was filtered through Celite® (eluent CH_2Cl_2), then silica gel (500 mg) was added and the resultant mixture was concentrated *in vacuo*. Purification via flash column chromatography (eluent $CHCl_3$/MeOH 4:1) followed by addition of HCl (1.25 M in MeOH, 5 mL) gave **37**·2HCl as a colourless oil (5 mg, 35%); $[\alpha]_D^{25}$ +3.3 (*c* 0.31 in H_2O); ν_{max} 2965, 2924; ^{1}H NMR (D_2O, 400 MHz) 4.98−4.94 (m, 2H), 4.90 (dd, 1H, *J* 4.9, 2.3), 4.40 (d, 1H, *J* 2.5), 4.30 (dd, 1H, *J* 14.0, 1.2), 3.98–3.82 (m, 2H), 3.74 (d, 1H, *J* 14.0), 2.97 (s, 3H), 2.56 (ddt, 1H, *J* 15.0, 10.0, 4.9), 2.51–2.39 (m, 1H); $^{13}C\{^{1}H\}$ NMR (D_2O, 100 MHz) 83.4, 74.1, 72.2, 66.0, 64.3, 58.2, 36.4, 31.6; HRMS (ESI^+) $C_8H_{15}N_2O^+$ ($[M+H]^+$) requires 155.1179; found 155.1178.

(1*R*,2*R*,7*S*,7a*S*)-1-[*N*-Methylamino]-2,7-epoxypyrrolizidine [loline] 37

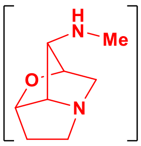

NaOH (18 mg, 0.440 mmol) was added to a suspension of **37**·2HCl (5 mg, 22.0 µmol) in $CDCl_3$ (1.0 mL) and the resultant suspension was stirred at rt for 30 min. The resultant solution was filtered and analysed by ^{1}H and ^{13}C NMR spectroscopy; ^{1}H NMR ($CDCl_3$, 500 MHz) 4.41 (dd, 1H, *J* 4.5, 1.7), 4.03 (t, 1H, *J* 1.8), 3.41 (d, 1H, *J* 11.6), 3.36–3.32 (m, 1H), 3.15 (dd, 1H, *J* 1.7, 1.5), 3.08 (ddd, 1H, *J* 12.6, 8.5, 3.4), 2.93 (ddd, 1H, *J* 12.6, 9.5, 7.2), 2.49 (s, 3H), 2.42 (d, 1H, *J* 11.6), 2.05 (ddd, 1H, *J* 14.2, 8.5, 7.2), 2.01–1.91 (m, 1H); ^{13}C{^{1}H} NMR ($CDCl_3$, 125 MHz) 81.8, 74.1, 69.6, 68.3, 61.2, 54.7, 35.2, 34.1.

Methyl (2*R*,3*S*,2′*S*,3′*R*,*R*s)-2-(*tert*-butoxycarbonyl)oxy-3-(*tert*-butylsulfinyl)amino-3-(3′-triisopropylsilyloxypyrrolidin-2′-yl)propanoate 38 and (1*S*,2*R*,7*R*,7a*S*,*R*s)-1-(*tert*-Butylsulfinyl)amino-2-(*tert*-butoxycarbonyl)oxy-7-triisopropylsilyloxypyrrolizin-3-one 39

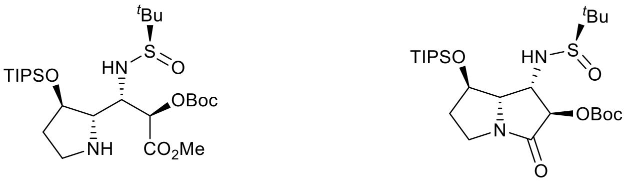

38 **39**

Pd(PPh_3)$_4$ (152 mg, 0.132 mmol) was added to a degassed solution of **25** (400 mg, 0.661 mmol) and DMBA (309 mg, 1.98 mmol) in CH_2Cl_2 (6.60 mL) under an argon atmosphere. The resultant mixture was stirred in the dark at rt for 18 h, then filtered through Celite® (eluent CH_2Cl_2). The filtrate was concentrated *in vacuo* to give an ~70:30 mixture of **38** and **39**, respectively. Purification via flash column chromatography (eluent 30–40 °C petrol/Et_2O, 1:2) gave **38** as a yellow oil (167 mg, 45%); $[\alpha]_D^{25}$ −38.5 (*c* 1.0 in $CHCl_3$); ν_{max} 3237, 1749, 1703, 1675; ^{1}H NMR ($CDCl_3$, 400 MHz) 5.11 (s, 1H), 4.82 (d, 1H, *J* 4.1), 4.42 (dt, 1H, *J* 5.8, 3.4), 3.81–3.76 (m, 1H), 3.75–3.72 (m, 4H), 3.27 (d, 1H, *J* 3.4), 3.14 (td, 1H, *J* 9.1, 7.1), 2.99 (ddd, 1H, *J* 9.6, 7.3, 4.3), 1.95 (dddd, 1H, *J* 13.0, 8.7, 7.4, 5.8), 1.72 (ddt, 1H, *J* 11.1, 7.4, 3.9), 1.47 (s, 9H), 1.20 (s, 9H), 1.09–1.02 (m, 21H); ^{13}C{^{1}H} NMR ($CDCl_3$, 100 MHz) 168.7, 152.9, 83.4, 77.6, 76.4, 66.9, 57.9, 56.4, 52.3, 44.8, 35.6, 27.8, 22.9, 18.2, 18.1, 12.3; HRMS (ESI$^+$) $C_{26}H_{53}N_2O_7SSi^+$ ([M+H]$^+$) requires 565.3337; found 565.3330. Further elution gave **39** as colourless crystals (59 mg, 17%); mp 170–173 °C; $[\alpha]_D^{25}$ −11.7 (*c* 1.0 in $CHCl_3$); ν_{max} 1749, 1703; ^{1}H NMR ($CDCl_3$, 400 MHz) 4.97 (d, 1H, *J* 1.8), 4.52 (td, 1H, *J* 5.2, 4.2), 4.19–4.11 (m, 2H), 3.74 (dd, 1H, *J* 12.0, 6.1), 3.41 (d, 1H, *J* 4.6), 3.27–3.19 (m, 1H), 2.06–1.99 (m, 2H), 1.50 (s, 9H), 1.20 (s, 9H), 1.10–1.04 (m, 21H); ^{13}C{^{1}H} NMR ($CDCl_3$, 100 MHz) 168.1, 152.1, 83.8, 81.8, 70.8, 70.6, 56.5,

56.4, 40.8, 36.9, 27.7, 22.4, 18.1, 12.2; HRMS (ESI^+) $C_{25}H_{49}N_2O_6SSi^+$ ($[M+H]^+$) requires 533.3075; found 533.3073.

(1*R*,2*S*,7*R*,7a*S*,*S*S)-1-(*tert*-Butylsulfinyl)amino-2-(*tert*-butoxycarbonyl)oxy-7-triisopropylsilyloxypyrrolizin-3-one 28 and (1*R*,2*R*,7*R*,7a*S*,*S*S)-1-(*tert*-butylsulfinyl)amino-2-(*tert*-butoxycarbonyl)oxy-7-triisopropylsilyloxypyrrolizin-3-one 40

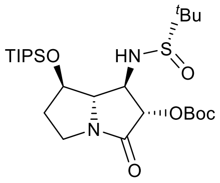

28

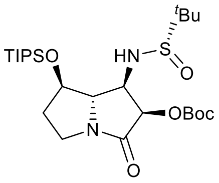

40

Step 1. LiHMDS (1 M in THF, 15.9 mL, 15.9 mmol) was added to a solution of **22** (3.02 g, 15.9 mmol) in THF (57.1 mL) at −78 °C and the resultant mixture was stirred at −78 °C for 1 h. A solution of (S_S)-**21** (1.78 g) in THF (18.9 mL) was added dropwise and the reaction mixture was stirred at −78 °C for 3 h. Satd aq NH_4Cl (50 mL) was added and the resultant mixture was extracted with CH_2Cl_2 (3 × 250 mL). The combined organics were washed with brine (250 mL), dried, filtered and concentrated *in vacuo* to give an ~50:50 mixture of **26** and **27**. Purification via flash column chromatography (eluent 30–40 °C petrol/Et_2O, 2:1 to 1:1) gave a mixture of **26** and **27** as a yellow oil (2.12 g, 82%, 50:50 dr); ^{1}H NMR (400 MHz, $CDCl_3$, selected peaks) 3.65 (s, 3H), 3.64 (s, 3H), 1.44 (s, 9H), 1.43 (s, 9H), 1.21 (s, 9H), 1.09 (s, 9H).

Step 2. Polymer supported $Pd(PPh_3)_4$ (550 mg, 0.06 mmol/g, 0.033 mmol) was added to a degassed solution of **26** and **27** (50:50 dr, 100 mg, 0.661 mmol) and DMBA (77 mg, 0.496 mmol) in CH_2Cl_2 (1.65 mL) under an argon atmosphere. The resultant mixture was stirred in the dark at rt for 18 h, then filtered through Celite® (eluent CH_2Cl_2). The filtrate concentrated *in vacuo*. Purification via flash column chromatography (eluent 30–40 °C petrol/acetone, 5:1 then 3:1) gave **28** as a colourless oil (31 mg, 35%). Further elution gave **40** as a white residue (40 mg, 46%); $[\alpha]_D^{25}$ −38.7 (*c* 1.0 in $CHCl_3$); ν_{max} 1707; ^{1}H NMR ($CDCl_3$, 500 MHz) 4.80 (dd, 1H, *J* 8.0, 2.9), 4.36–4.28 (m, 2H), 3.87 (ddd, 1H, *J* 11.9, 7.4, 4.5), 3.80–3.73 (m, 2H), 3.16–3.09 (m, 1H), 1.98–1.82 (m, 2H), 1.46 (s, 9H), 1.25 (s, 9H), 1.01 (br s, 21H); $^{13}C\{^1H\}$ NMR ($CDCl_3$, 125 MHz) 172.2, 153.4, 84.5, 75.7, 73.3, 66.7, 60.9, 59.9, 41.1, 34.9, 28.2, 22.7, 18.1, 18.0, 12.7; HRMS (ESI^+) $C_{25}H_{49}N_2O_6SSi^+$ ($[M+H]^+$) requires 533.3075; found 533.3073.

(1*R*,2*R*,7*R*,7a*S*)-1-Amino-2-(*tert*-butoxycarbonyl)oxy-7-hydroxypyrrolizin-3-one 41

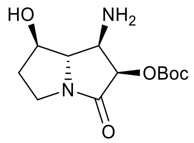

TBAF (1.0 M in THF, 0.88 mL, 0.88 mmol) was added to a solution of **40** (117 mg, 0.220 mmol) in THF (1.1 mL), and the resultant mixture was stirred at rt for 10 h. The resultant solution was concentrated *in vacuo*. Purification via flash column chromatography (eluent 30–40 °C petrol/acetone, 1:1 to 1:2) gave **41** as a white residue (31 mg, 52%); $[\alpha]_D^{25}$ +24.8 (*c* 1.0 in $CHCl_3$); ν_{max} 3289, 1676; ^1H NMR ($CDCl_3$, 400 MHz) 6.60 (d, 1H, *J* 2.8), 5.70–5.63 (m, 1H), 5.06 (d, 1H, *J* 1.4), 4.68 (dd, 1H, *J* 10.2, 2.9), 3.96 (dt, 1H, *J* 9.5, 7.3), 3.62–3.50 (m, 2H), 3.31–3.20 (m, 2H), 2.40–2.28 (m, 1H), 2.04–1.90 (m, 1H), 1.38 (s, 9H); $^{13}C\{^1H\}$ NMR ($CDCl_3$, 100 MHz) 171.7, 157.7, 81.0, 75.7, 74.8, 70.6, 62.1, 40.2, 32.3, 28.3; HRMS (ESI^+) $C_{12}H_{20}N_2NaO_5^+$ ($[M+H]^+$) requires 295.1264; found 295.1264.

3. X-ray Crystal Structures

X-Ray crystal structure determination for 16·$CHCl_3$ [CCDC 2212755]

Single crystals of **16**·$CHCl_3$ were obtained upon crystallisation via the slow diffusion method ($CHCl_3$/heptane, v:v 1:1). Data were collected using an Oxford Diffraction SuperNova diffractometer with graphite monochromated Cu-Kα radiation using standard procedures at 150 K. The structure was solved by direct methods (SIR92); all non-hydrogen atoms were refined with anisotropic thermal parameters. Hydrogen atoms were added at idealised positions. The structure was refined using CRYSTALS.

X-ray crystal structure data for **16**·$CHCl_3$ [$C_{15}H_{26}Cl_3NO_5$]: M = 406.74, monoclinic, space group $P\ 2_1$, a = 9.29029(11) Å, b = 10.42733(14) Å, c = 10.22998(14) Å, β = 91.7833(11)°, V = 990.53(2) Å^3, Z = 4, μ = 4.397 mm^{-1}, colourless block, crystal dimensions = 0.13 × 0.22 × 0.22 mm^3. A total of 4079 unique reflections were measured for 4 < θ < 76 and 3810 reflections were used in the refinement. The final parameters were wR_2 = 0.0570 and R_1 = 0.0224 [I>–3.0σ(I)], with Flack enantiopole = –0.009(8).

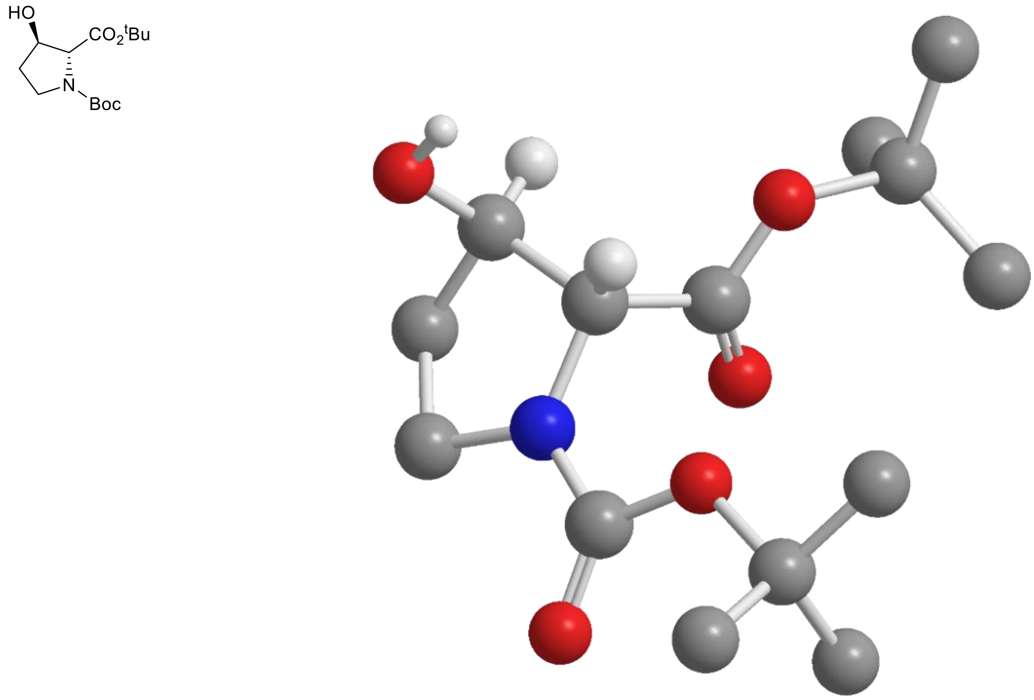

Figure S1. Simplified Chem3D representation of the X-ray crystal structure of **16**·$CHCl_3$ (selected H atoms and $CHCl_3$ molecule are omitted for clarity).

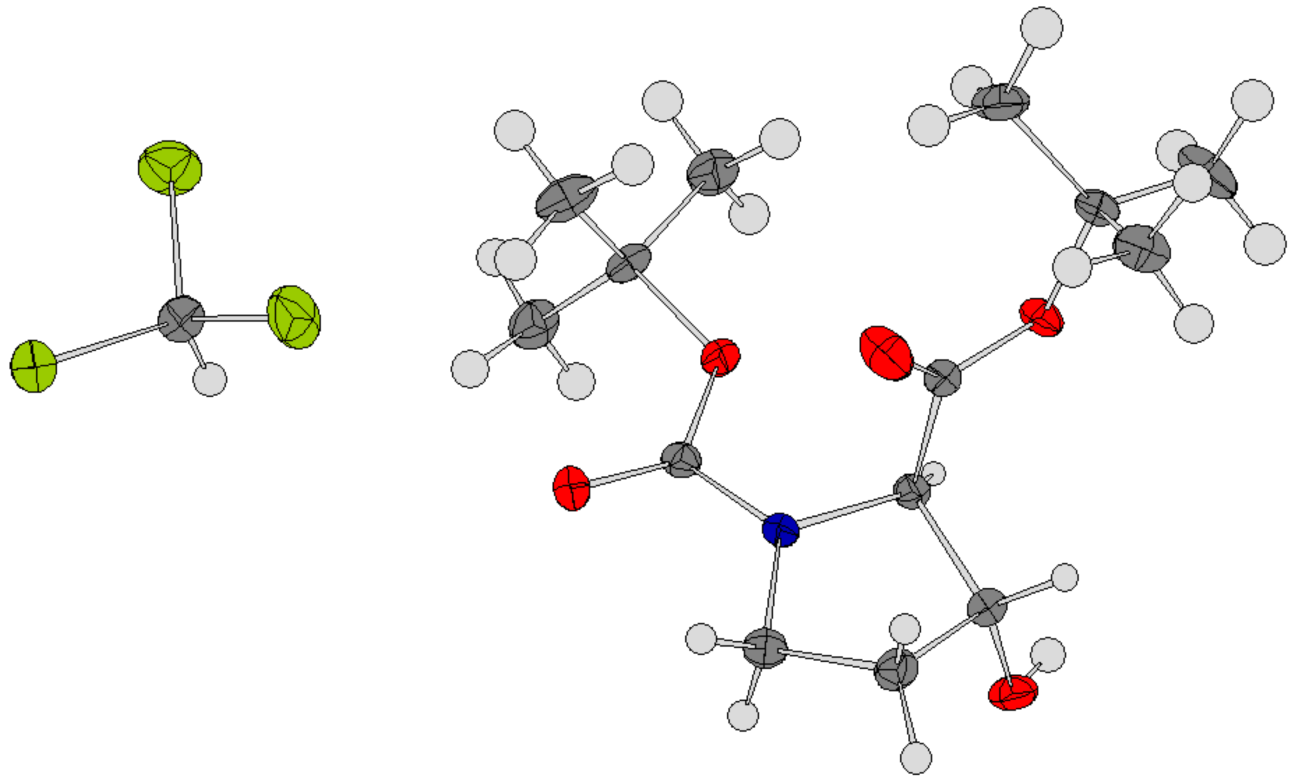

Figure S2. Ortep representation of the asymmetric unit of the X-ray crystal structure of **16**·$CHCl_3$ (ellipsoids shown at the 50% probability level).

X-Ray crystal structure determination for 29 [CCDC 2212756]

Single crystals of **29** were obtained upon crystallisation via the slow diffusion method ($CHCl_3$/heptane, v:v 1:1). Data were collected using an Oxford Diffraction SuperNova diffractometer with graphite monochromated Cu-Kα radiation using standard procedures at 150 K. The structure was solved by direct methods (SIR92); all non-hydrogen atoms were refined with anisotropic thermal parameters. Hydrogen atoms were added at idealised positions. The structure was refined using CRYSTALS.

X-ray crystal structure data for **29** [$C_{16}H_{28}N_2O_6Si$]: M = 376.47, orthorhombic, space group $P\ 2_1\ 2_1\ 2_1$, a = 5.96864(11) Å, b = 15.6792(3) Å, c = 20.5546(3) Å, V = 1923.57(6) Å^3, Z = 4, μ = 1.788 mm^{-1}, colourless block, crystal dimensions = 0.12 × 0.13 × 0.20 mm^3. A total of 3978 unique reflections were measured for $4 < \theta < 76$ and 3473 reflections were used in the refinement. The final parameters were wR_2 = 0.0812 and R_1 = 0.0347 [I>–3.0σ(I)], with Flack enantiopole = –0.011(17).

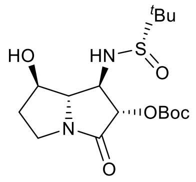

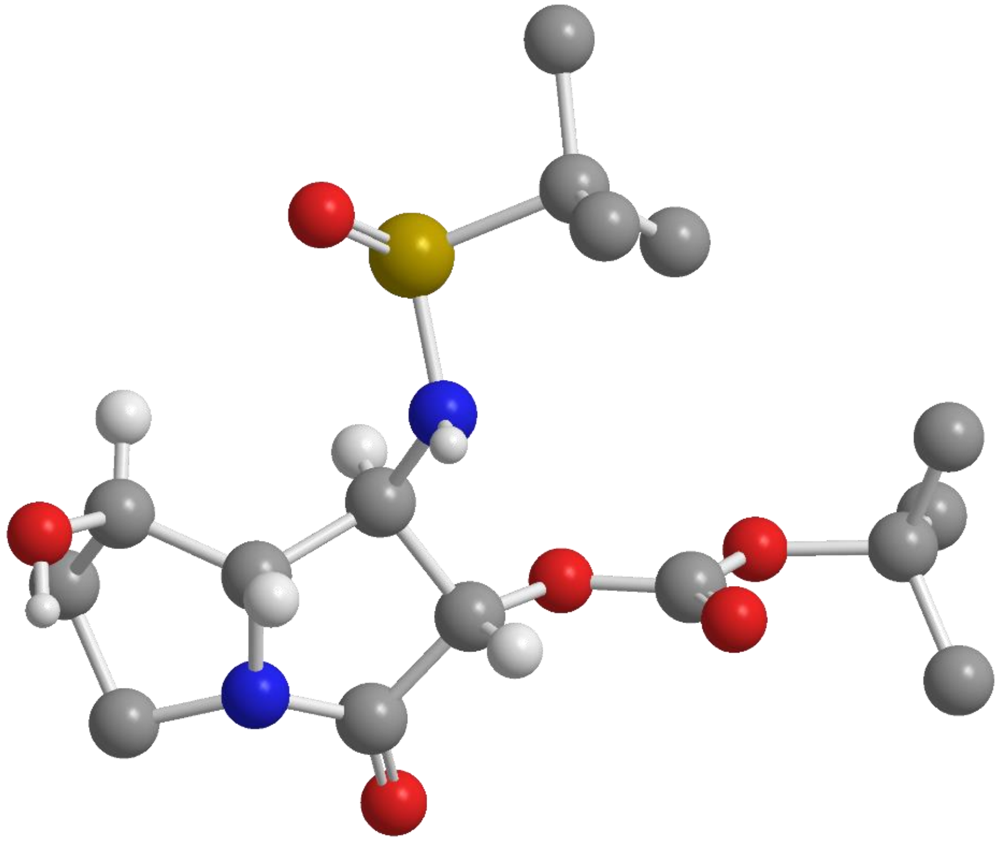

Figure S3. Simplified Chem3D representation of the X-ray crystal structure of **29** (selected H atoms are omitted for clarity).

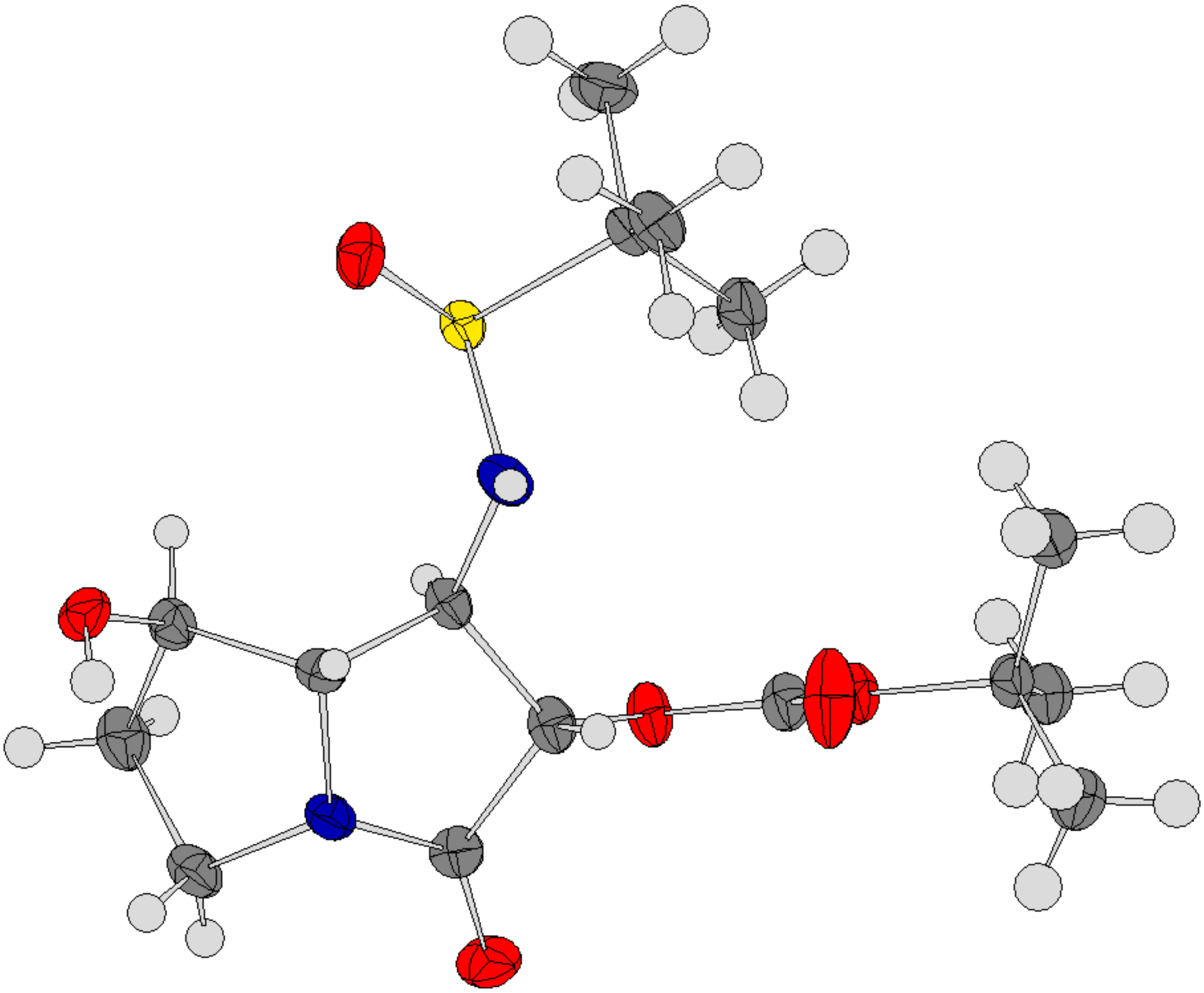

Figure S4. Ortep representation of the asymmetric unit of the X-ray crystal structure of **29** (ellipsoids shown at the 50% probability level).

X-ray crystal structure determination for 30·BH_3·H_2O [CCDC 2212757]

Single crystals of **30**·BH_3·H_2O were obtained upon crystallisation via the slow diffusion method ($CHCl_3$/heptane, v:v 1:1). Data were collected using an Oxford Diffraction SuperNova diffractometer with graphite monochromated Cu-Kα radiation using standard procedures at 150 K. The structure was solved by direct methods (SIR92); all non-hydrogen atoms were refined with anisotropic thermal parameters. Hydrogen atoms were added at idealised positions. The structure was refined using CRYSTALS.

X-ray crystal structure data for **30**·BH_3·H_2O [$C_{16}H_{35}N_2O_6Si$]: M = 394.34, orthorhombic, space group $P\ 2_1\ 2_1\ 2_1$, a = 5.99365(14) Å, b = 11.5309(2) Å, c = 30.9703(6) Å, V = 2140.43(8) Å^3, Z = 4, μ = 1.675 mm^{-1}, colourless prism, crystal dimensions = 0.06 × 0.09 × 0.19 mm^3. A total of 4497 unique reflections were measured for 3 < θ < 7 and 3791 reflections were used in the refinement. The final parameters were wR_2 = 0.1163 and R_1 = 0.0832 [I>–3.0σ(I)], with Flack enantiopole = –0.02(3).

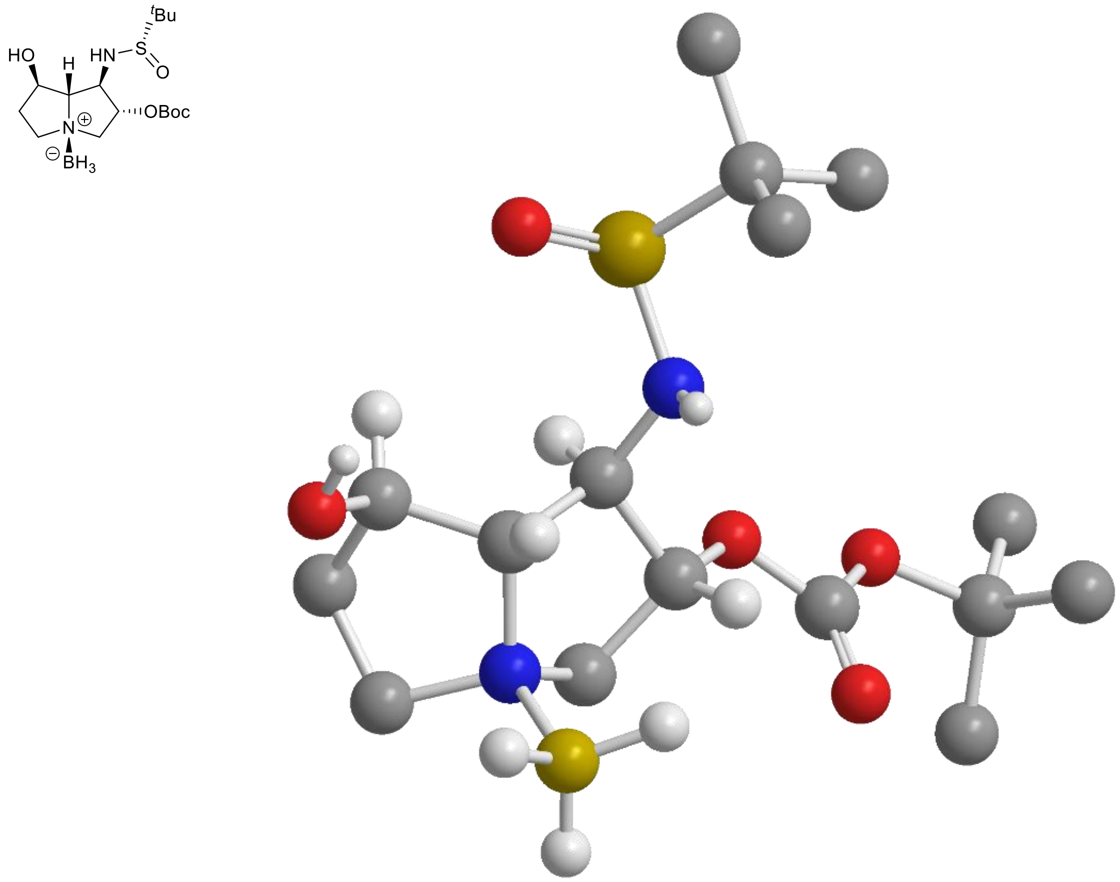

Figure S5. Simplified Chem3D representation of the X-ray crystal structure of **30**·BH_3·H_2O (selected H atoms and H_2O molecule are omitted for clarity).

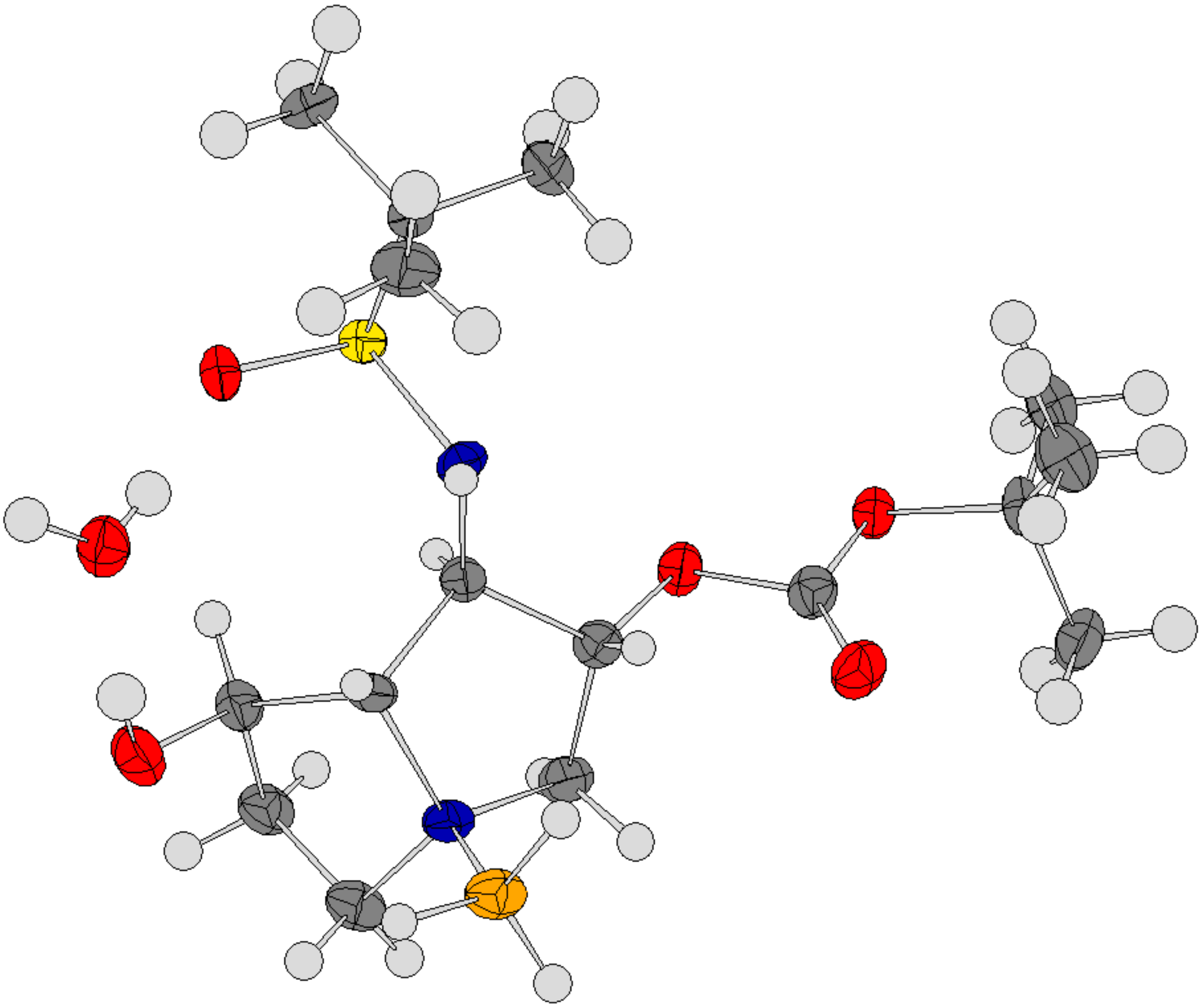

Figure S6. Ortep representation of the asymmetric unit of the X-ray crystal structure of **30**·BH_3·H_2O (ellipsoids shown at the 50% probability level).

X-ray crystal structure determination for 30·0.5H_2O [CCDC 2212758]

Single crystals of **30**·0.5H_2O were obtained upon crystallisation via the slow diffusion method ($CHCl_3$/heptane, v:v 1:1). Data were collected using an Oxford Diffraction SuperNova diffractometer with graphite monochromated Cu-Kα radiation using standard procedures at 150 K. The structure was solved by direct methods (SIR92); all non-hydrogen atoms were refined with anisotropic thermal parameters. Hydrogen atoms were added at idealised positions. The structure was refined using CRYSTALS.

X-ray crystal structure data for **30**·0.5H_2O [$C_{16}H_{31}N_2O_{0.5}S$]: M = 371.50, monoclinic, space group $P\ 2_1$, a = 6.14973(17) Å, b = 16.2619(3) Å, c = 20.3467(6) Å, β = 90.138(3)°, V = 2034.80(9) Å^3, Z = 4, μ = 1.660 mm^{-1}, colourless block, crystal dimensions = 0.17 × 0.20 × 0.31 mm^3. A total of 8251 unique reflections were measured for 2 < θ < 76 and 7765 reflections were used in the refinement. The final parameters were wR_2 = 0.1086 and R_1 = 0.0501 [I>–3.0σ(I)], with Flack enantiopole = –0.016(14).

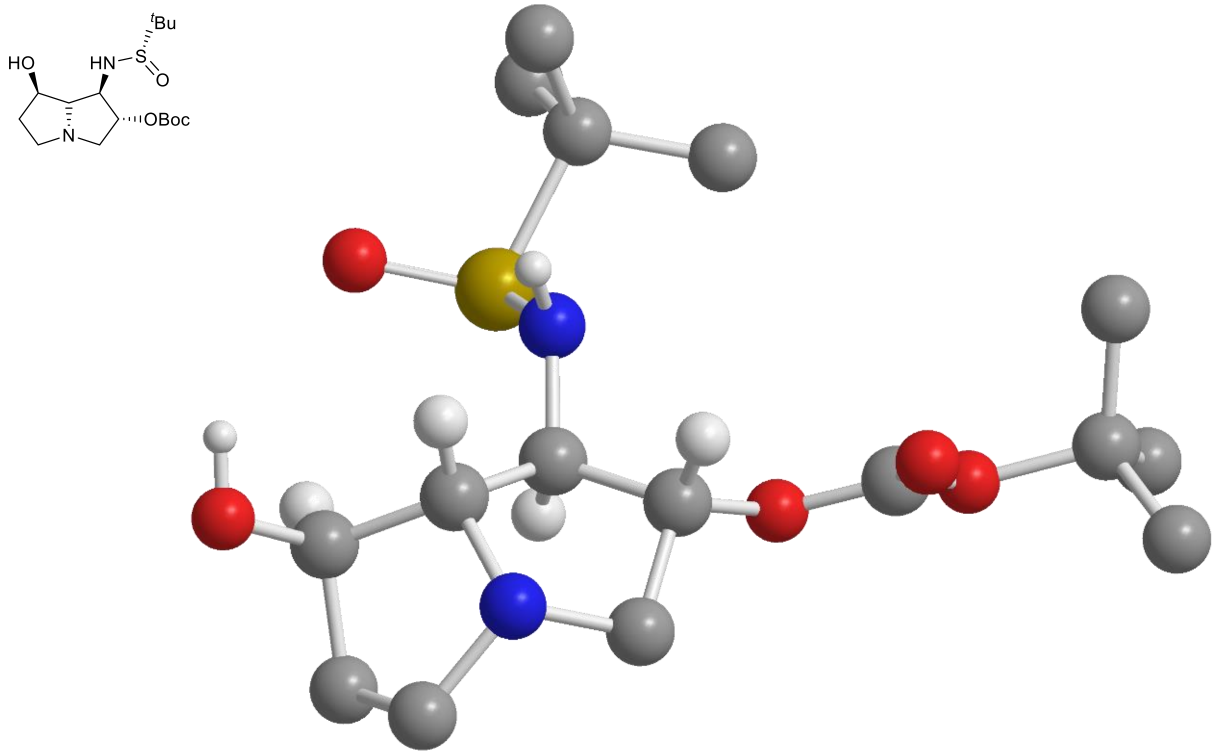

Figure S7. Simplified Chem3D representation of the X-ray crystal structure of **30**·0.5H_2O (selected H atoms and the H_2O molecule are omitted for clarity).

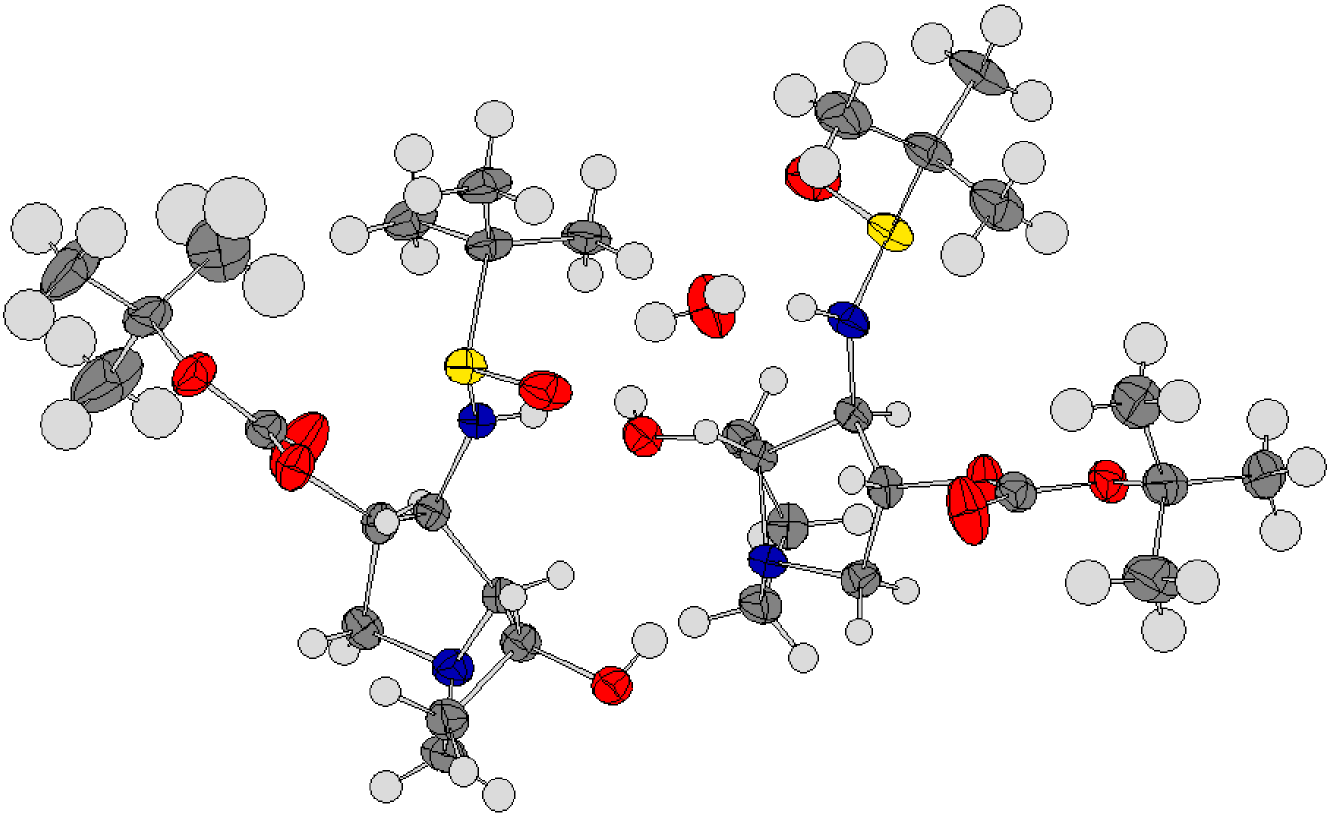

Figure S8. Ortep representation of the asymmetric unit of the X-ray crystal structure of **30**·0.5H_2O (ellipsoids shown at the 50% probability level).

X-ray crystal structure determination for 33·2HCl [CCDC 2212759]

Single crystals of **33**·2HCl were obtained upon crystallisation via the slow diffusion method (MeOH/heptane, v:v 1:1). Data were collected using an Oxford Diffraction SuperNova diffractometer with graphite monochromated Cu-Kα radiation using standard procedures at 150 K. The structure was solved by direct methods (SIR92); all non-hydrogen atoms were refined with anisotropic thermal parameters. Hydrogen atoms were added at idealised positions. The structure was refined using CRYSTALS.

X-ray crystal structure data for **33**·2HCl [$C_7H_{14}Cl_2N2O$]: M = 213.11, orthorhombic, space group $P\ 2_1\ 2_1\ 2_1$, a = 6.29038(10) Å, b = 6.50236(12) Å, c = 22.0613(3) Å, V = 902.36(3) Å^3, Z = 4, μ = 6.103 mm^{-1}, colourless prism, crystal dimensions = 0.10 × 0.12 × 0.27 mm^3. A total of 1901 unique reflections were measured for $4 < \theta < 77$ and 1690 reflections were used in the refinement. The final parameters were wR_2 = 0.0749 and R_1 = 0.0292 [$I > -3.0\sigma(I)$], with Flack enantiopole = –0.016(19).

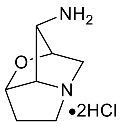

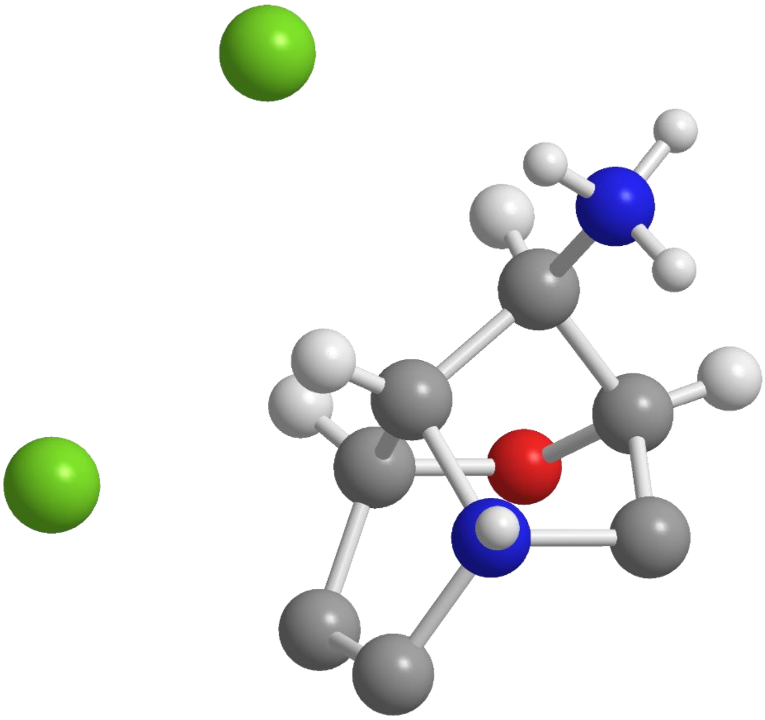

Figure S9. Simplified Chem3D representation of the X-ray crystal structure of **33**·2HCl (selected H atoms are omitted for clarity).

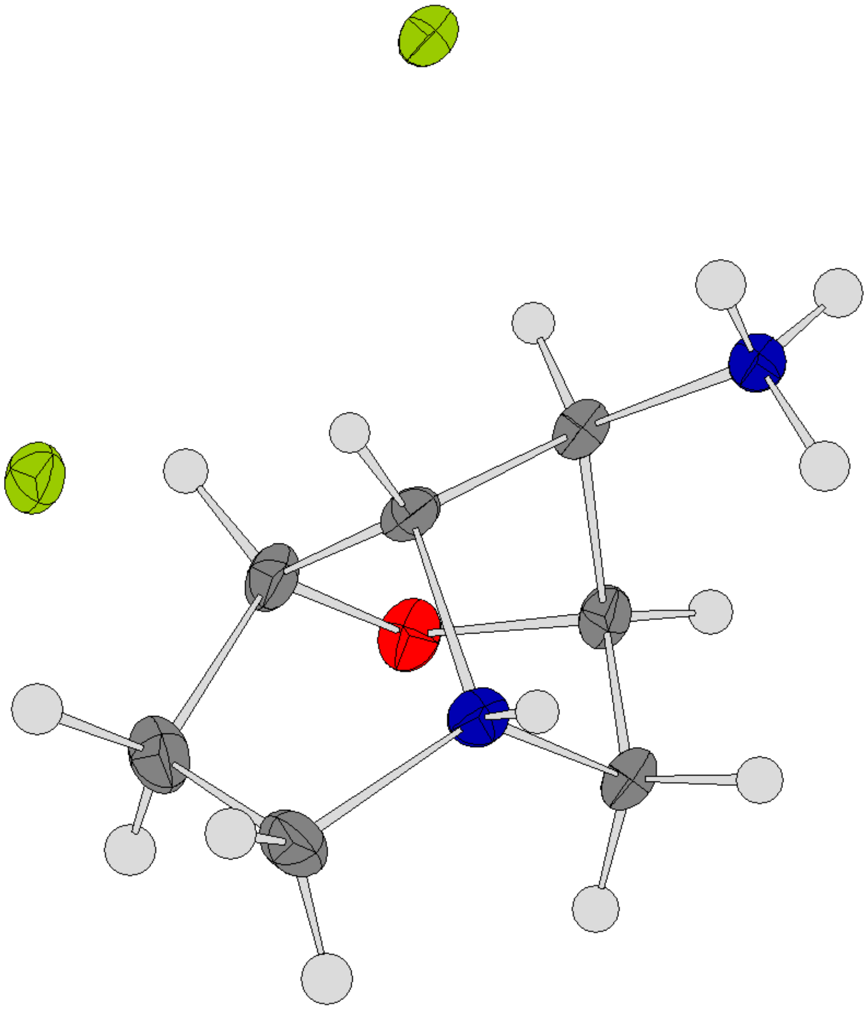

Figure S10. Ortep representation of the asymmetric unit of the X-ray crystal structure of **33**·2HCl (ellipsoids shown at the 50% probability level).

X-ray crystal structure determination for 39 [CCDC 2212760]

Single crystals of **39** were obtained upon crystallisation via the slow diffusion method ($CHCl_3$/heptane, v:v 1:1). Data were collected using an Oxford Diffraction SuperNova diffractometer with graphite monochromated Cu-Kα radiation using standard procedures at 150 K. The structure was solved by direct methods (SIR92); all non-hydrogen atoms were refined with anisotropic thermal parameters. Hydrogen atoms were added at idealised positions. The structure was refined using CRYSTALS.

X-ray crystal structure data for **39** [$C_{25}H_{48}N_2O_6SSi$]: M = 532.825, orthorhombic, space group $P\ 2_1\ 2_1\ 2_1$, a = 16.832(2) Å, b = 19.6714(13) Å, c = 56.6846(6) Å, V = 18602.9(4) Å^3, Z = 30, μ = 1.598 mm^{-1}, colourless prism, crystal dimensions = 0.16 × 0.18 × 0.27 mm^3. A total of 34696 unique reflections were measured for 3 < θ < 76 and 33354 reflections were used in the refinement. The final parameters were wR_2 = 0.1636 and R_1 = 0.075 [I>–3.0σ(I)], with Flack enantiopole = –007(16).

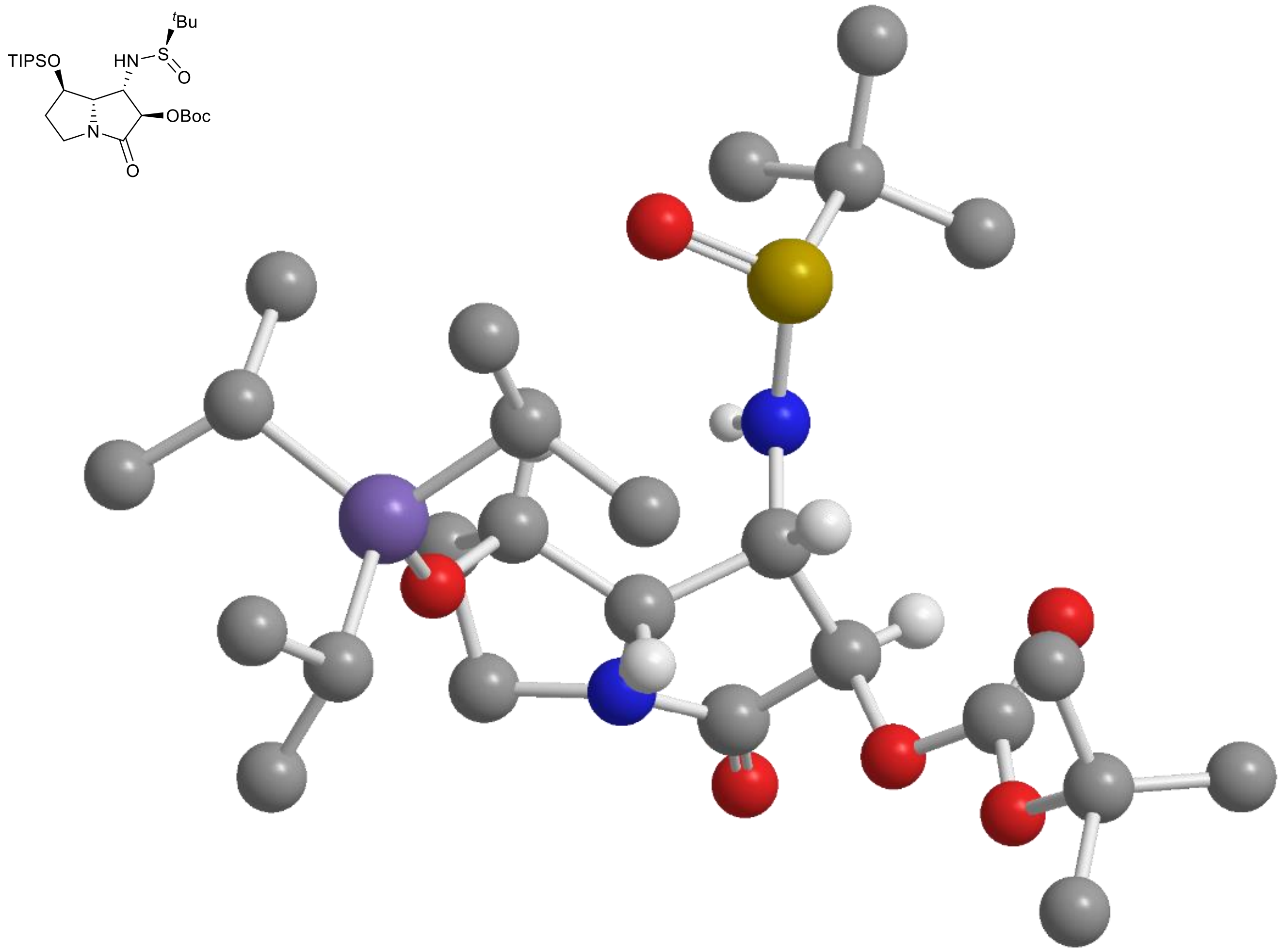

Figure S11. X-ray crystal structure of **39** (selected H atoms are omitted for clarity). Ellipsoids shown at 50% probability level.

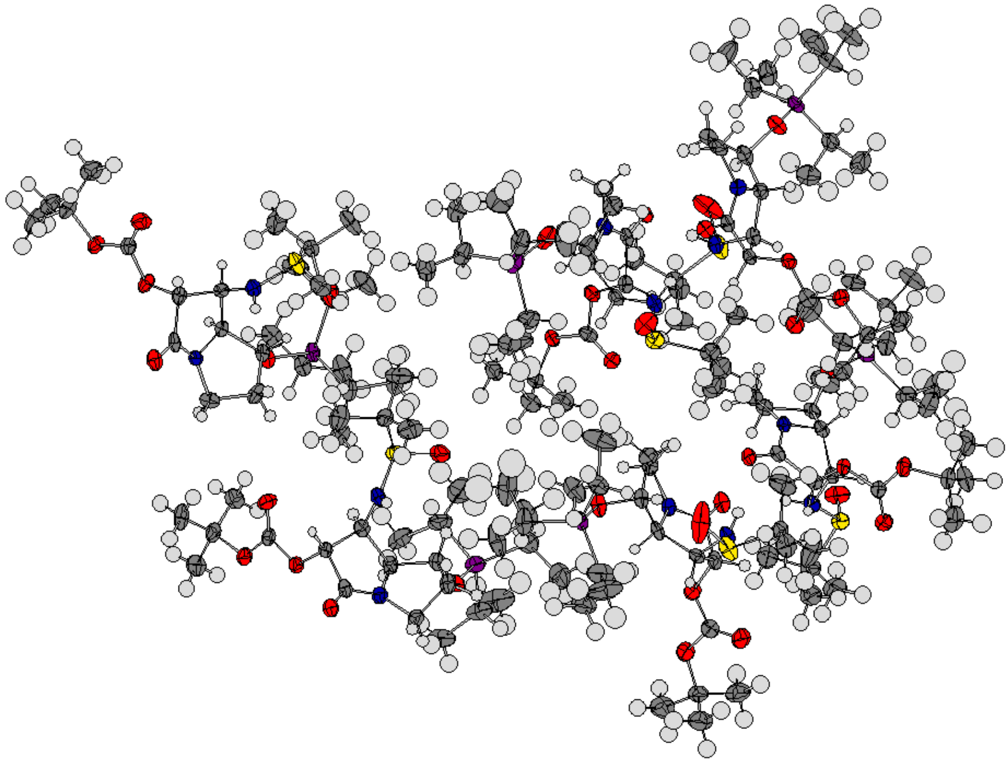

Figure S12. Ortep representation of the asymmetric unit of the X-ray crystal structure of **39** (ellipsoids shown at the 50% probability level).

4. Copies of ^{1}H and ^{13}C NMR Spectra

tert-Butyl (2*S*,3*S*,α*S*)-2-hydroxy-3-[*N*-benzyl-*N*-(α-methylbenzyl)amino]-5-benzyloxypentanoate 11

^{1}H NMR ($CDCl_3$, 400 MHz)

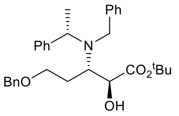

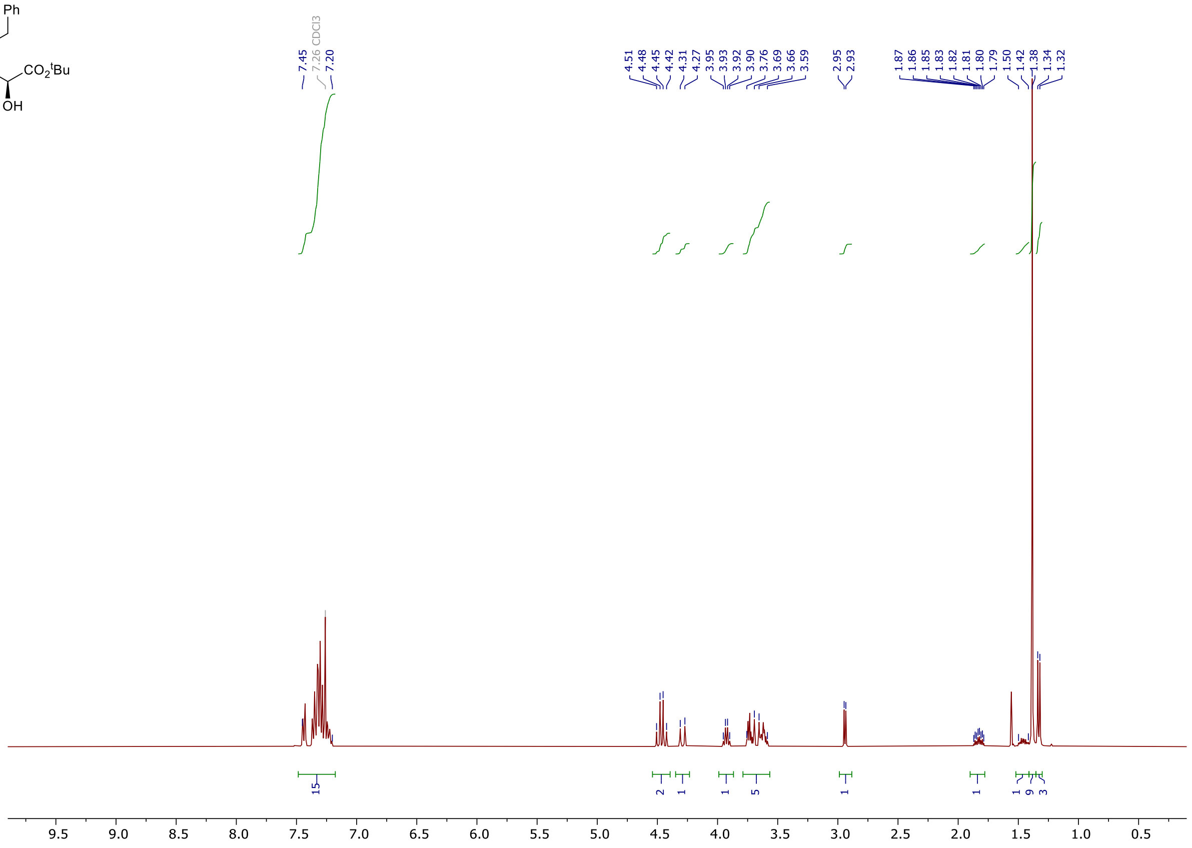

$^{13}C\{^{1}H\}$ NMR ($CDCl_3$, 100 MHz)

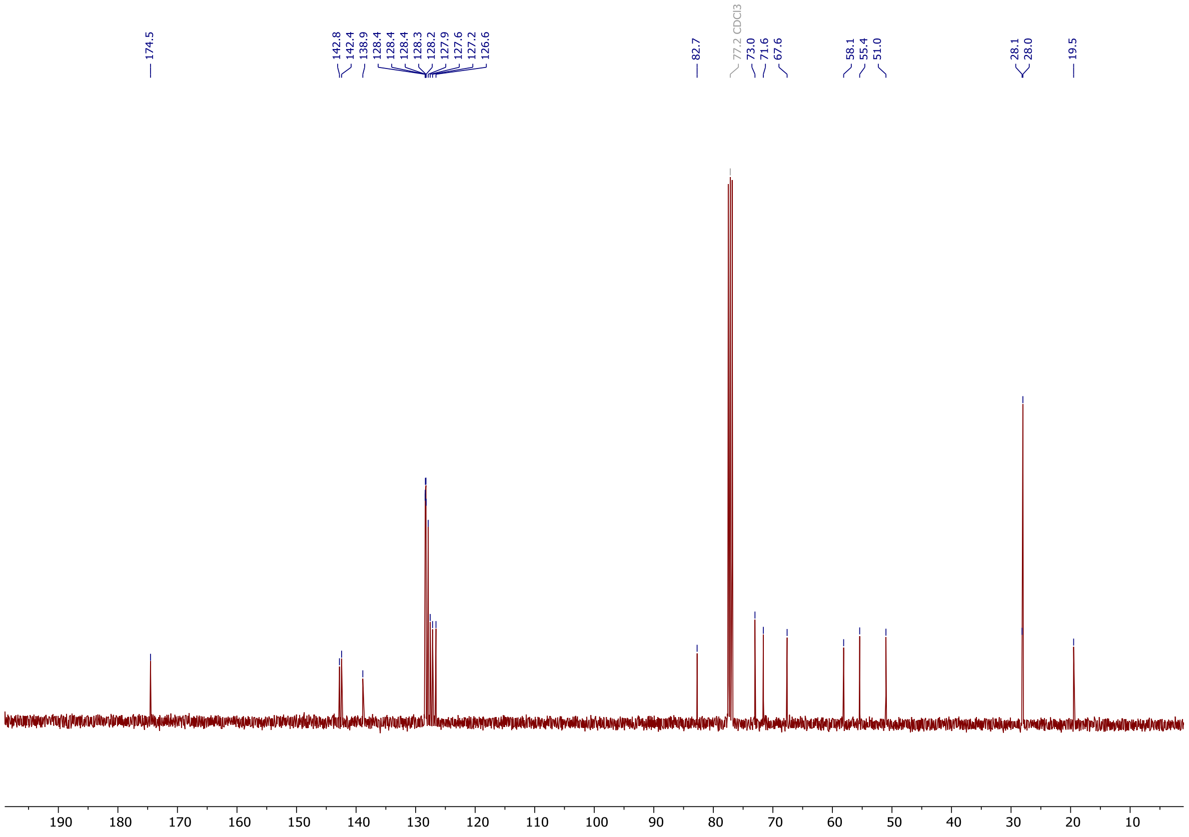

***tert*-Butyl (2*R*,3*R*,α*S*)-2-[*N*-benzyl-*N*-(α-methylbenzyl)]-3-hydroxy-5-benzyloxypentanoate 13**

^{1}H NMR ($CDCl_3$, 400 MHz)

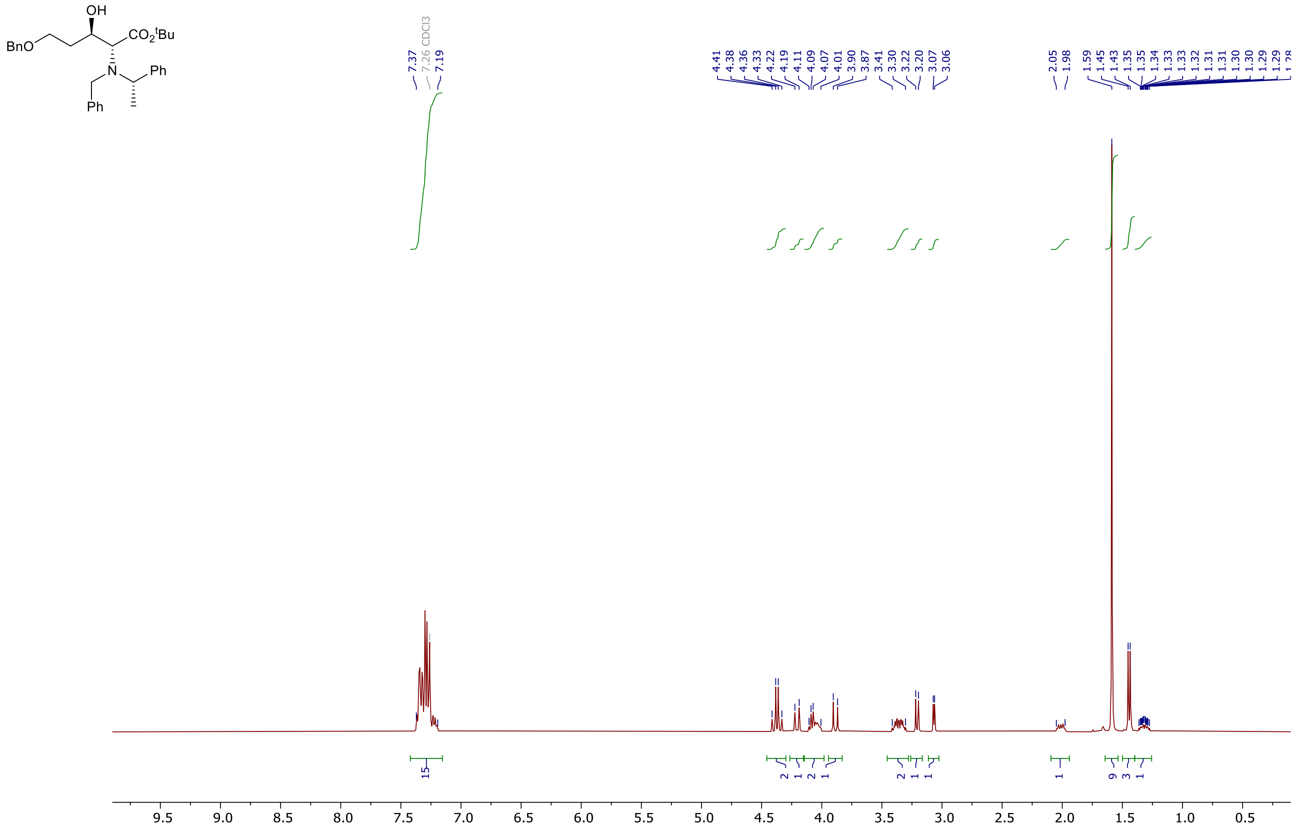

$^{13}C\{^{1}H\}$ NMR ($CDCl_3$, 100 MHz)

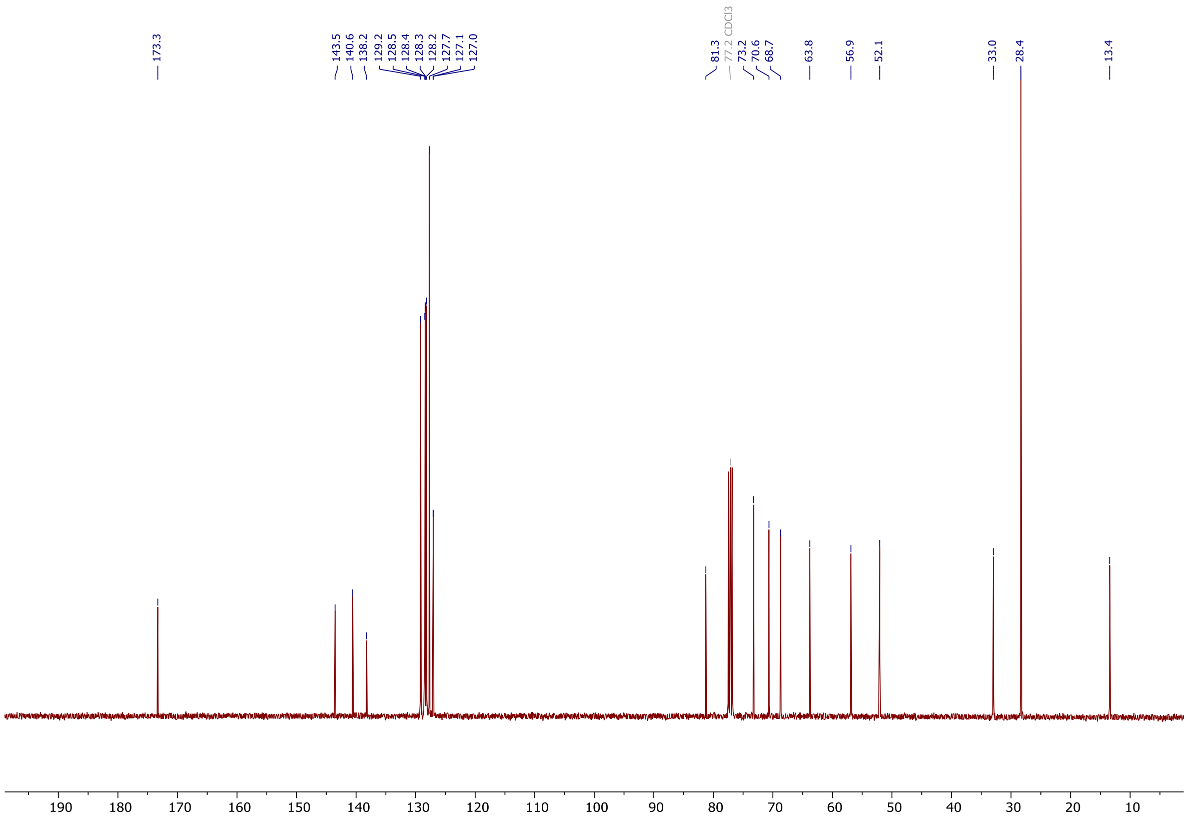

***tert*-Butyl (2*R*,3*R*)-2-[(*N*-*tert*-butoxycarbonyl)amino]-3,5-dihydroxypentanoate 14**

^{1}H NMR (400 MHz, MeOH-*d*$_4$)

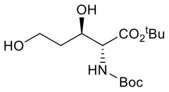

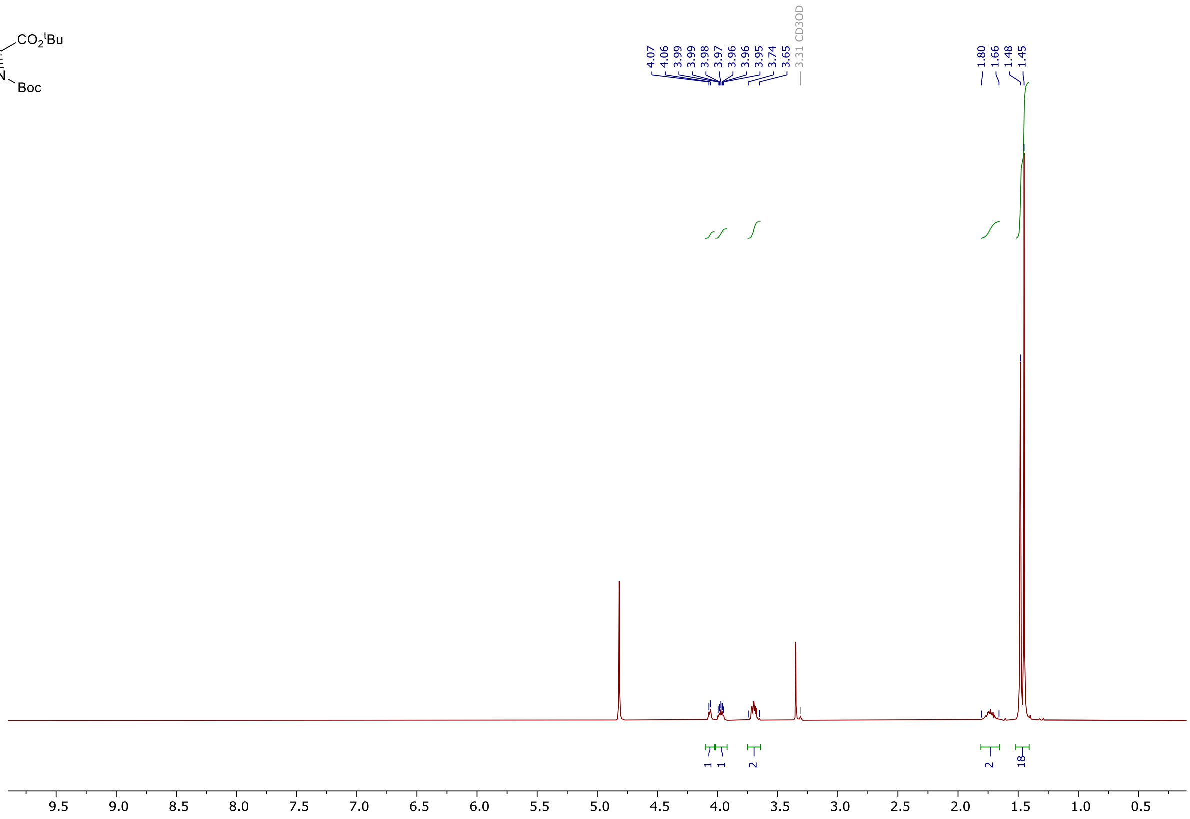

^{13}C{^{1}H} NMR (100 MHz, MeOH-*d*$_4$)

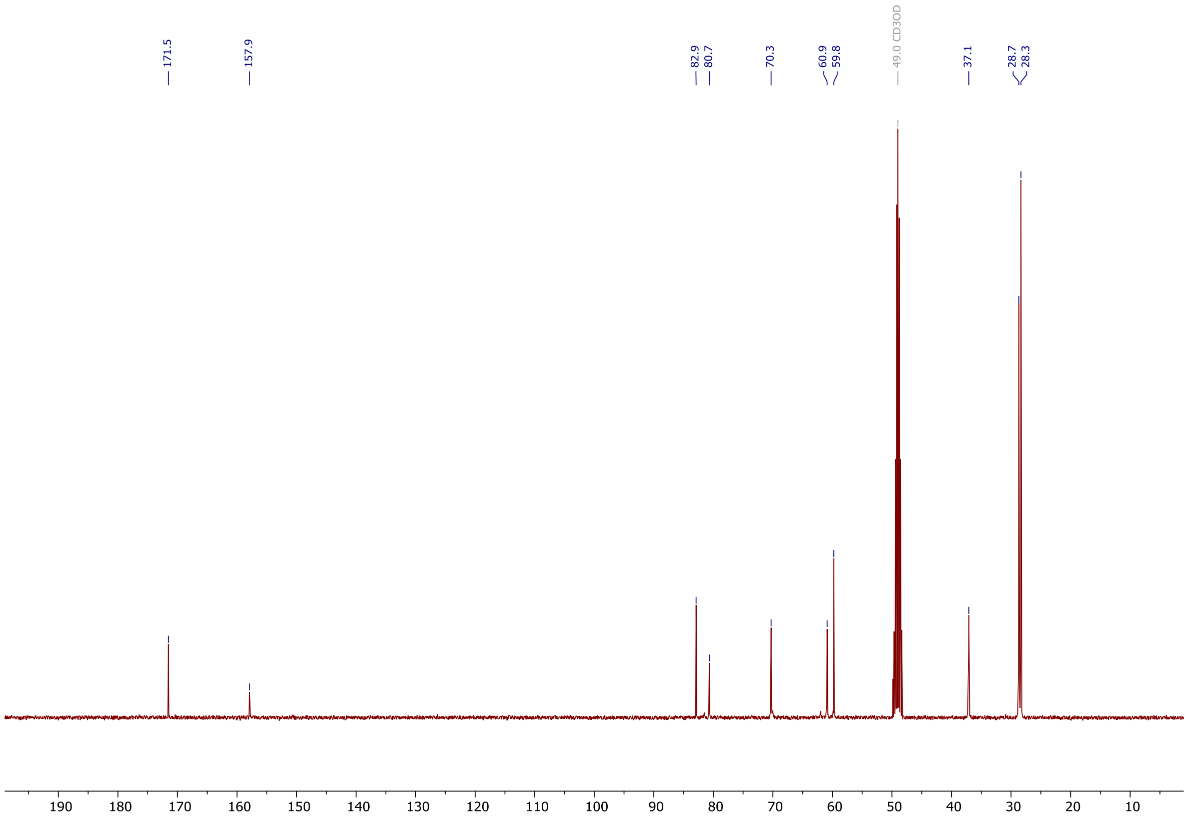

***tert*-Butyl (2*R*,3*R*)-2-(*N*-*tert*-butoxycarbonylamino)-3-hydroxy-5-(naphthalen-2′-ylsulfonyloxy)pentanoate 15**

^{1}H NMR ($CDCl_3$, 400 MHz)

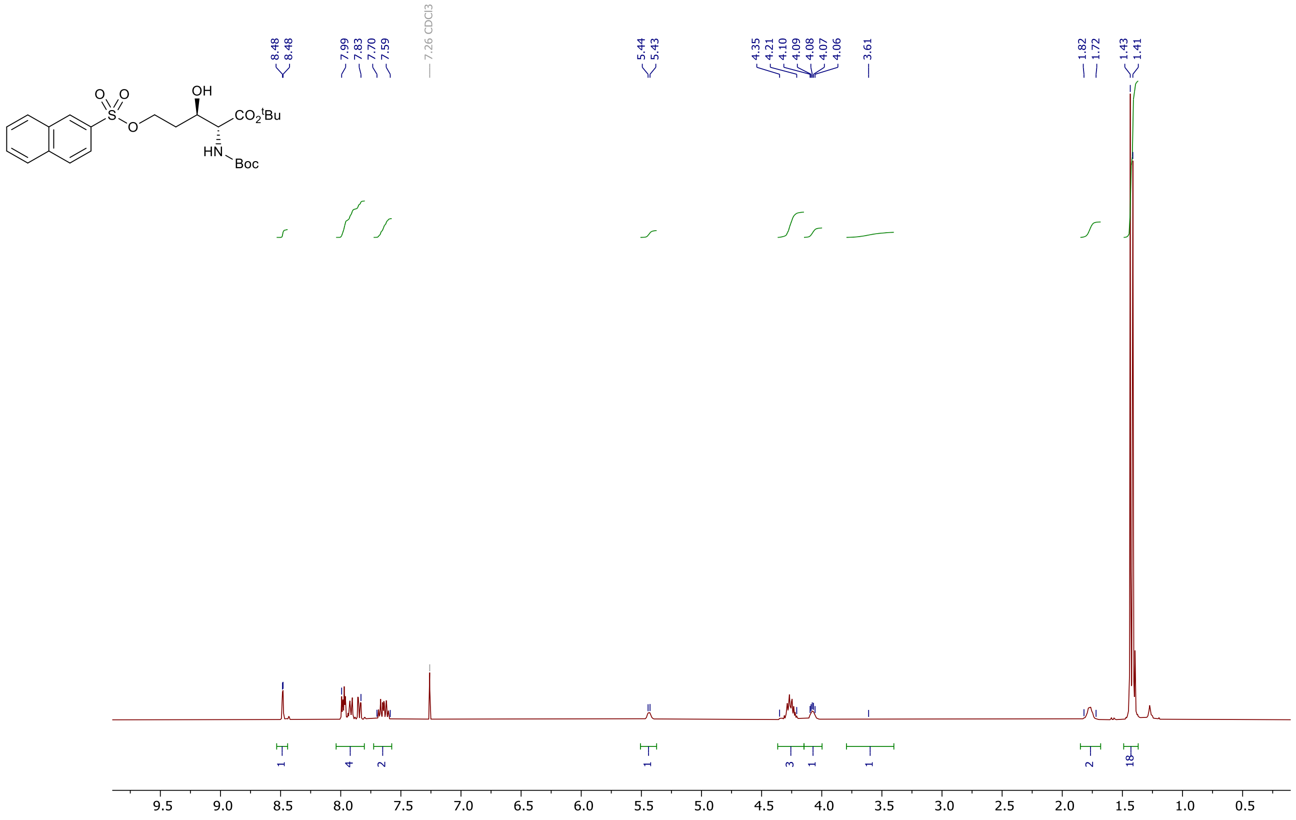

^{13}C{^{1}H} NMR ($CDCl_3$, 100 MHz)

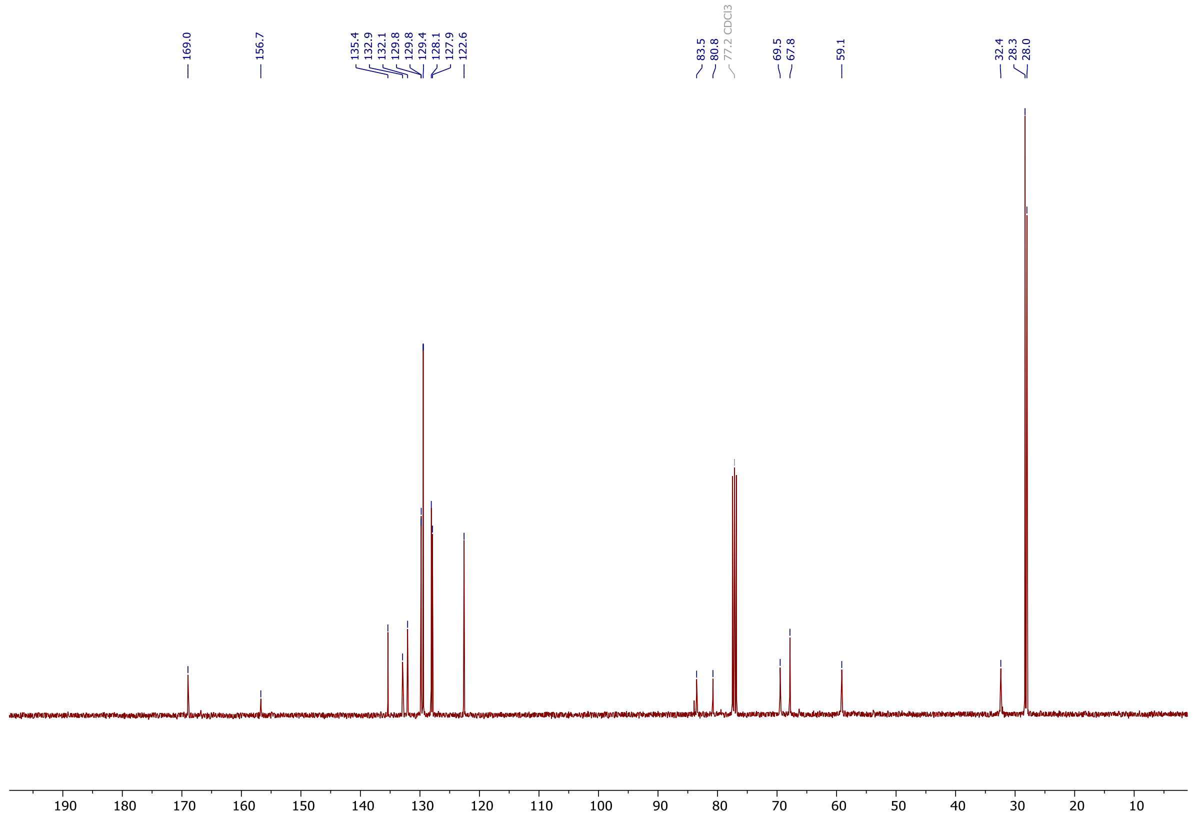

(2*R*,3*R*)-1,2-Bis-*tert*-butoxycabronyl-3-pyrrolidinol 16

^{1}H NMR (PhMe-d_8, 363 K, 500 MHz)

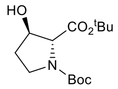

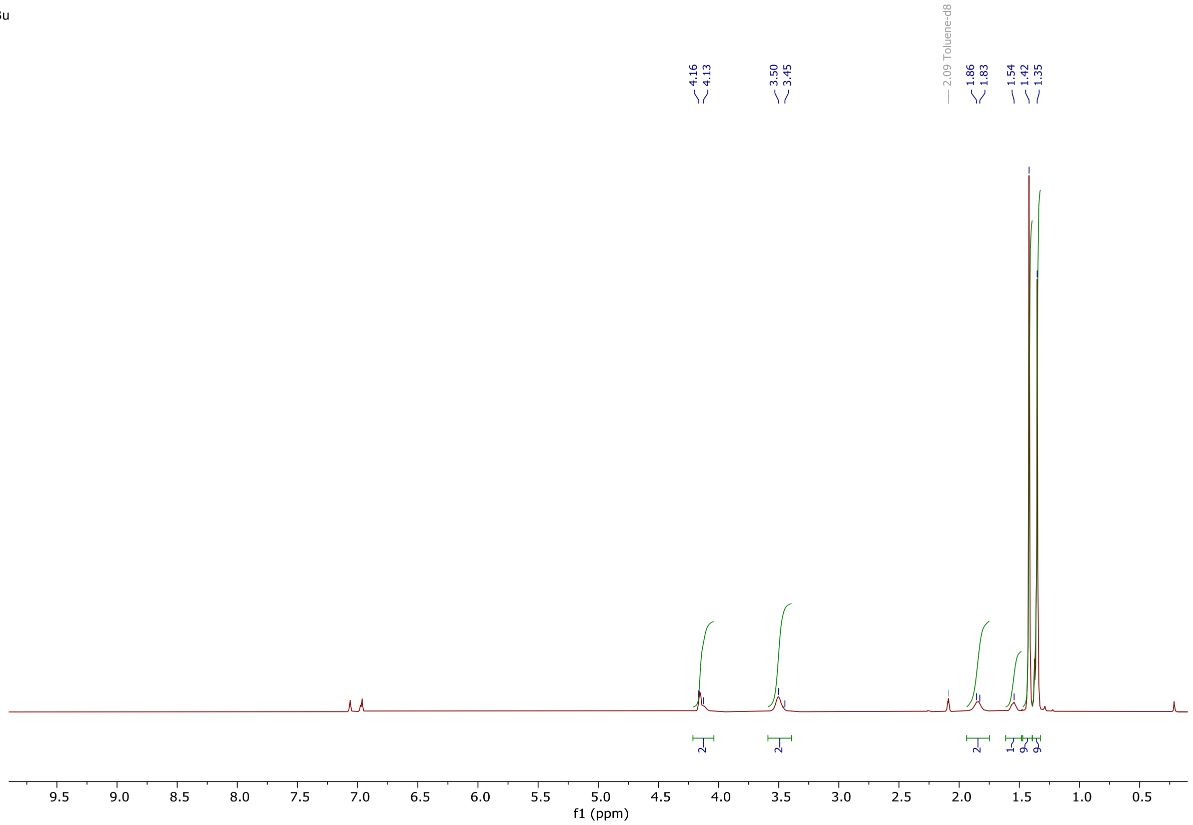

$^{13}C\{^{1}H\}$ NMR (PhMe-d_8, 363 K, 125 MHz)

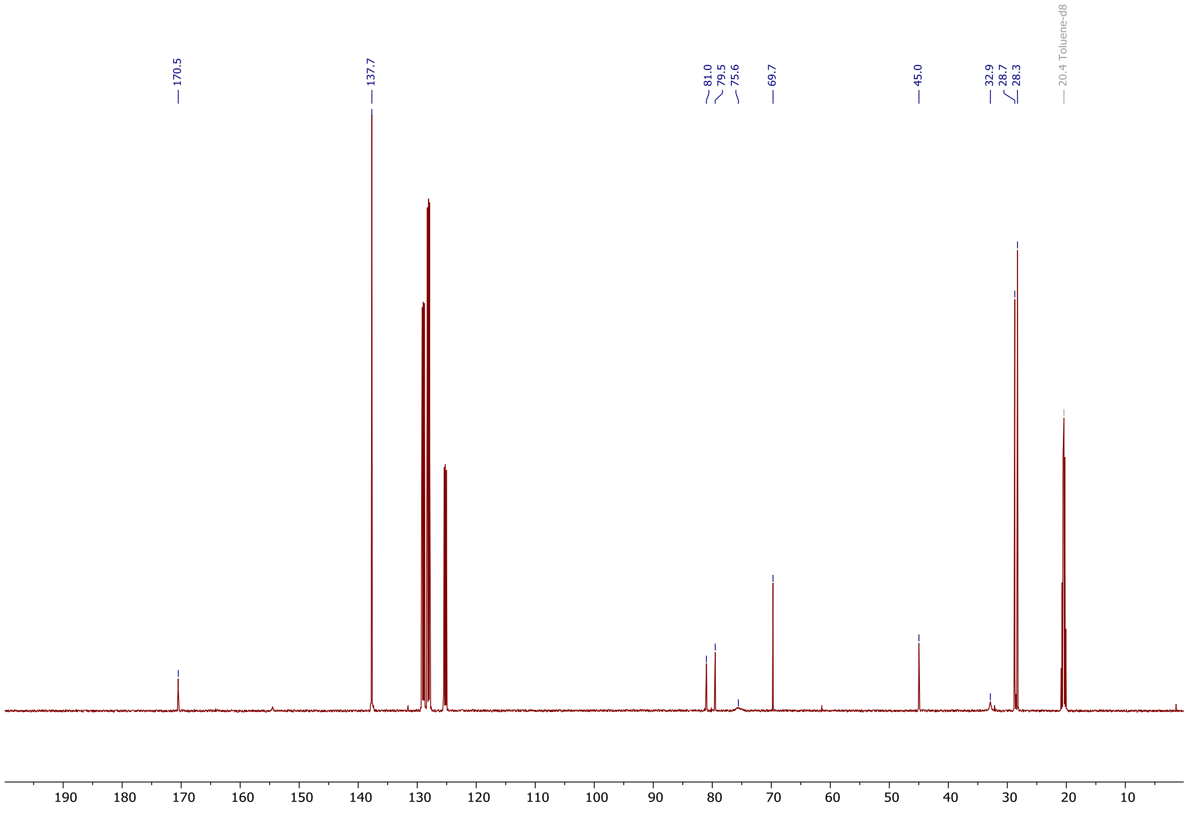

(2*R*,3*R*)-1,2-Bis-*tert*-butoxycabronyl-3-triisopropylsilyloxypyrrolidine 17

^{1}H NMR (400 MHz, CDCl$_3$, rotameric)

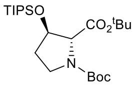

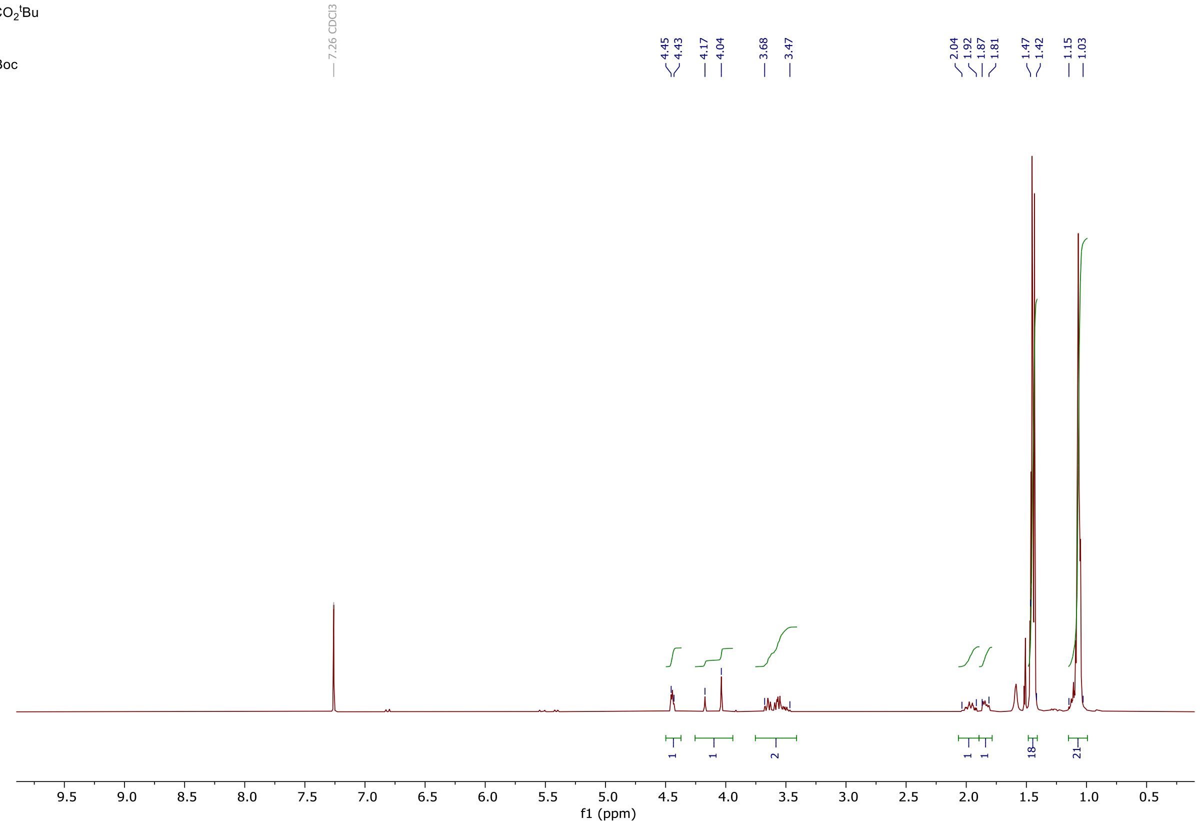

^{13}C{^{1}H} NMR (100 MHz, CDCl$_3$, rotameric)

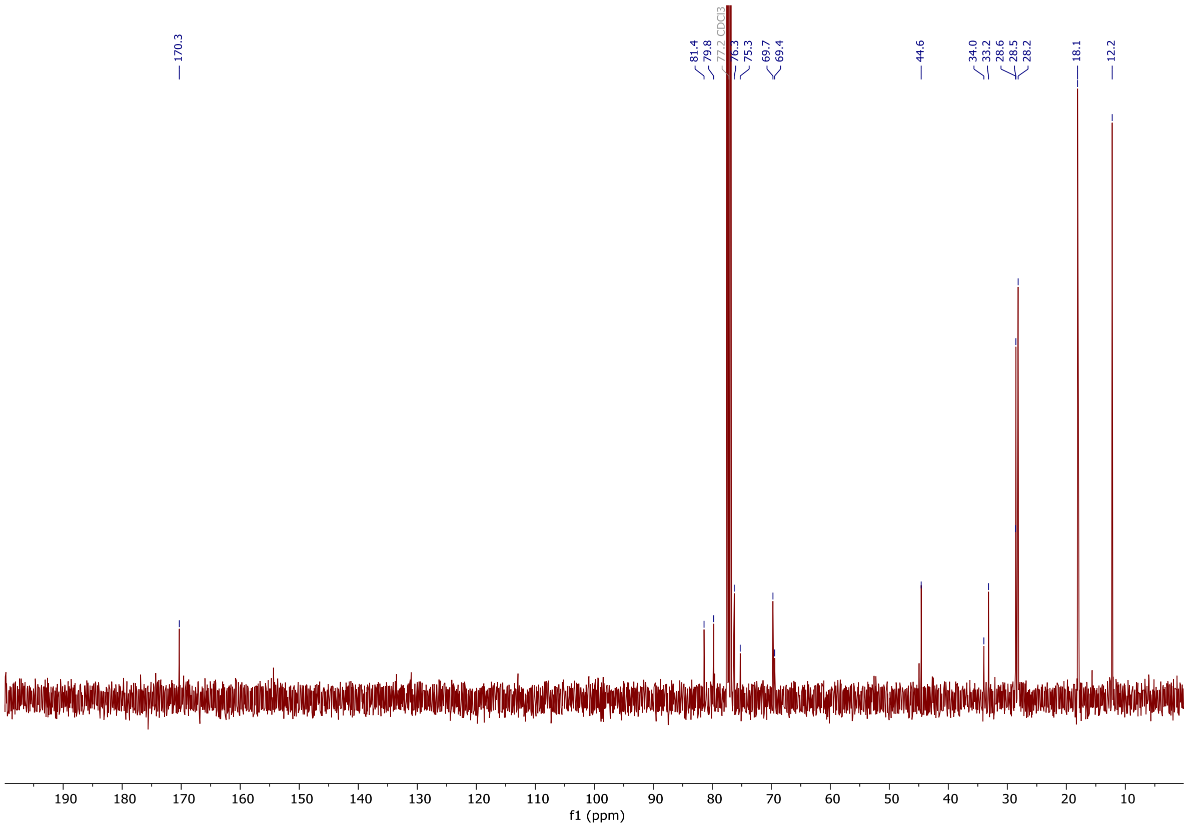

(2*R*,3*R*)-1-Allyl-2-*tert*-butoxycarbonyl-3-triisopropylsilyloxypyrrolidine 18

^{1}H NMR ($CDCl_3$, 400 MHz)

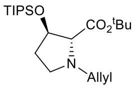

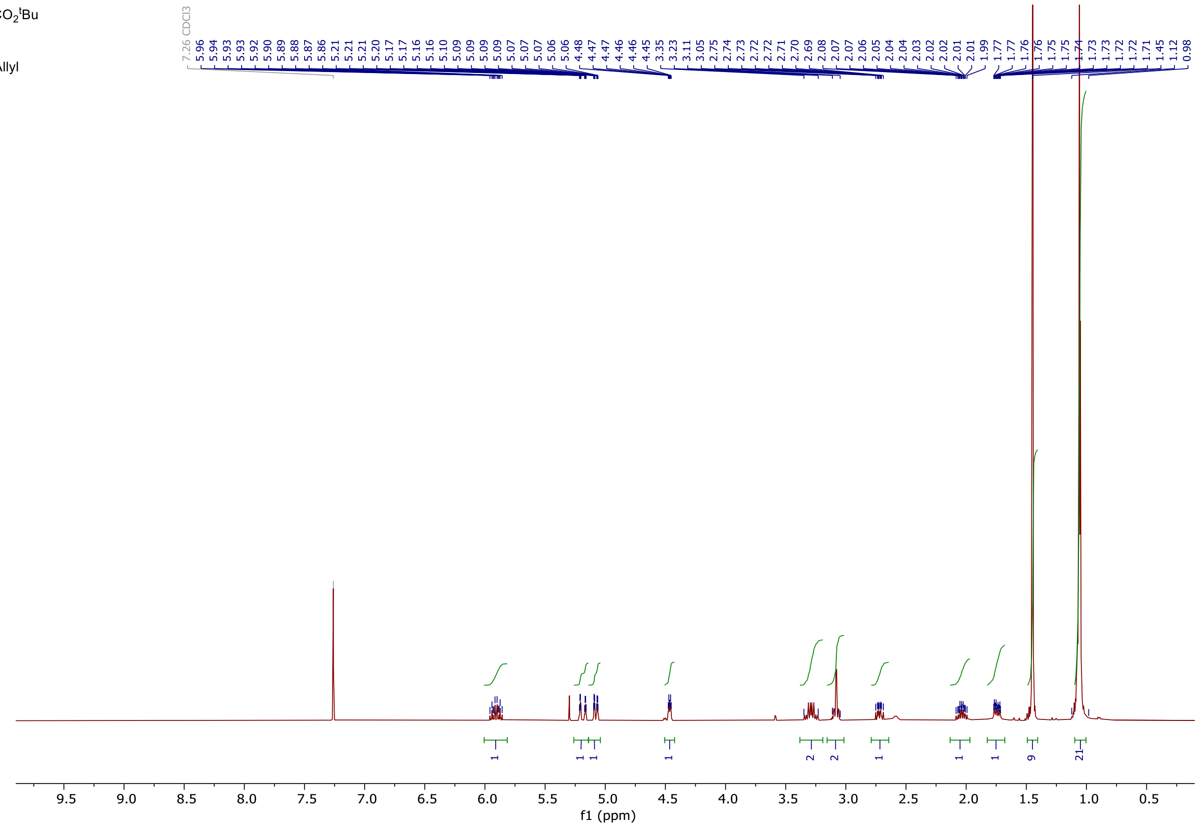

(2*S*,3*R*,*R*S,*E*)-*N*-[(1-allyl-3-triisopropylsilyloxypyrrolidin-2-yl)methylene]-*tert*-butylsulfinamide (*R*S)-20

^{1}H NMR ($CDCl_3$, 400 MHz)

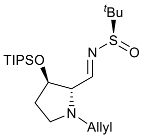

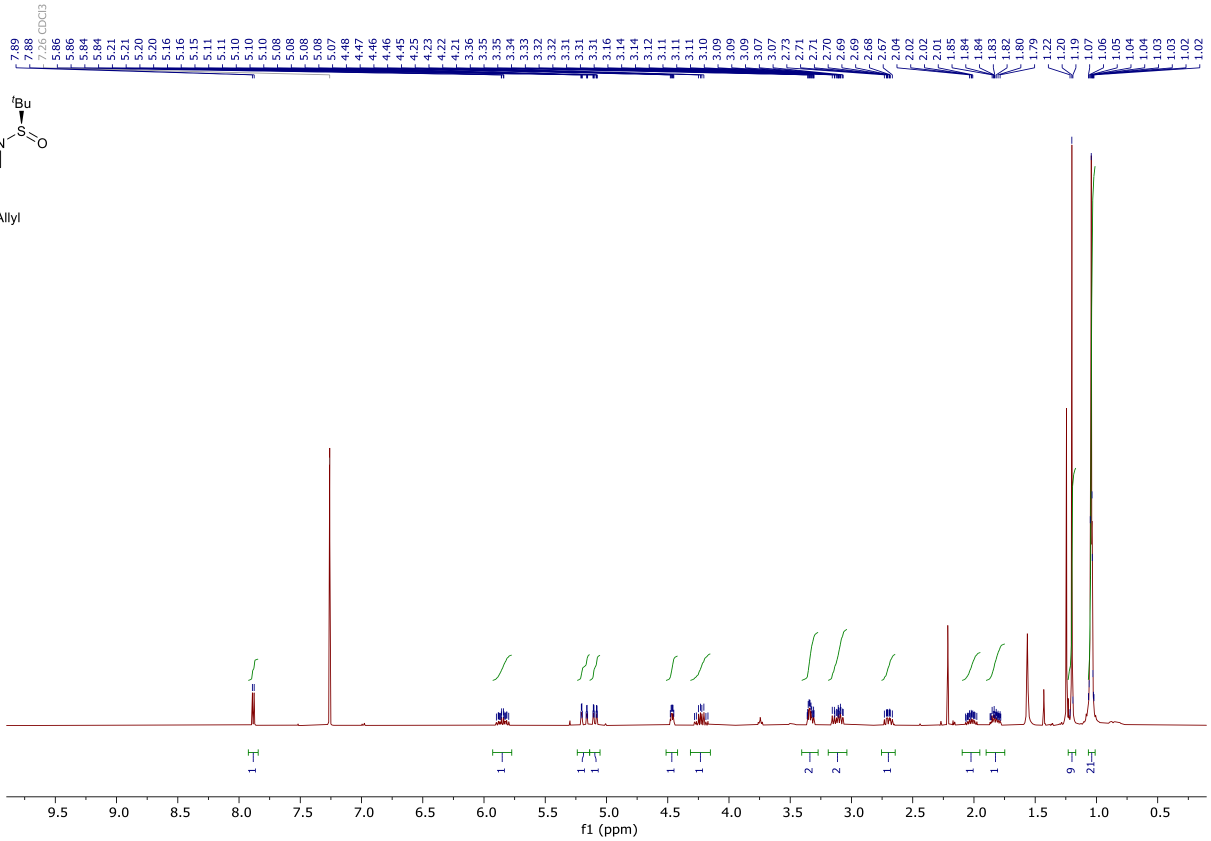

(2*S*,3*R*,*S*S,*E*)-*N*-[(1-allyl-3-triisopropylsilyloxypyrrolidin-2-yl)methylene]-*tert*-butylsulfinamide (*S*S)-21

^{1}H NMR ($CDCl_3$, 400 MHz)

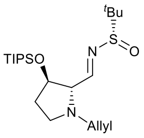

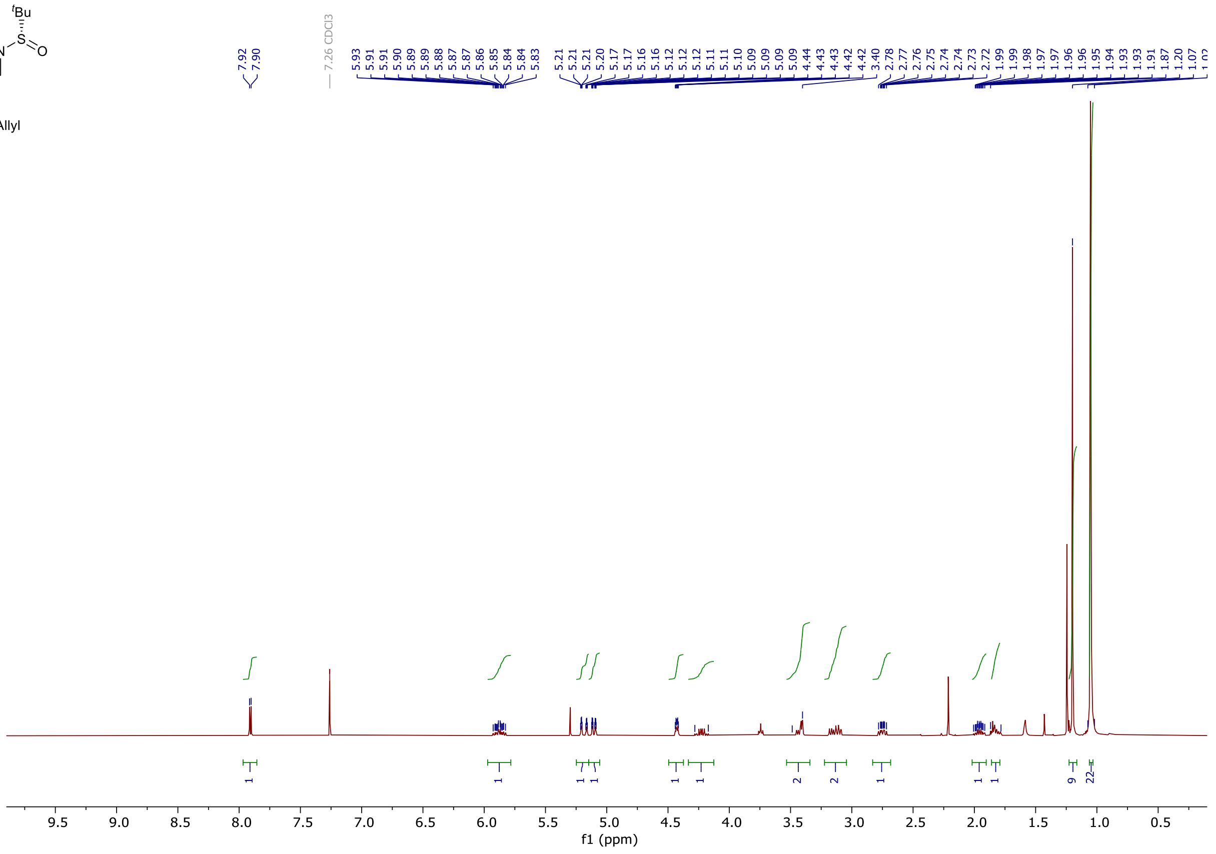

O-tert-Butoxycarbonyl methyl glycolate 22

^{1}H NMR ($CDCl_3$, 400 MHz)

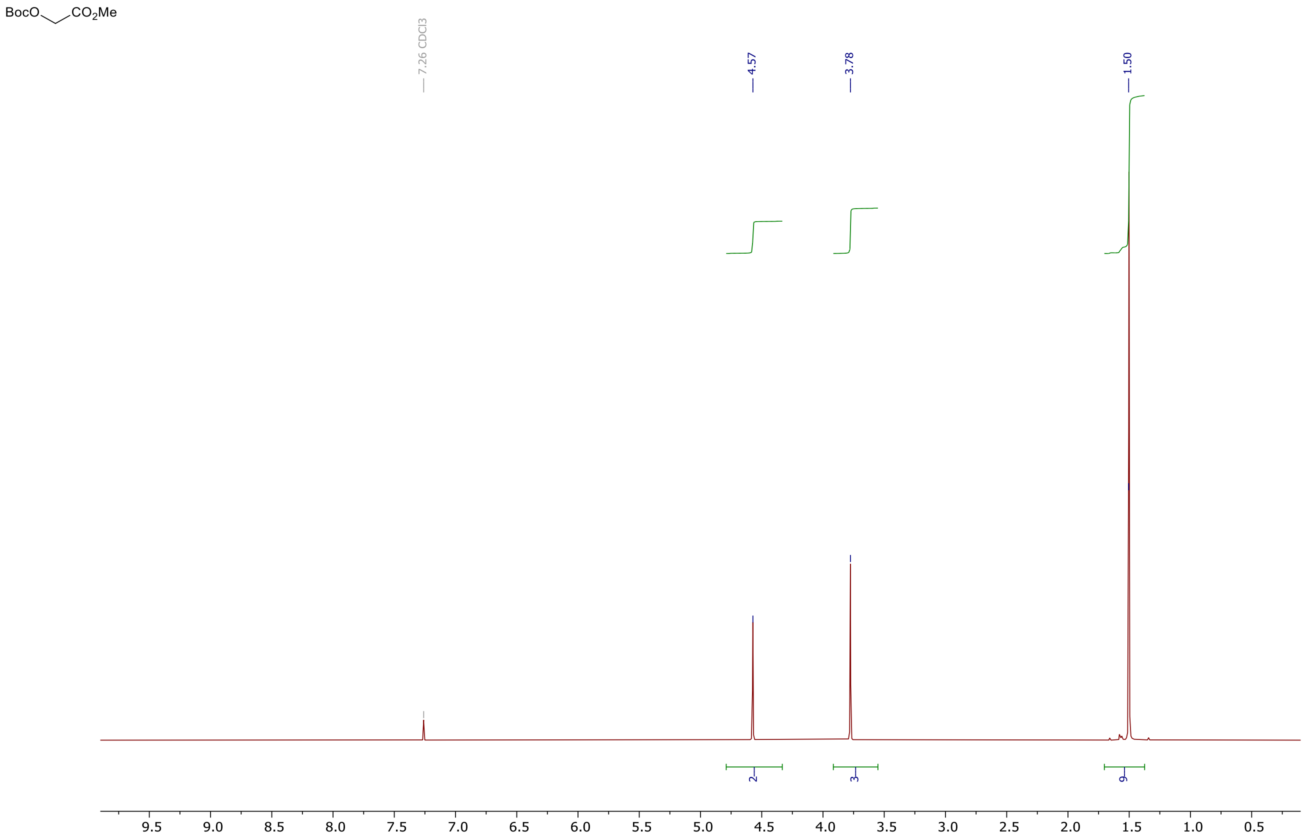

Methyl (2*R*,3*S*,2′*S*,3′*R*,*R*s)-2-(*tert*-butoxycarbonyl)oxy-3-(*tert*-butylsulfinyl)amino-3-(1′-allyl-3′-triisopropylsilyloxypyrrolidin-2′-yl)propanoate 25

^{1}H NMR ($CDCl_3$, 400 MHz)

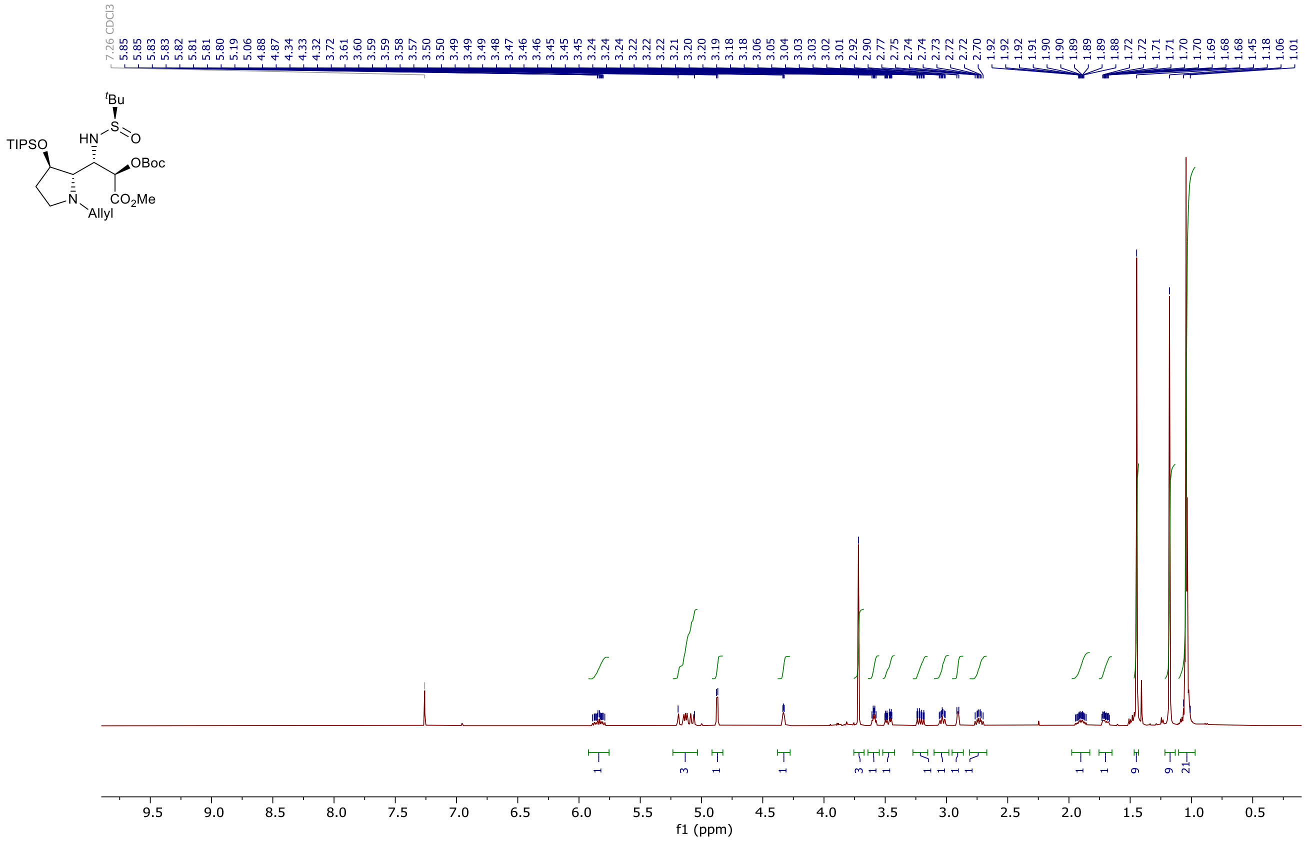

$^{13}C\{^{1}H\}$ NMR ($CDCl_3$, 100 MHz)

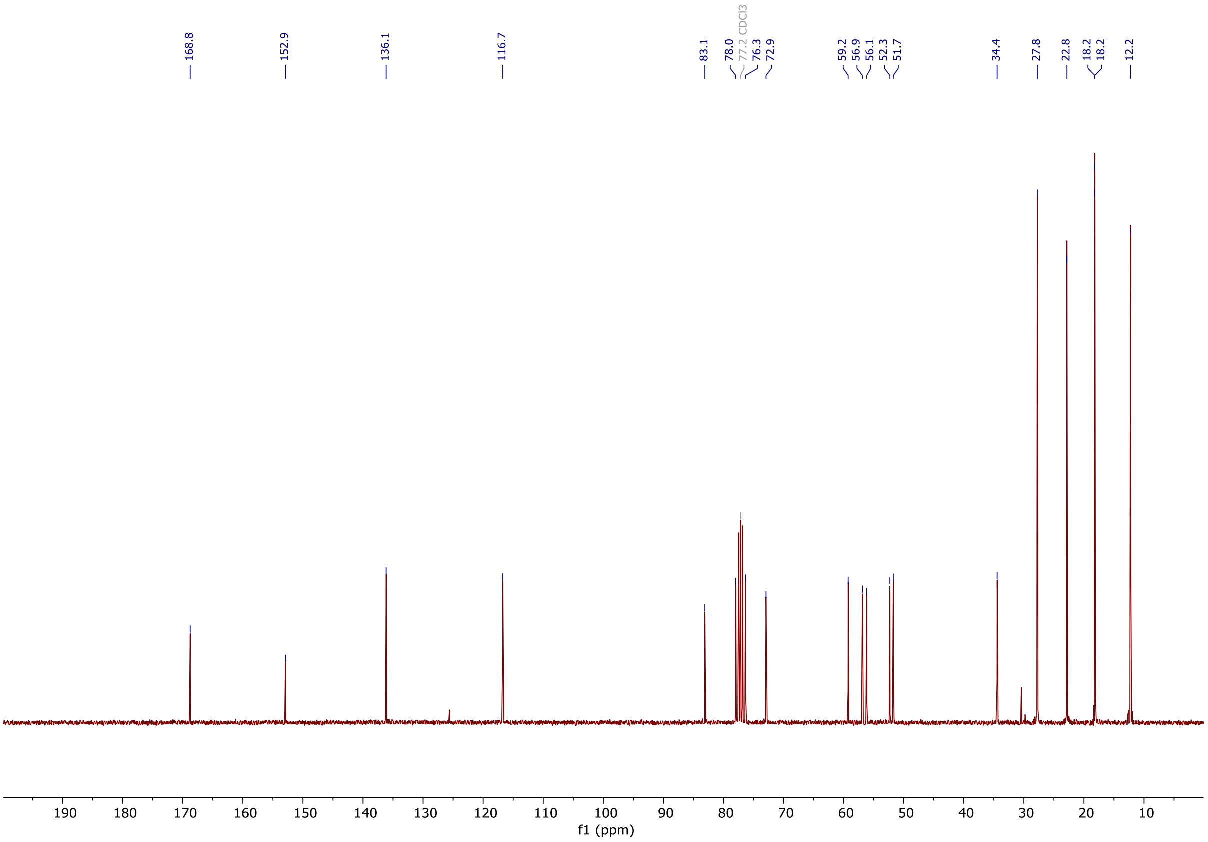

Methyl (2*S*,3*R*,2′*S*,3′*R*,*S*S)-2-(*tert*-butoxycarbonyl)oxy-3-(*tert*-butylsulfinyl)amino-3-(1′-allyl-3′-triisopropylsilyloxypyrrolidin-2′-yl)propanoate 26

^{1}H NMR ($CDCl_3$, 400 MHz)

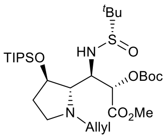

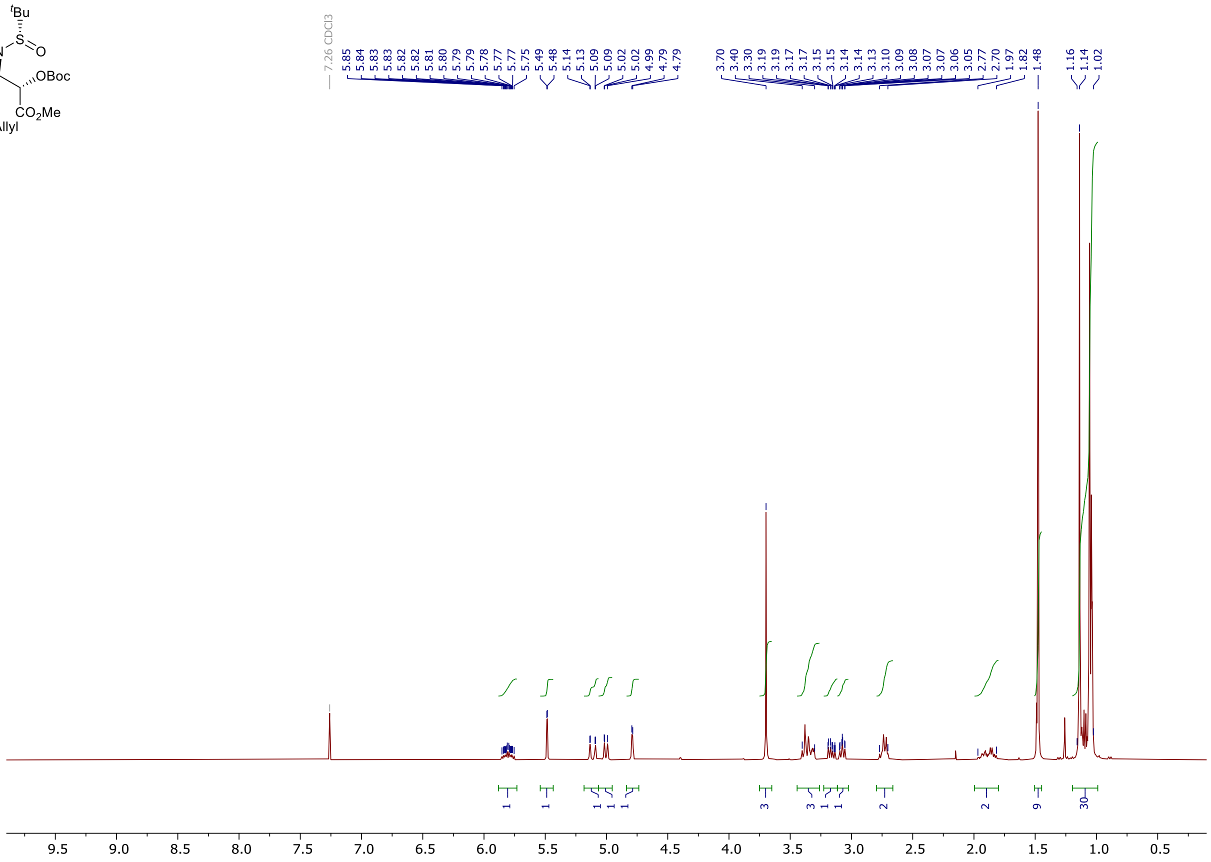

^{13}C{^{1}H} NMR ($CDCl_3$, 100 MHz)

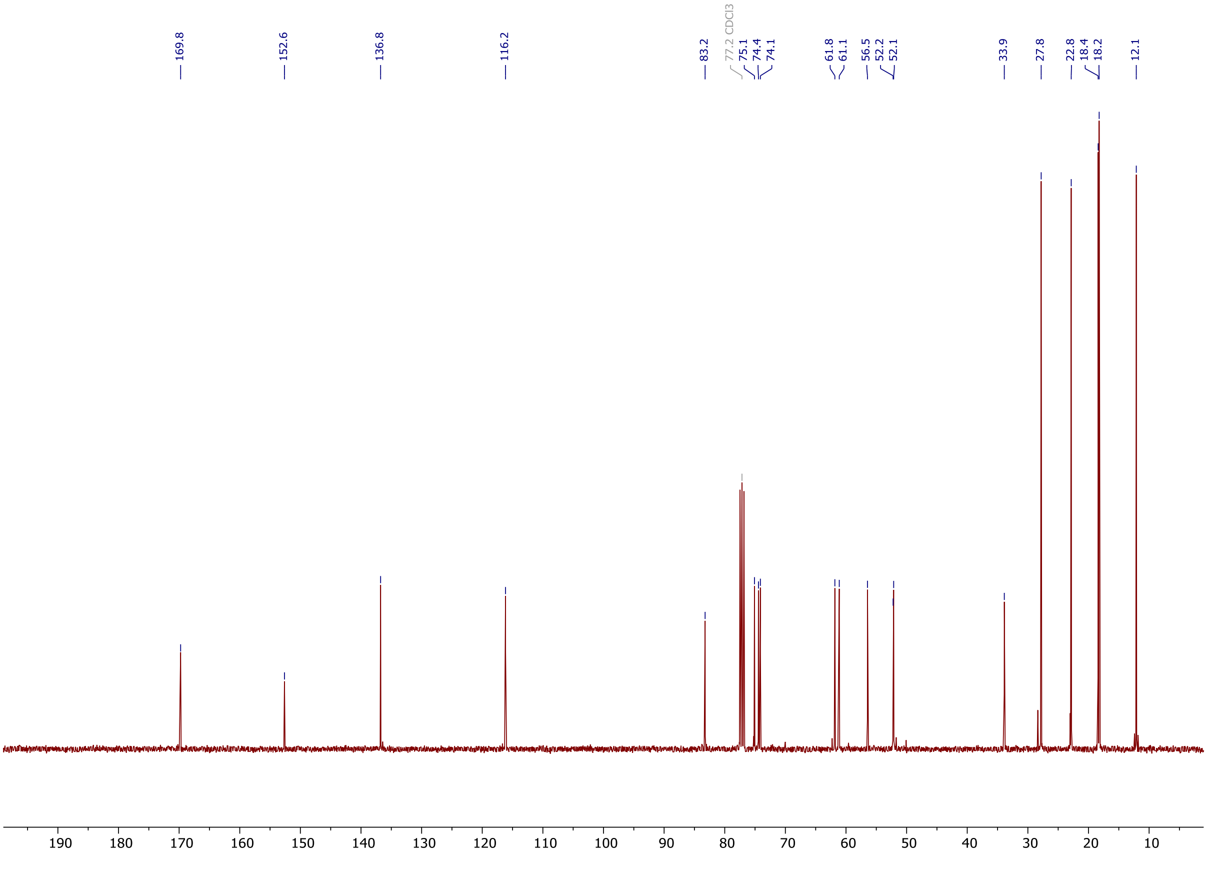

(1*R*,2*S*,7*R*,7a*S*,*S*S)-1-(*tert*-Butylsulfinyl)amino-2-(*tert*-butoxycarbonyl)oxy-7-triisopropylsilyloxypyrrolizin-3-one 28

^{1}H NMR ($CDCl_3$, 500 MHz)

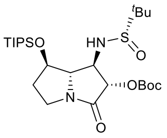

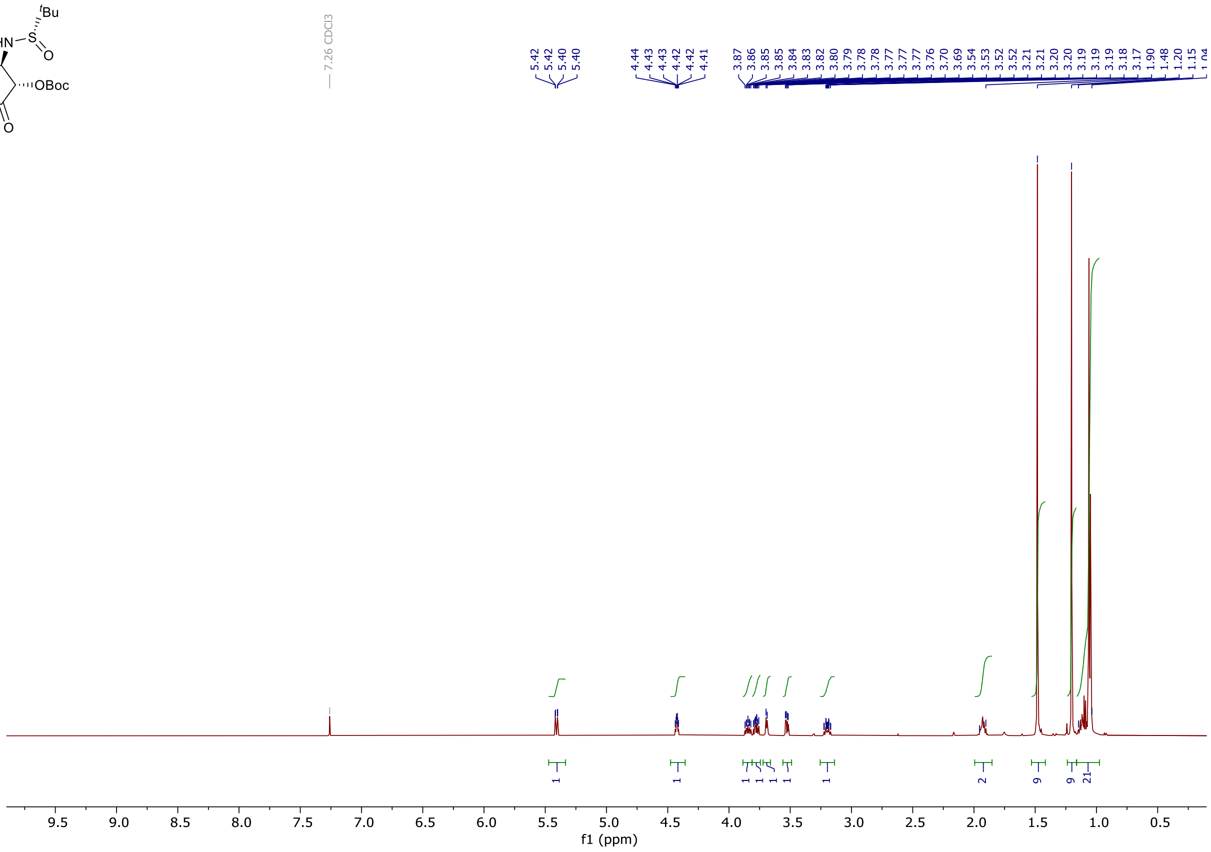

^{13}C{^{1}H} NMR ($CDCl_3$, 125 MHz)

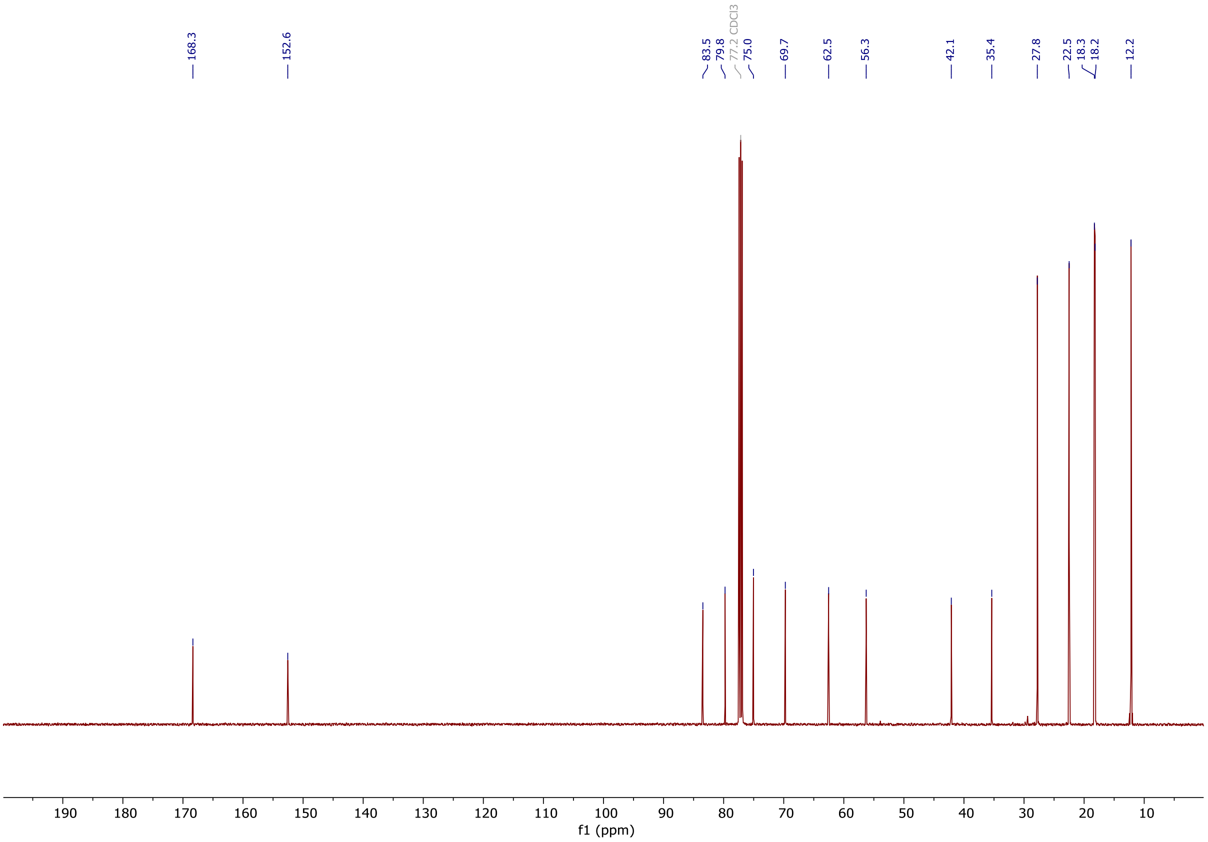

(1*R*,2*S*,7*R*,7a*S*,*S*S)-1-(*tert*-Butylsulfinyl)amino-2-(*tert*-butoxycarbonyl)oxy-7-hydroxypyrrolizin-3-one 29

^{1}H NMR (CDCl$_3$, 400 MHz)

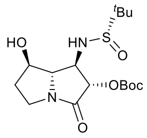

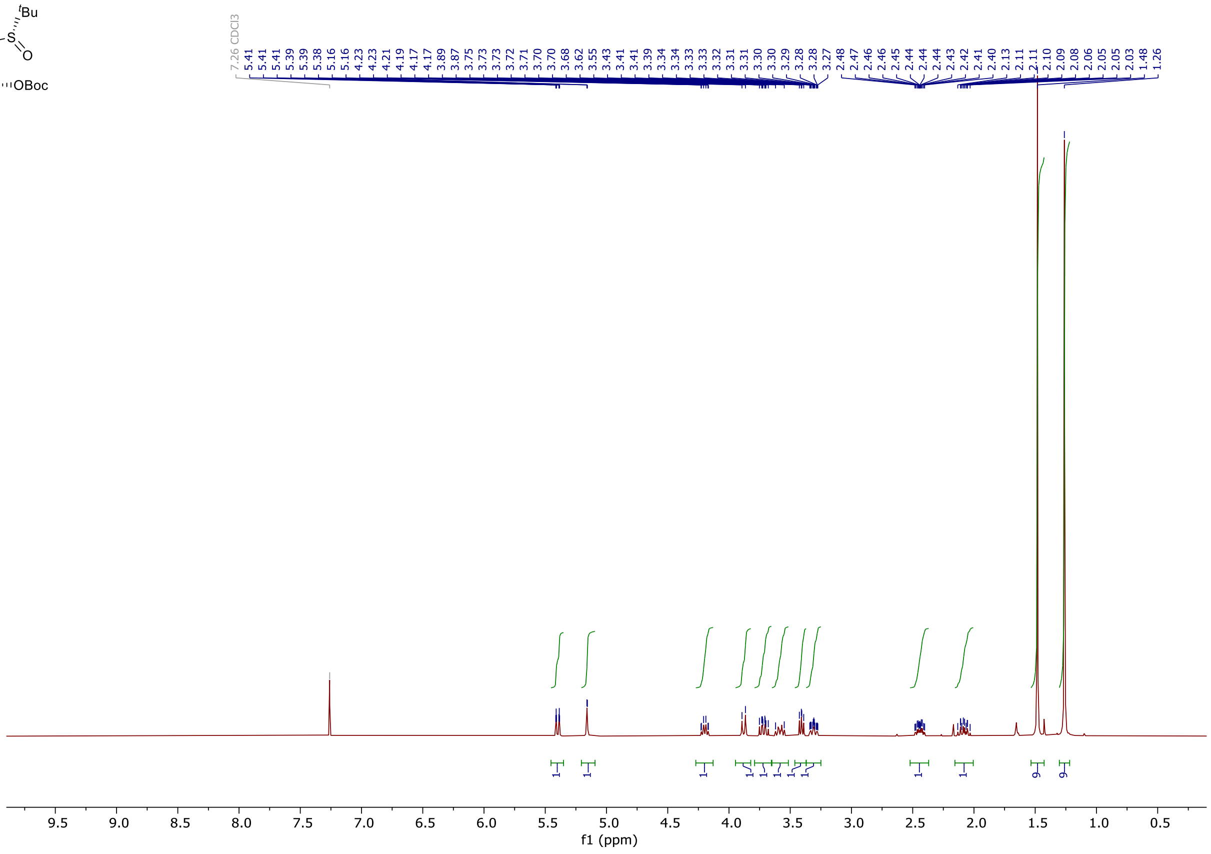

^{13}C{^{1}H} NMR (CDCl$_3$, 100 MHz)

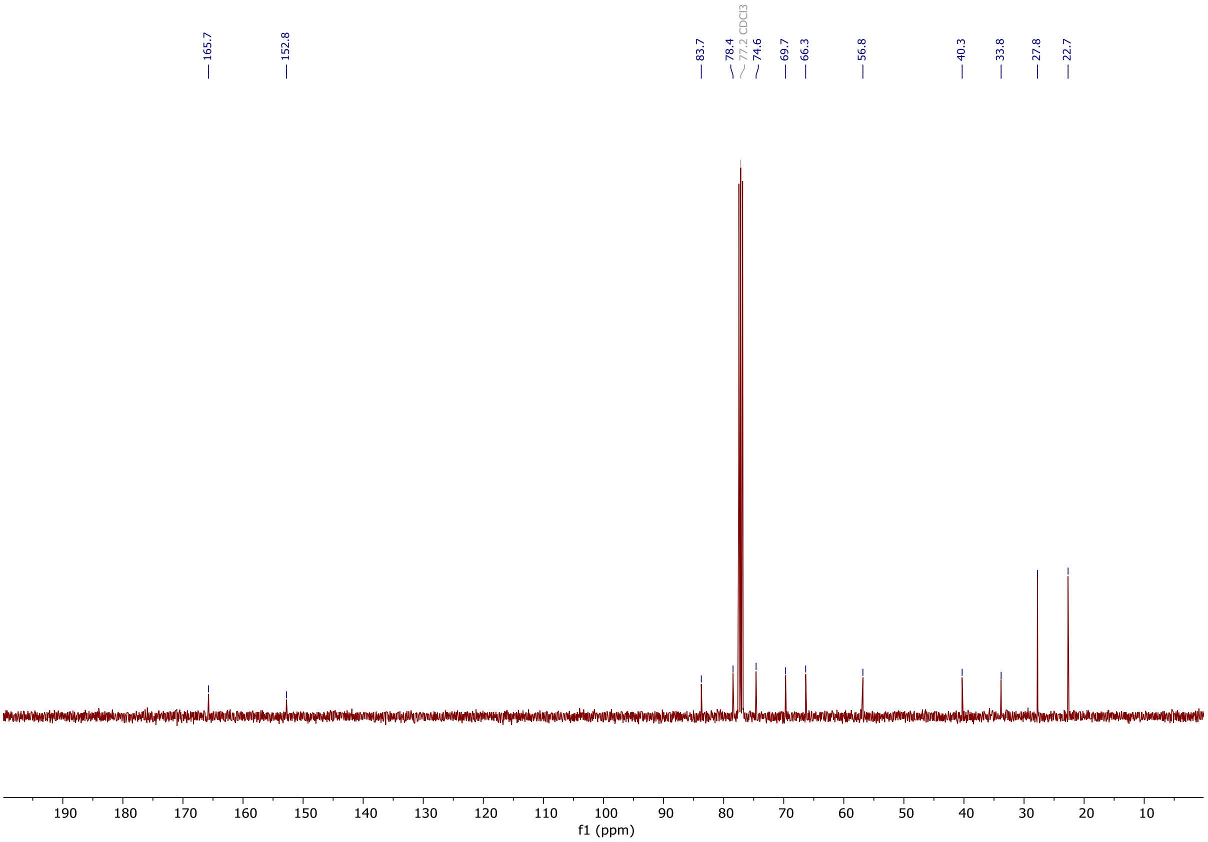

(1*R*,2*R*,4*S*,7*R*,7a*S*,*S*S)-1-(*tert*-Butylsulfinyl)amino-2-(*tert*-butoxycarbonyl)oxy-7-hydroxypyrrolizine borane complex 30·BH3

^{1}H NMR (CDCl3, 400 MHz)

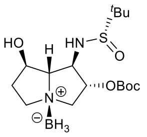

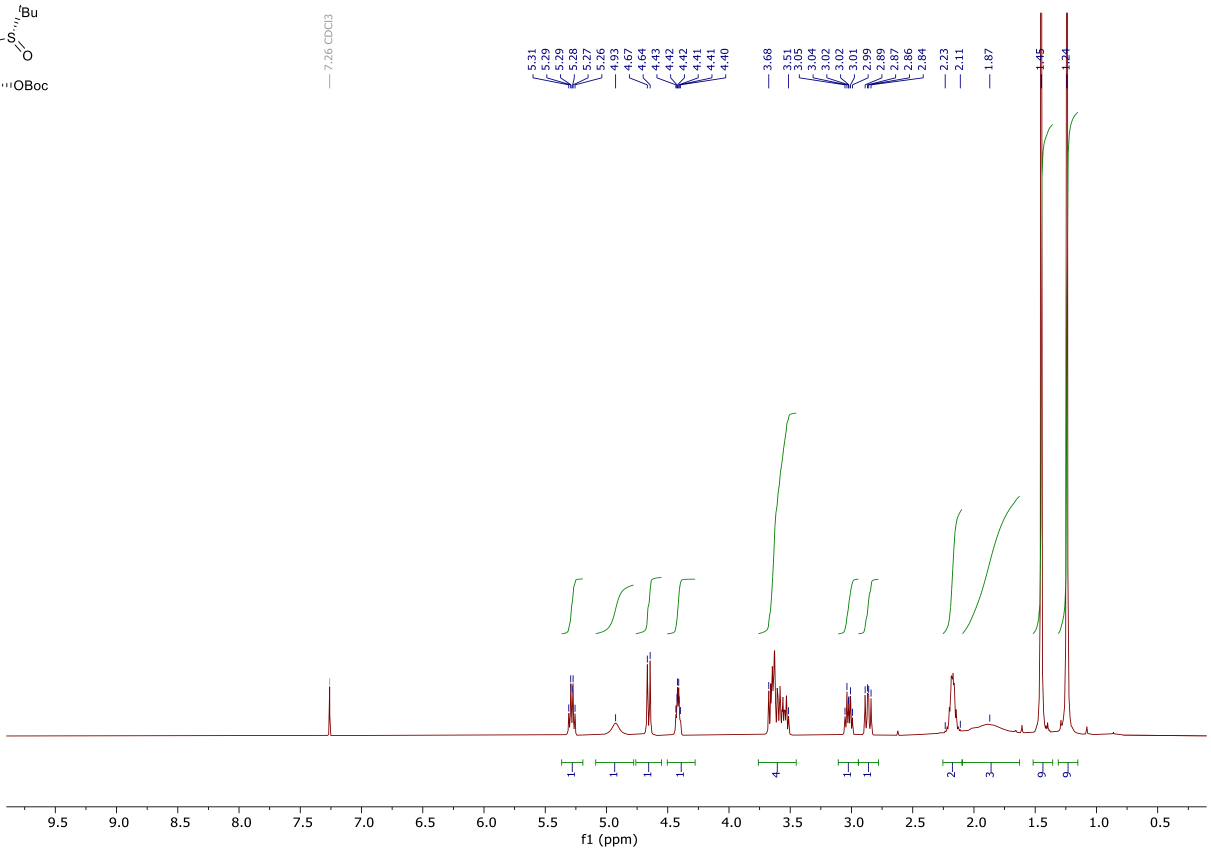

^{13}C{^{1}H} NMR (CDCl3, 100 MHz)

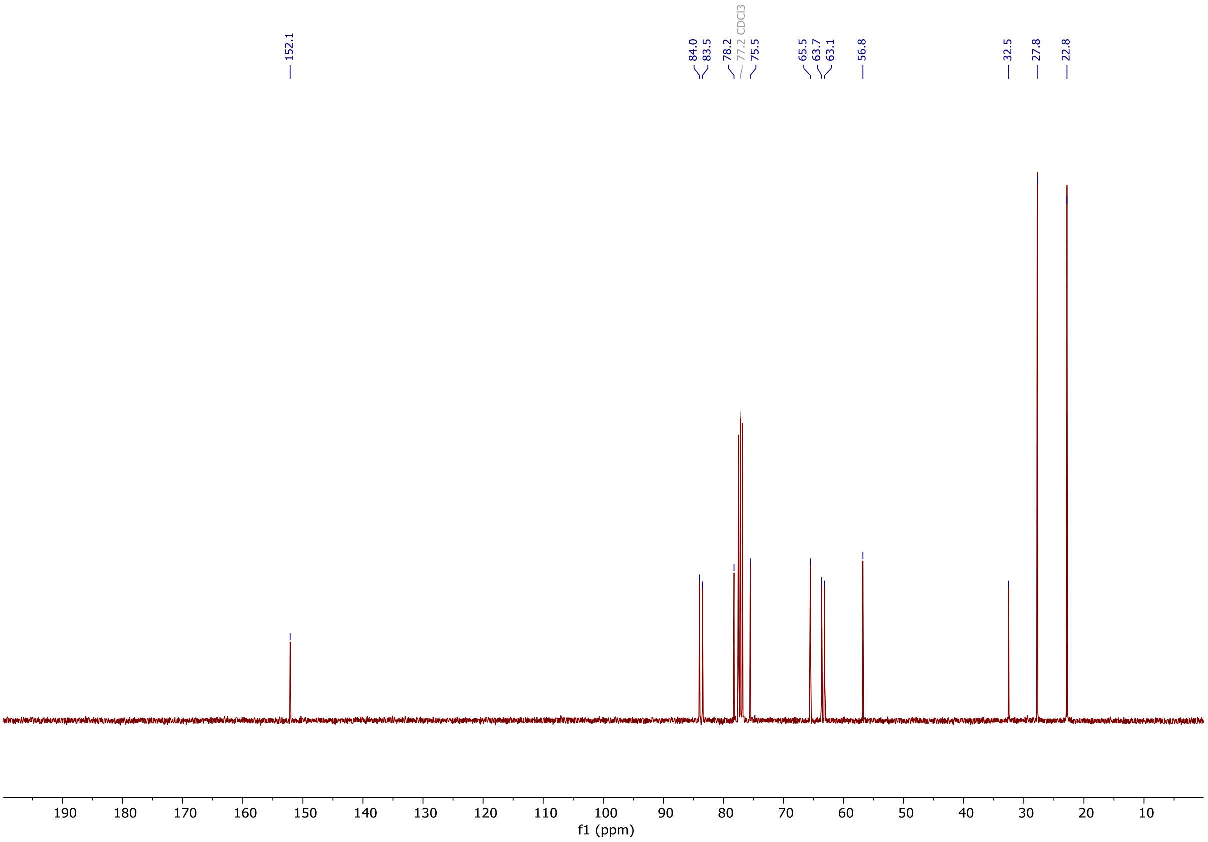

(1*R*,2*R*,7*R*,7a*S*,*S*S)-1-(*tert*-Butylsulfinyl)amino-2-(*tert*-butoxycarbonyl)oxy-7-hydroxypyrrolizine 30

^{1}H NMR (CDCl$_3$, 400 MHz)

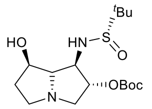

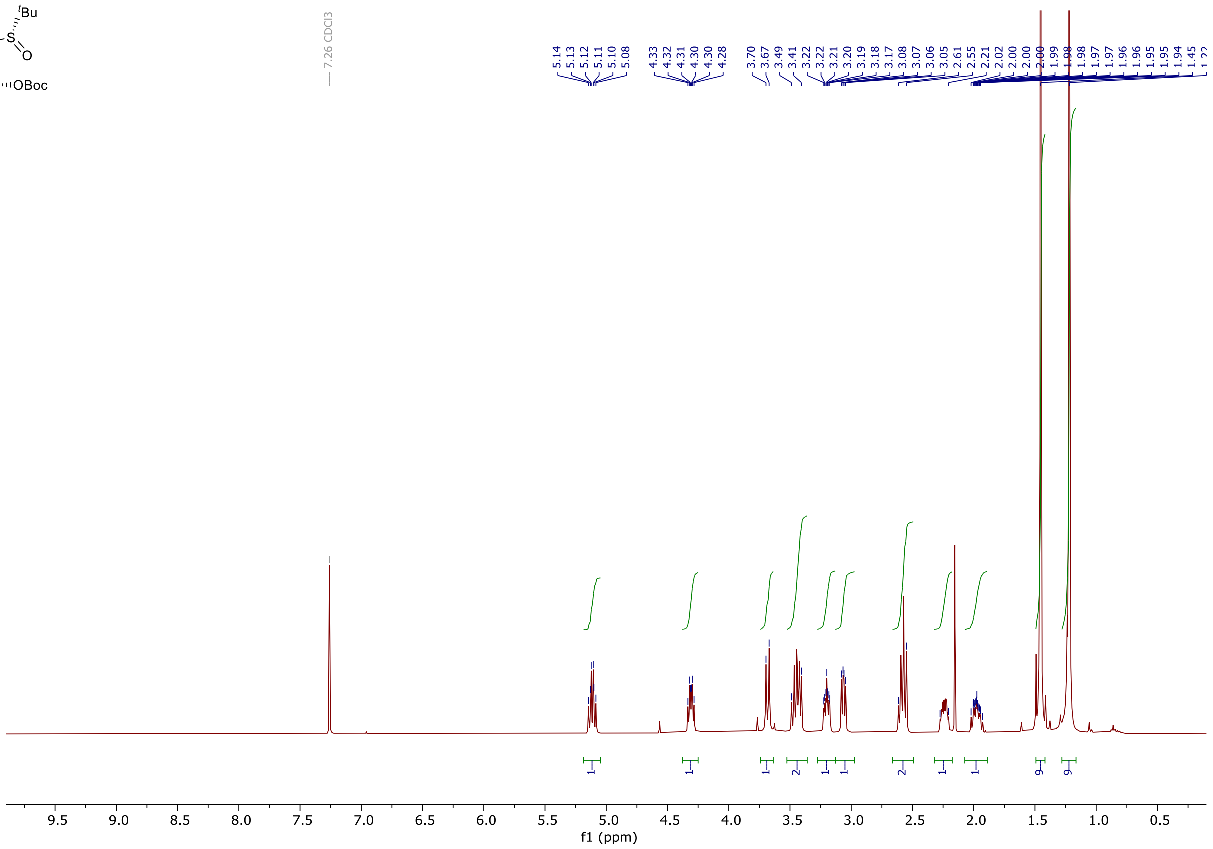

^{13}C{^{1}H} NMR (CDCl$_3$, 100 MHz)

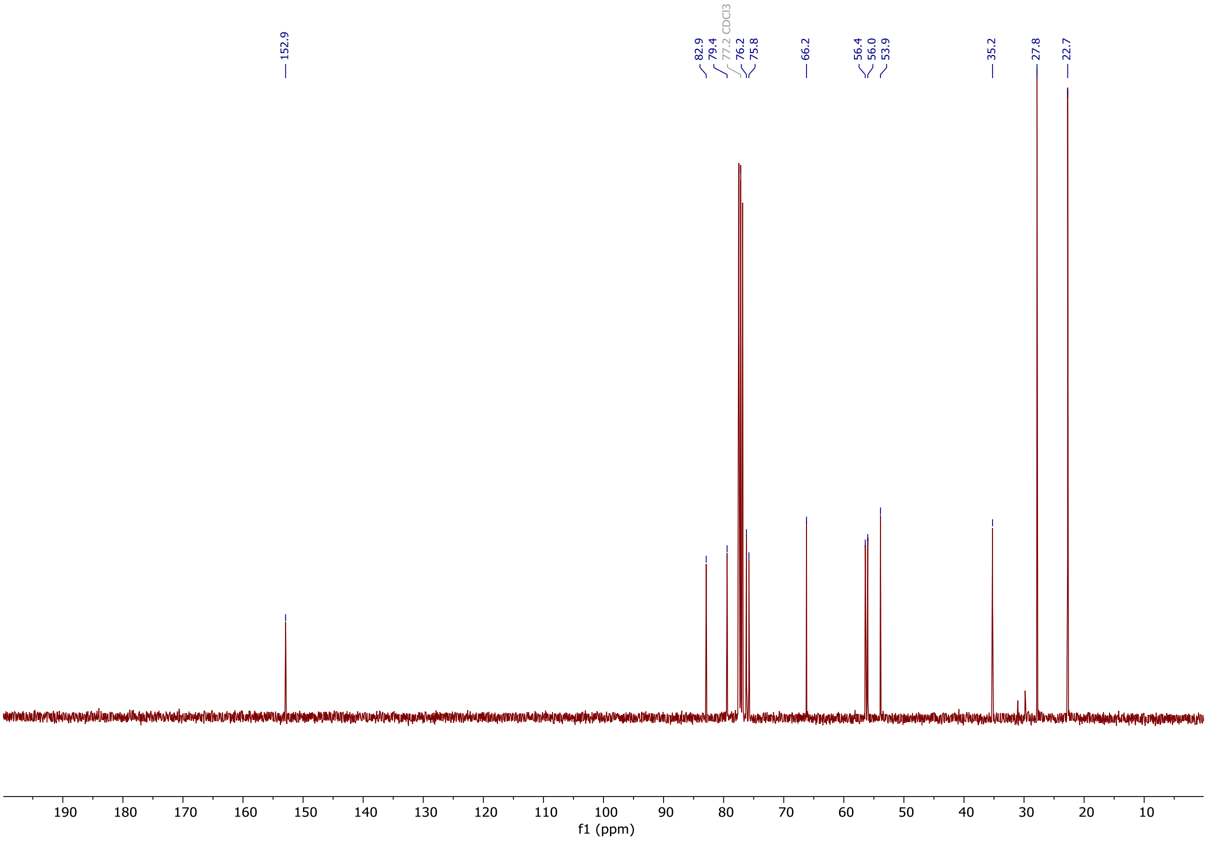

(1*R*,2*R*,7*S*,7a*S*,*S*S)-1-(*tert*-Butylsulfinyl)amino-2,7-epoxypyrrolizidine 32

^{1}H NMR ($CDCl_3$, 400 MHz)

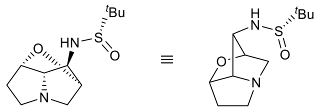

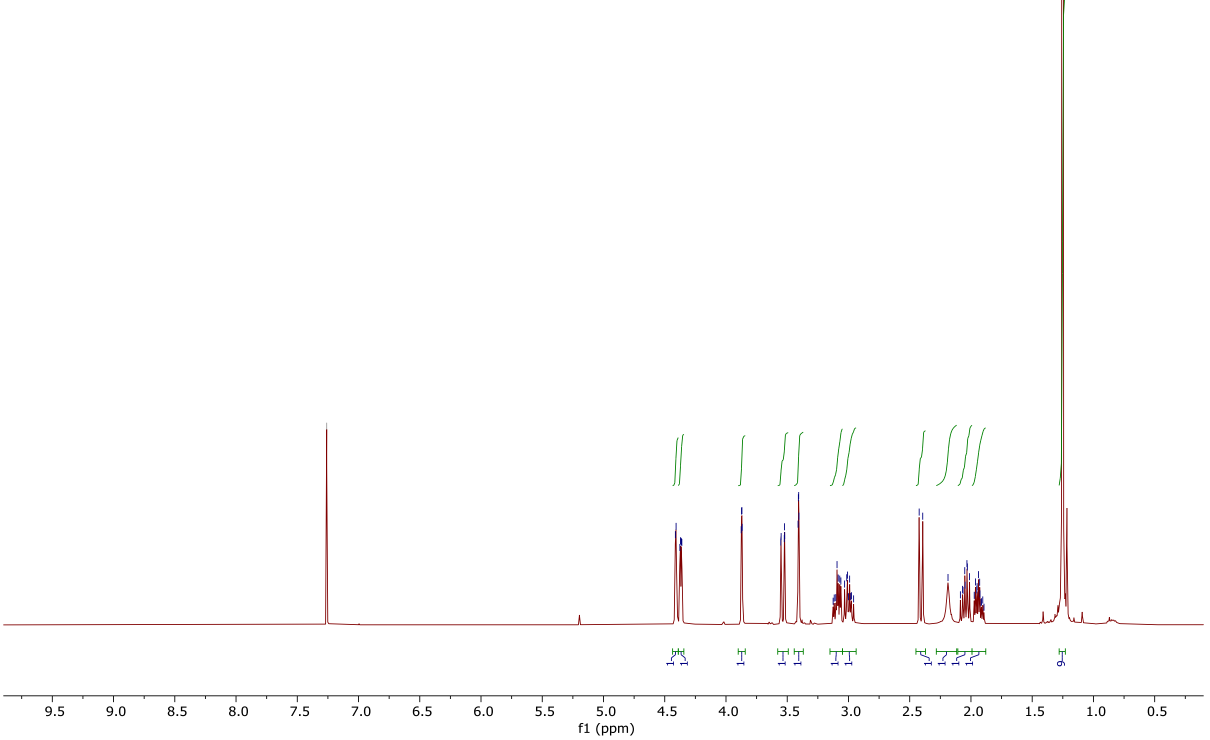

(1*R*,2*R*,7*S*,7a*S*)-1-Amino-2,7-epoxypyrrolizidine dihydrochloride [norloline dihydrochloride] 33·2HCl

^{1}H NMR (D_2O, 400 MHz)

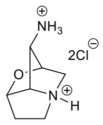

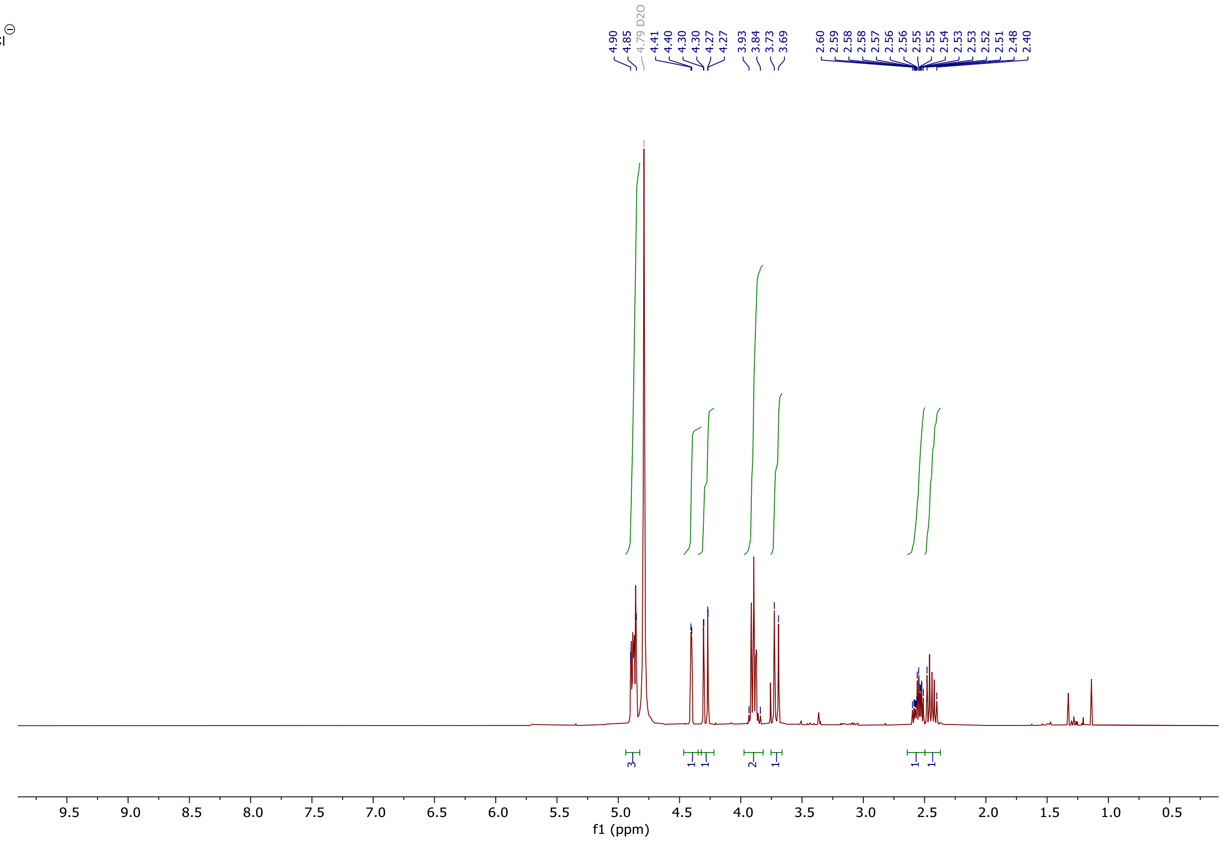

^{13}C{^{1}H} NMR (D_2O, 100 MHz)

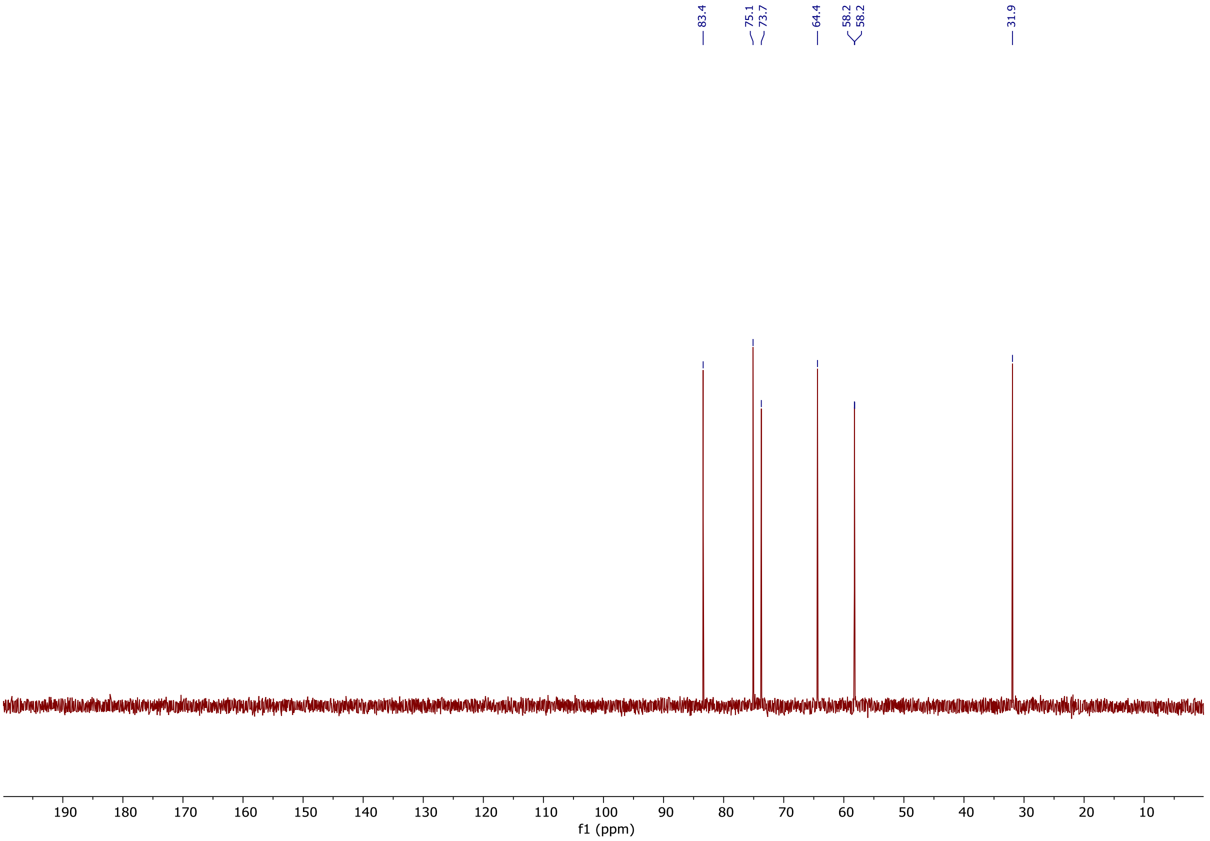

(1*R*,2*R*,7*S*,7a*S*)-1-Amino-2,7-epoxypyrrolizidine [norloline] 33

^{1}H NMR ($CDCl_3$, 400 MHz)

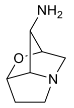

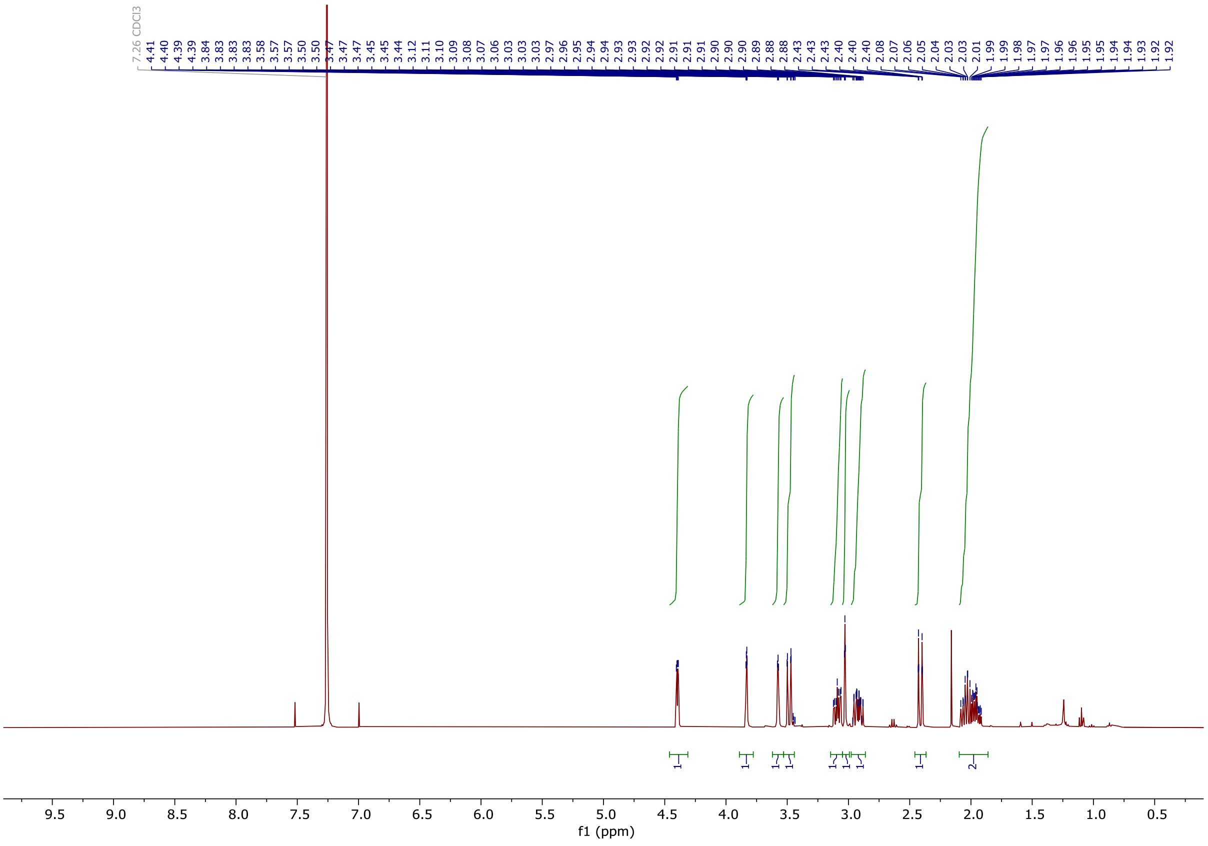

^{13}C{^{1}H} NMR ($CDCl_3$, 100 MHz)

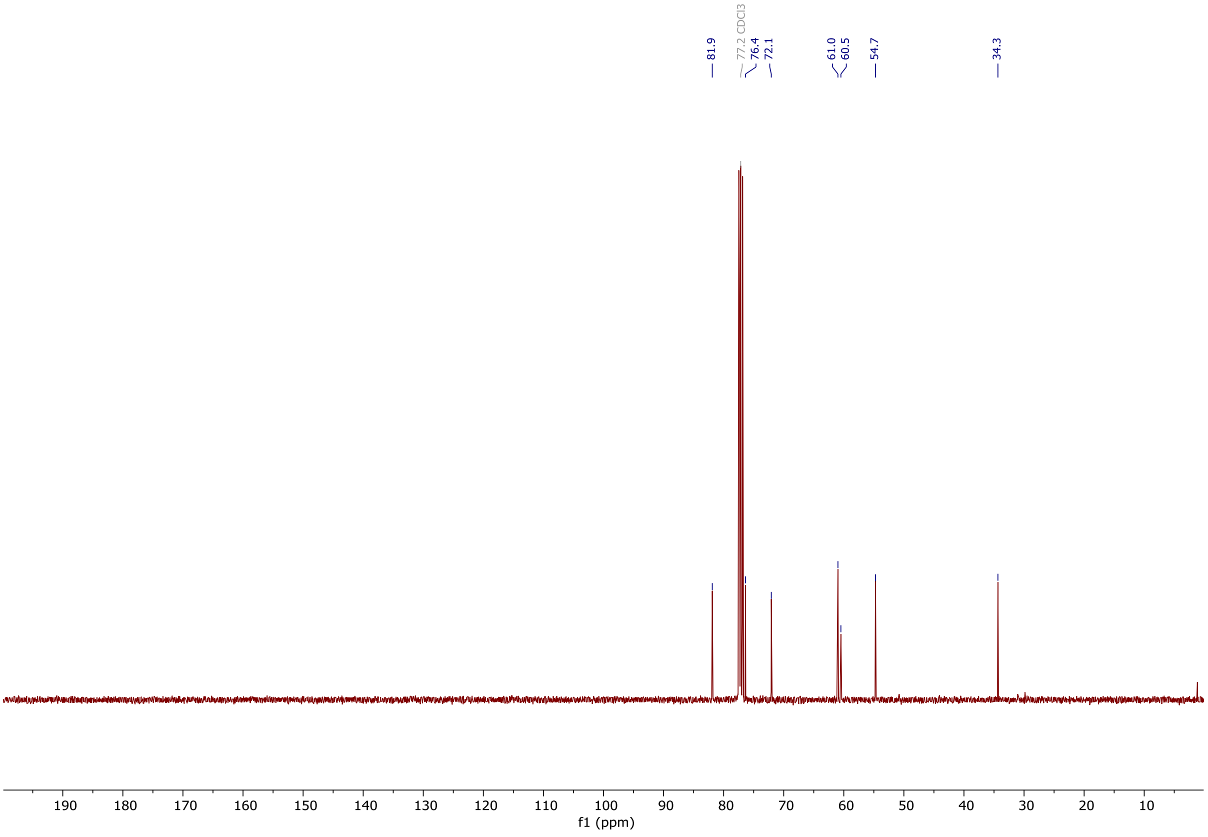

(1*R*,2*R*,7*S*,7a*S*)-1-Acetamido-2,7-epoxypyrrolizidine [*N*-acetyl norloline] 34

^{1}H NMR (CDCl$_3$, 400 MHz)

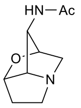

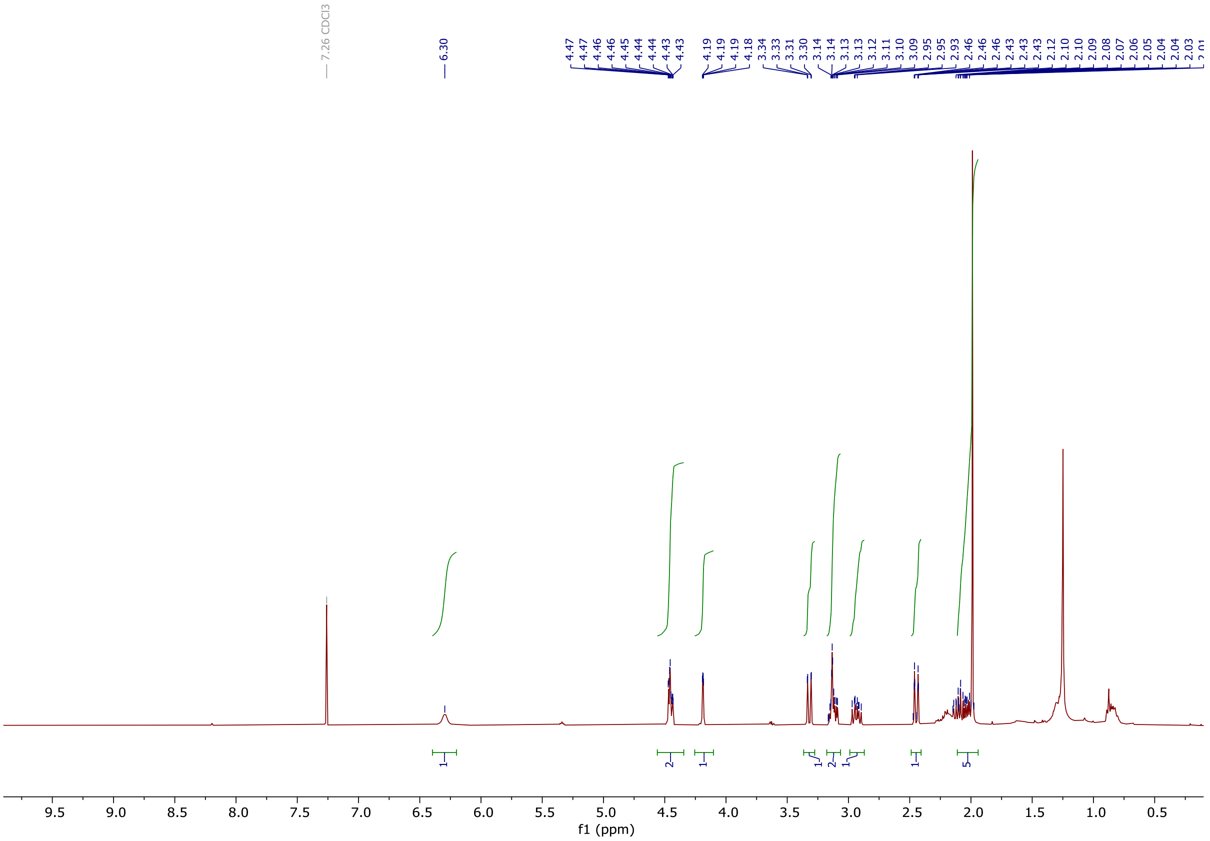

^{13}C{^{1}H} NMR (CDCl$_3$, 100 MHz)

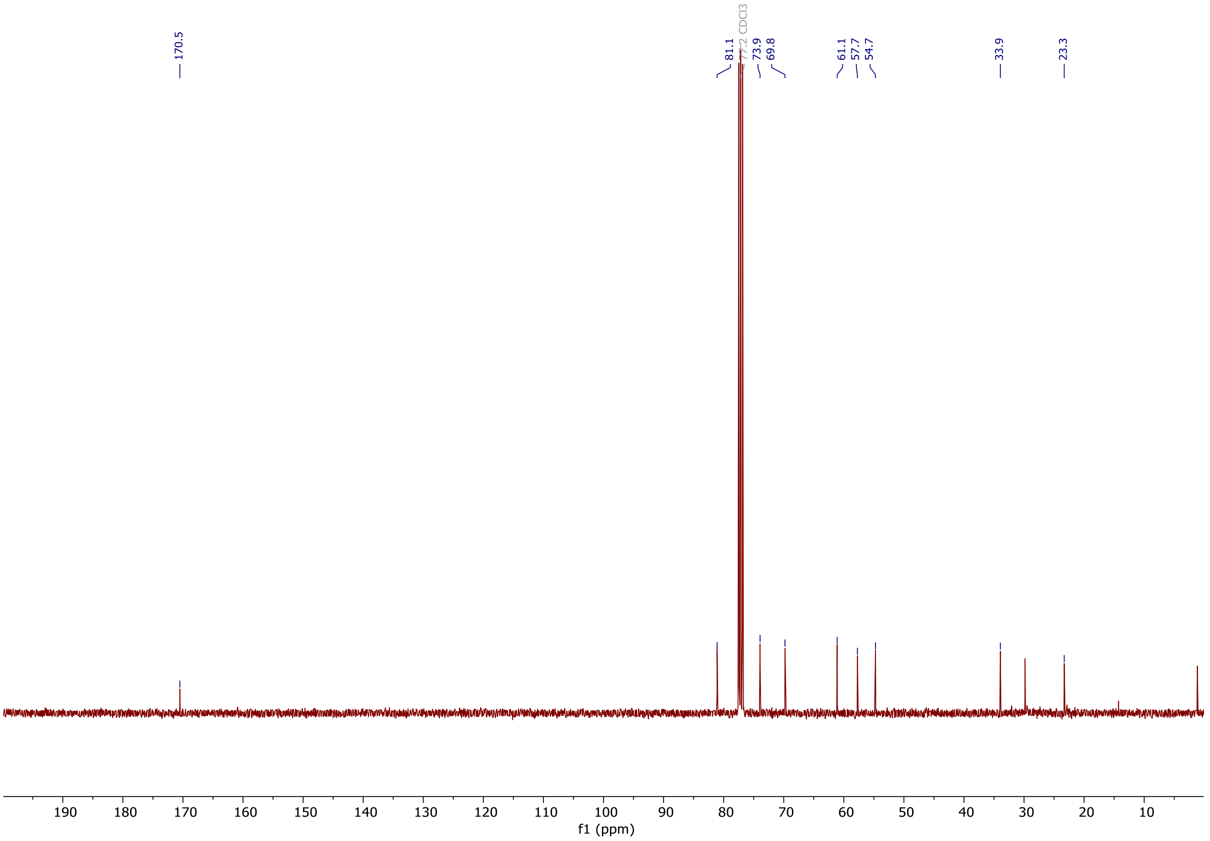

(1*R*,2*R*,7*S*,7a*S*)-1-(*N*,*N*-Dimethylamino)-2,7-epoxypyrrolizidine·dihydrochloride [*N*-methyl loline dihydrochloride] 35·2HCl

^{1}H NMR (D_2O, 400 MHz)

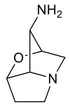

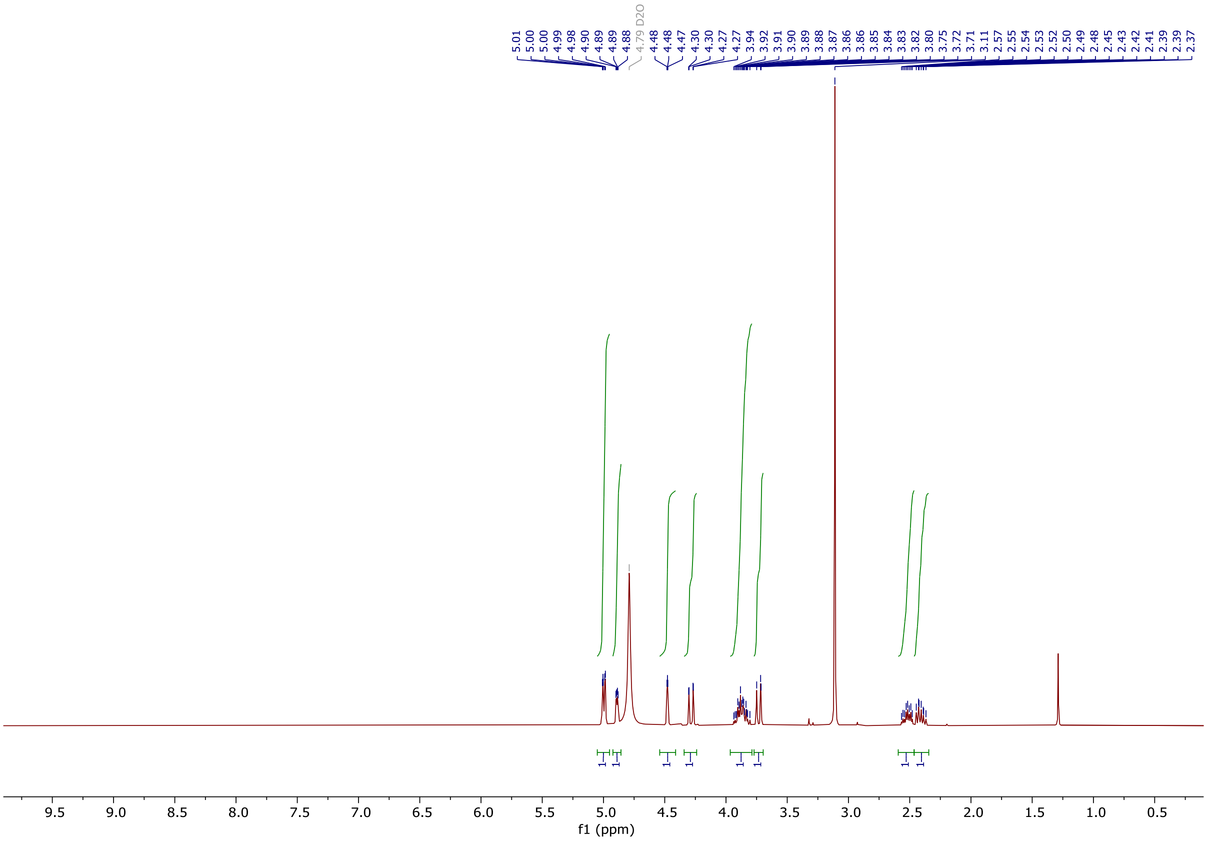

^{13}C{^{1}H} NMR (D_2O, 100 MHz)

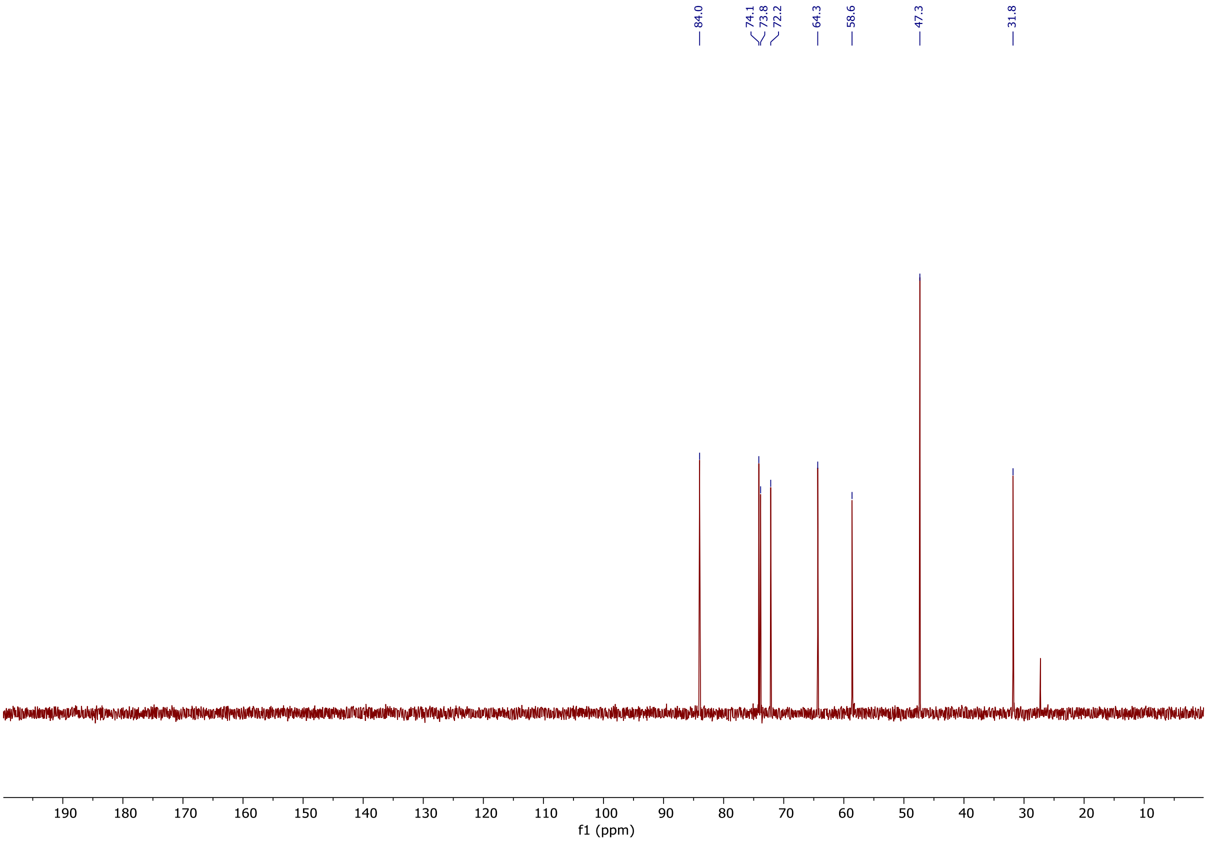

(1*R*,2*R*,7*S*,7a*S*)-1-(*N*,*N*-Dimethylamino)-2,7-epoxypyrrolizidine·[*N*-methyl loline] 35

^{1}H NMR (CDCl$_3$, 400 MHz)

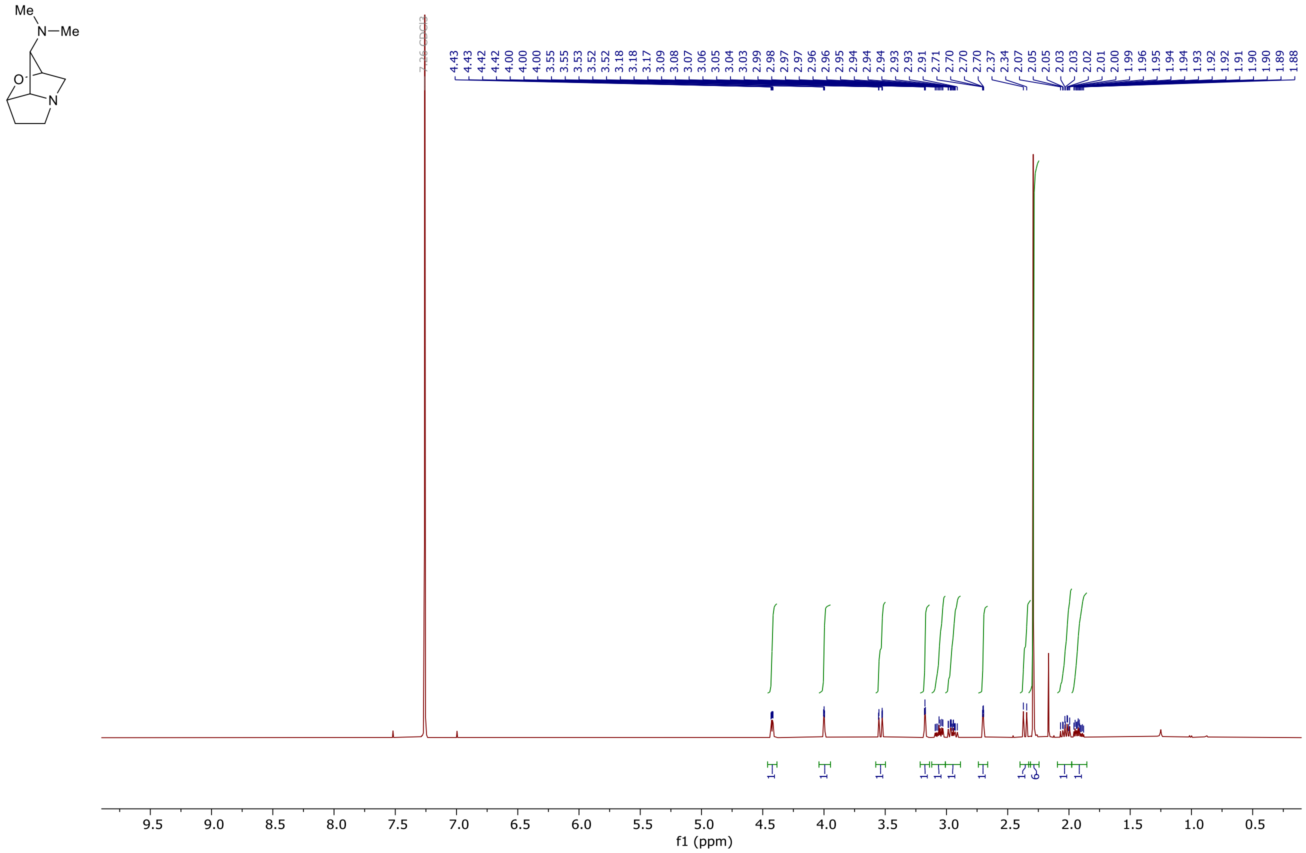

^{13}C{^{1}H} NMR (CDCl$_3$, 100 MHz)

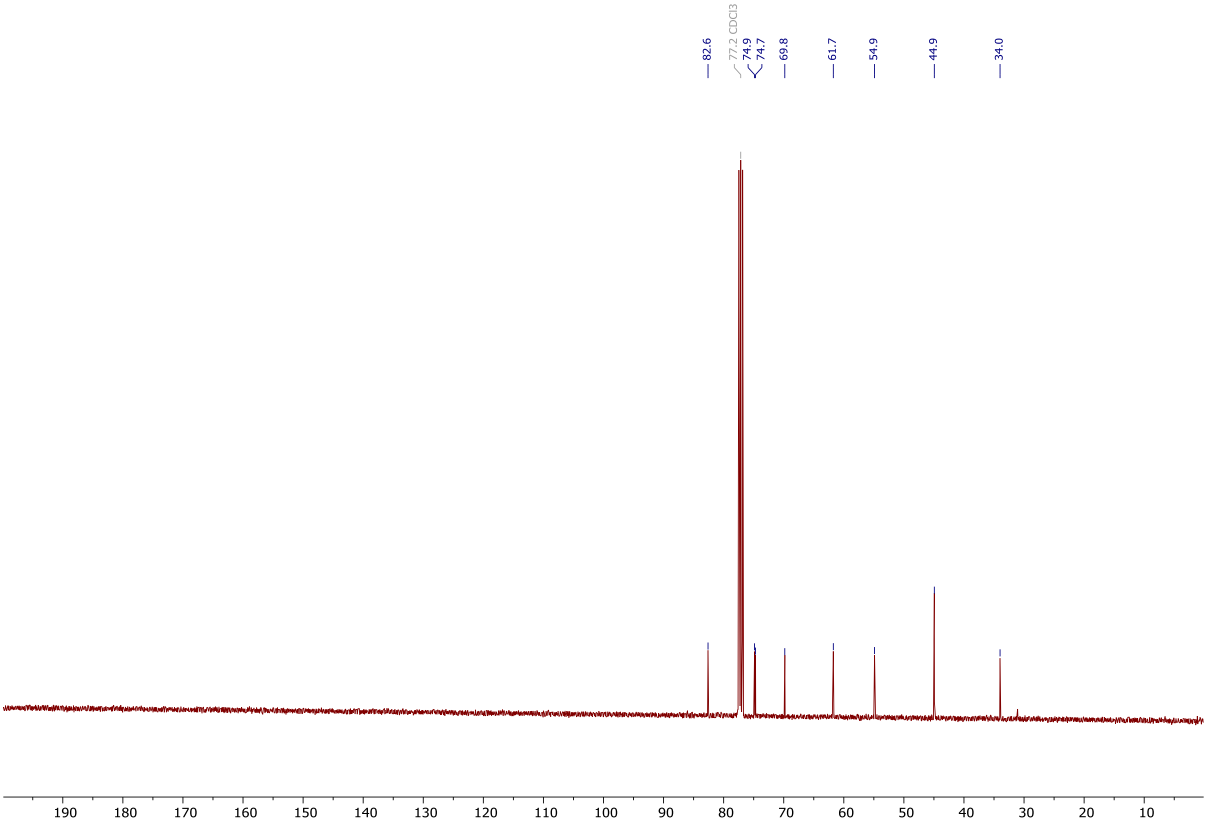

(1*R*,2*R*,7*S*,7a*S*)-1-(*tert*-Butoxycarbonyl)amino-2,7-epoxypyrrolizidine [*N*-Boc norloline] 36

^{1}H NMR ($CDCl_3$, 400 MHz)

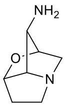

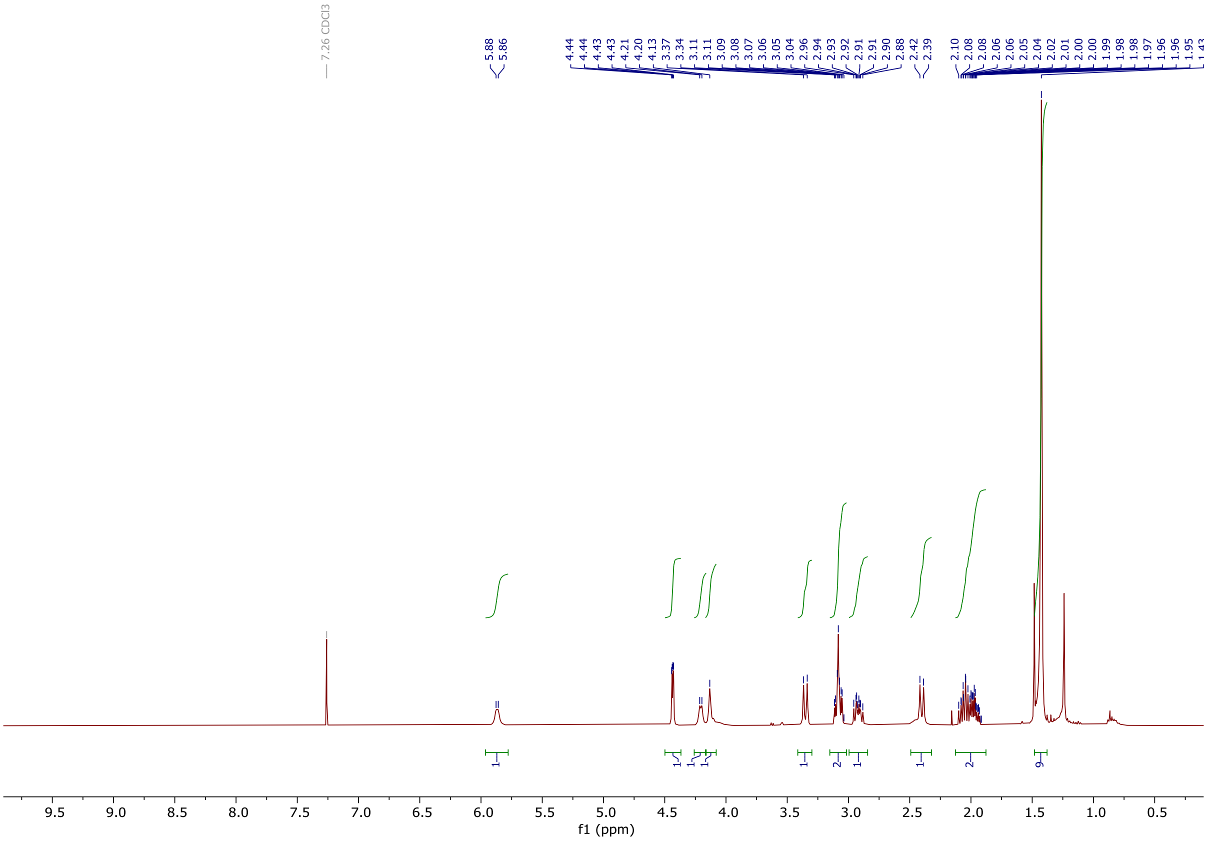

^{13}C{^{1}H} NMR ($CDCl_3$, 100 MHz)

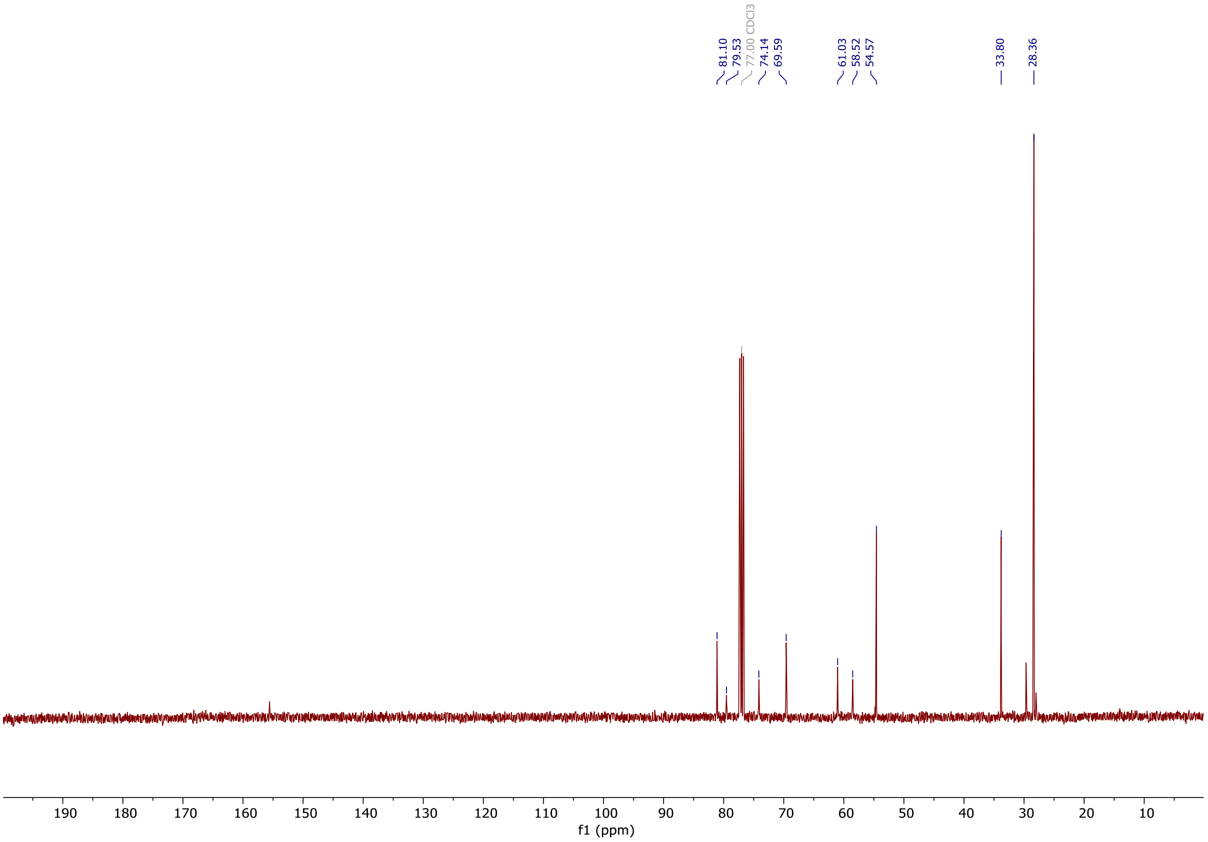

(1*R*,2*R*,7*S*,7a*S*)-1-[*N*-Methylamino]-2,7-epoxypyrrolizidine dihydrochloride [loline dihydrochloride] 37·2HCl

^{1}H NMR (D_2O, 400 MHz)

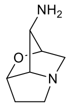

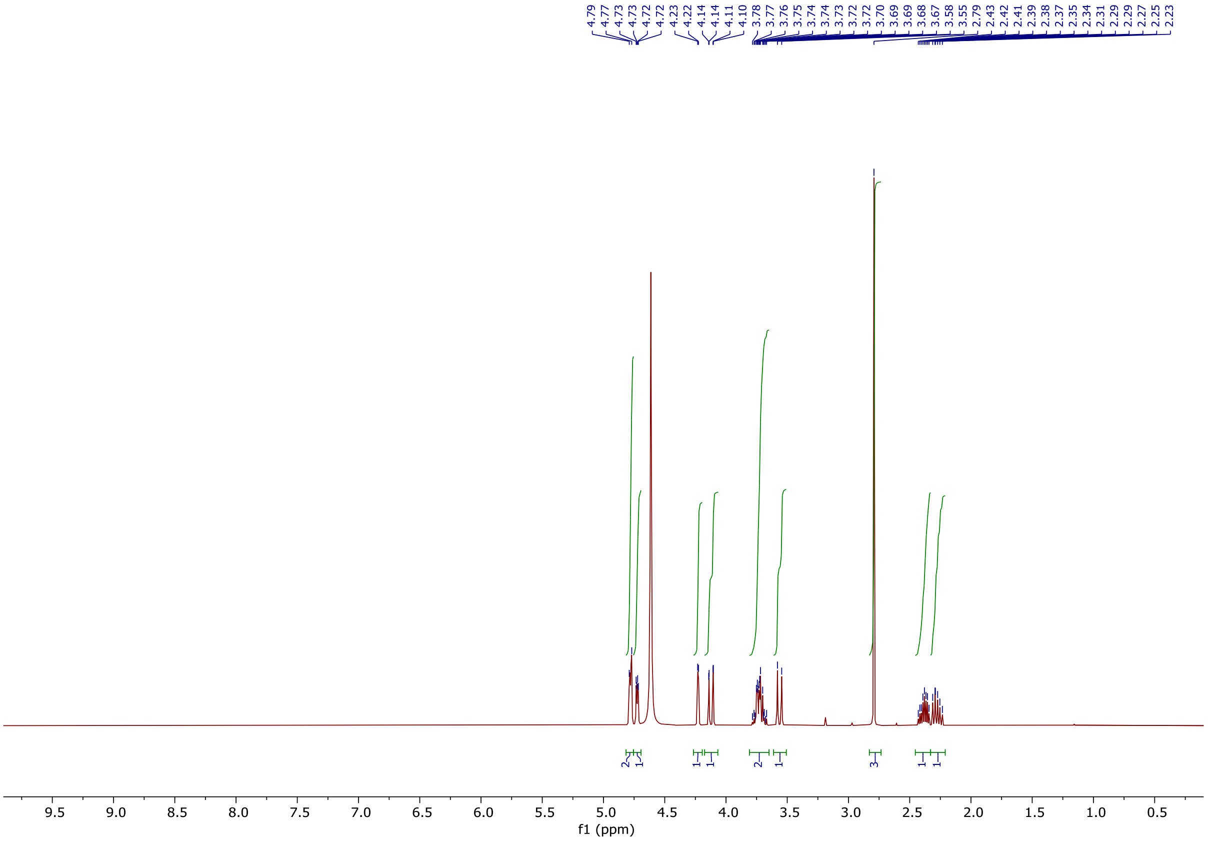

$^{13}C\{^{1}H\}$ NMR (D_2O, 100 MHz)

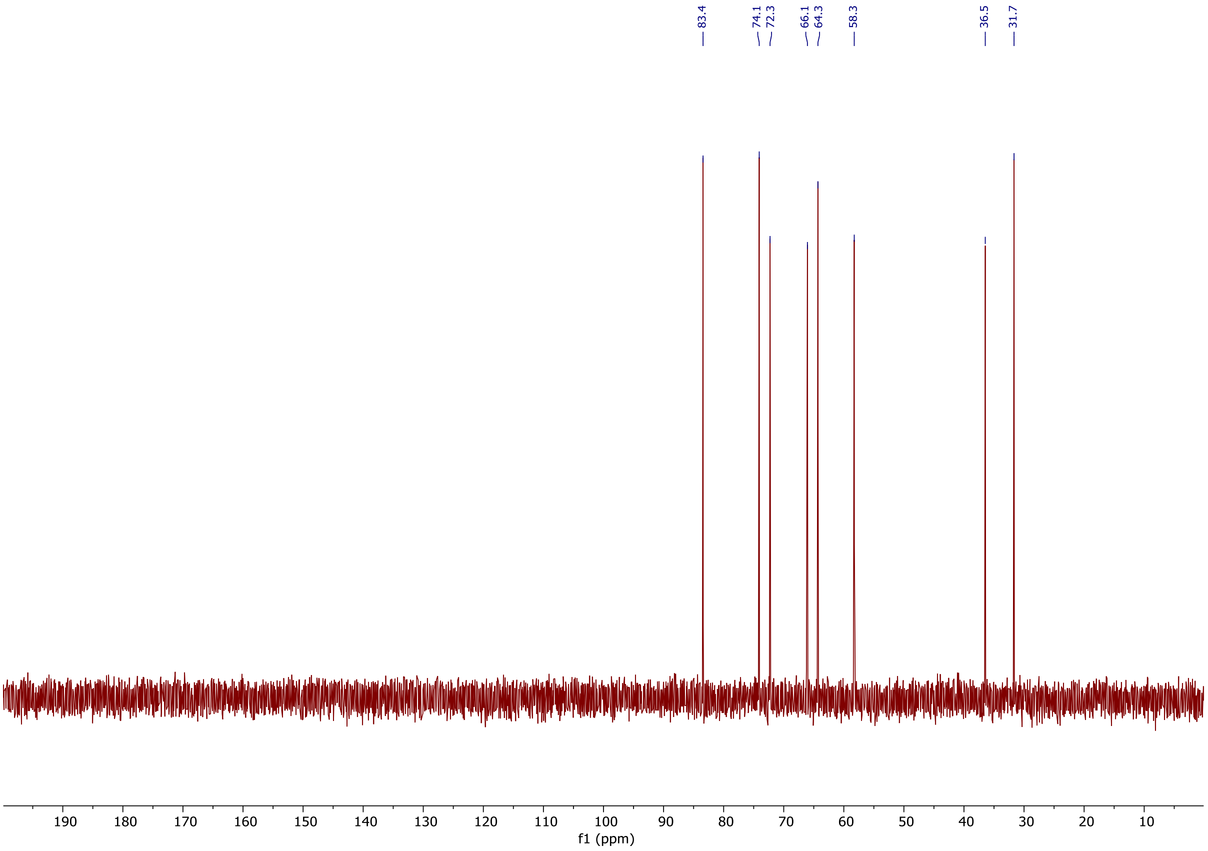

(1*R*,2*R*,7*S*,7a*S*)-1-[*N*-methylamino]-2,7-epoxypyrrolizidine [loline] 37

^{1}H NMR ($CDCl_3$, 500 MHz)

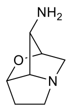

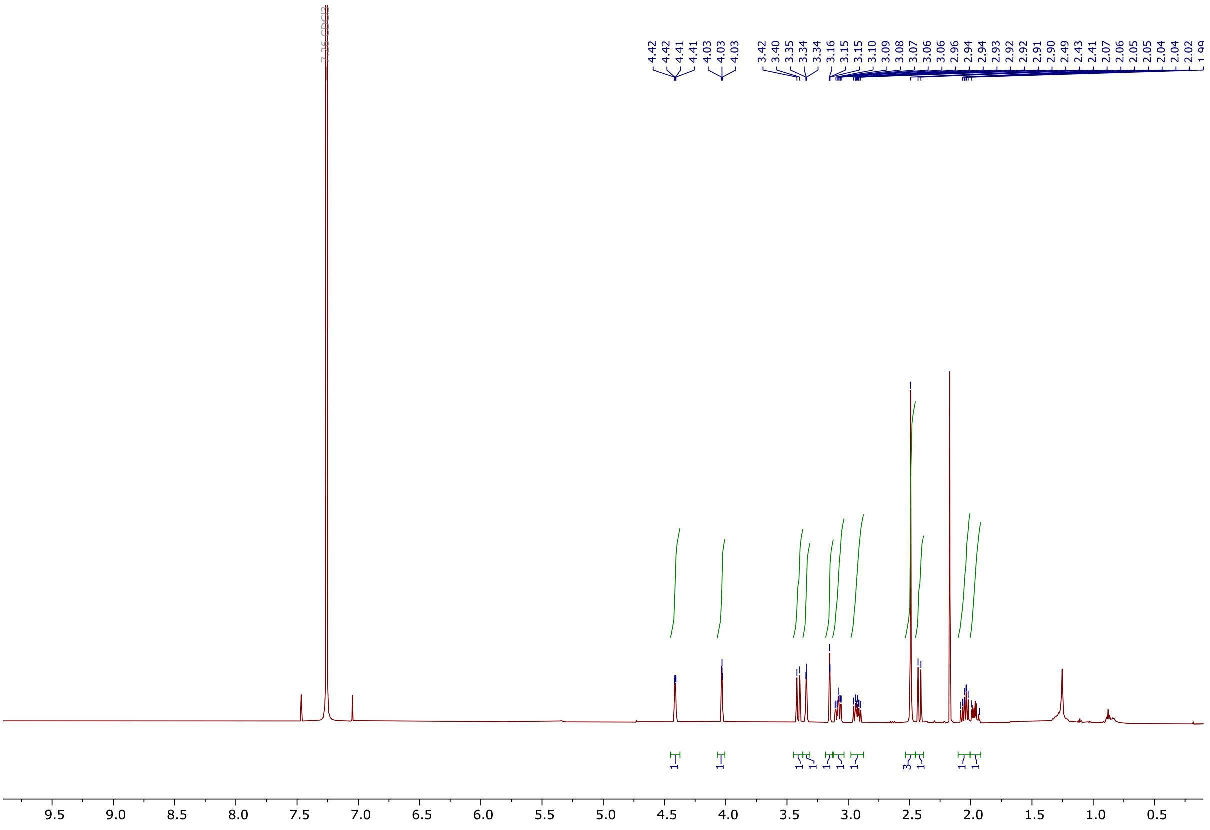

^{13}C{^{1}H} NMR ($CDCl_3$, 125 MHz)

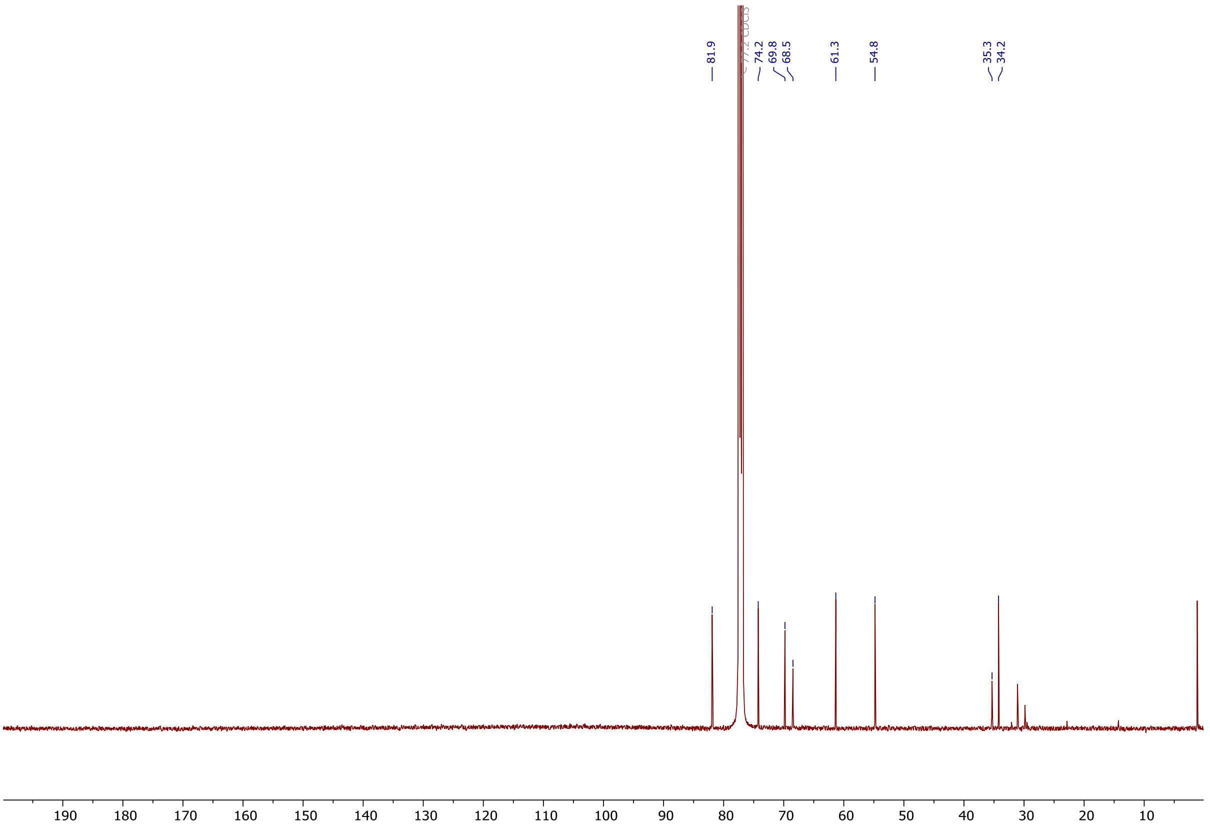

5. Data Comparison for the Loline Alkaloids

Compiled References by Alkaloid (in Chronological Order)

Norloline, $R^1 = R^2 = H$
N-Acetyl loline, $R^1 = Me$, $R^2 = Ac$
N-Methyl loline, $R^1 = R^2 = Me$
Loline, $R^1 = Me$, $R^2 = H$

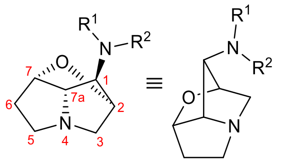

	Reference	Norloline	*N*-Acetyl norloline	*N*-Methyl loline	Loline•2HCl	Loline
1	*J. Nat. Prod.* **1989**, *52*, 810	x	x	x	x	x
2	*Chem. Commun.* **2000**, 1263				x	
3	*J. Chem. Soc., Perkin Trans. 1* **2001**, 1831				x	
4	*Org. Lett.* **2011**, *13*, 1246		x			
5	*Nat. Chem.* **2011**, *3*, 543	x			x	
6	*Chem. Commun.* **2016**, *52*, 561		x			

^{1}H and ^{13}C NMR Data Comparison for Norloline

	Ref 1		Ref 5		33	
No.	δ_C (75 MHz)	δ_H (300 MHz)	δ_C (75 MHz)	δ_H (300 MHz)	δ_C (100 MHz)	δ_H (400 MHz)
1	60.5	3.48	60.5 (0.0)	3.60 (+0.12)	60.3 (−0.2)	3.58 (+0.10)
2	76.2	3.72	76.2 (0.0)	3.84 (+0.12)	76.2 (0.0)	3.83 (+0.11)
3-A	60.8	2.29	60.8 (0.0)	2.42 (+0.13)	60.8 (0.0)	2.41 (+0.12)
3-B		3.38		3.50 (+0.12)		3.48 (+0.10)
5-A	54.5	2.80	54.5 (0.0)	2.93 (+0.13)	54.6 (+0.1)	2.92 (+0.12)
5-B		2.98		3.10 (+0.12)		3.09 (+0.11)
6-A	34.1	1.84	34.1 (0.0)	1.97 (+0.13)	34.2 (+0.1)	1.96 (+0.12)
6-B		1.94		2.03 (+0.09)		2.05 (+0.11)
7	81.7	4.29	81.7 (0.0)	4.40 (+0.11)	81.7 (0.0)	4.40 (+0.11)
7a	71.9	2.92	71.9 (0.0)	3.05 (+0.13)	71.9 (0.0)	3.03 (+0.11)

^{1}H and ^{13}C NMR Data Comparison for *N*-Acetyl Norloline

	Ref 1		Ref 4		Ref 6		34	
No.	δ_C (75 MHz)	δ_H (300 MHz)	δ_C (75 MHz)	δ_H (300 MHz)	δ_C (100 MHz)	δ_H (400 MHz)	δ_C (100 MHz)	δ_H (400 MHz)
1	57.6	4.41	57.6 (0.0)	4.44 (+0.03)	57.6 (0.0)	4.41 (0.00)	57.6 (0.0)	4.44 (+0.03)
2	73.8	4.17	73.8 (0.0)	4.18 (+0.01)	73.8 (0.0)	4.17 (0.00)	73.8 (0.0)	4.19 (+0.02)
3-A	60.9	2.42	60.9 (0.0)	2.45 (+0.03)	61.0 (+0.1)	2.43 (+0.01)	60.9 (0.0)	2.45 (+0.03)
3-B		3.29		3.32 (+0.03)		3.31 (+0.02)		3.32 (+0.03)
5-A	54.6	2.90	54.6 (0.0)	2.89 (−0.01)	54.6 (0.0)	2.93 (+0.03)	54.6 (0.0)	2.93 (+0.03)
5-B		3.10		3.13 (+0.03)		3.09 (−0.01)		3.13 (+0.03)
6-A	33.9	1.98	33.9 (0.0)	2.08 (+0.05)*	33.8 (−0.1)	1.98 (0.00)	33.8 (0.0)	2.04 (+0.06)
6-B		2.08				2.07 (−0.01)		2.10 (+0.02)
7	80.9	4.44	81.0 (+0.1)	4.47 (+0.03)	80.8 (−0.1)	4.43 (−0.01)	80.9 (0.0)	4.46 (+0.02)
7a	69.6	3.09	69.7 (+0.1)	3.12 (+0.03)	69.7 (+0.1)	3.15 (+0.06)	69.6 (0.0)	3.10 (+0.01)
NH	-	6.21	-	n.r.	-	n.r.	-	6.30 (+0.09)
CO*Me*	23.1	1.97	23.2 (+0.1)	1.99 (+0.02)	23.1 (0.0)	1.95 (−0.02)	23.2 (+0.1)	1.99 (+0.02)
*C*OMe	170.2	-	170.3 (+0.1)	-	170.5 (+0.3)	-	170.4 (+0.2)	-

^{1}H and ^{13}C NMR Data Comparison for *N*-Methyl Loline

	Ref 1		35	
No.	δ_C (75 MHz)	δ_H (300 MHz)	δ_C (125 MHz)	δ_H (500 MHz)
1	74.1	2.53	74.5 (+0.4)	2.70 (+0.17)
2	74.2	3.82	74.7 (+0.5)	4.00 (+0.18)
3-A	61.2	2.18	61.6 (+0.4)	2.36 (+0.18)
3-B		3.36		3.54 (+0.18)
5-A	54.3	2.78	54.7 (+0.4)	2.95 (+0.17)
5-B		2.89		3.06 (+0.17)
6-A	33.4	1.75	33.8 (+0.4)	1.92 (+0.17)
6-B		1.86		2.03 (+0.17)
7	82.0	4.25	82.4 (+0.4)	4.42 (+0.17)
7a	69.2	3.01	69.7 (+0.5)	3.18 (+0.17)
NMe	44.4	2.12	44.8 (+0.4)	2.29 (+0.17)

^{1}H and ^{13}C NMR Data Comparison for Loline Dihydrochloride

	Ref 1		Ref 2, 3		Ref 5		37•2HCl	
No.	δ_C (75 MHz)	δ_H (300 MHz)	δ_C (75 MHz)	δ_H (300 MHz)	δ_C (100 MHz)	δ_H (400 MHz)	δ_C (100 MHz)	δ_H (400 MHz)
1	65.9	4.23	66.0 (+0.1)	4.23 (0.00)	66.0 (+0.1)	4.26 (+0.03)	66.1 (+0.2)	4.23 (0.00)
2	73.9	4.79	74.0 (+0.1)	4.79 (0.00)	74.0 (+0.1)	4.80 (+0.01)	74.1 (+0.1)	4.78 (−0.01)
3-A	64.2	3.55	64.2 (0.0)	3.57 (+0.02)	64.2 (0.0)	3.60 (+0.05)	64.3 (+0.1)	3.56 (+0.01)
3-B		4.15		4.12 (−0.03)		4.15 (0.00)		4.12 (−0.03)
5-A	58.1	3.73	58.2 (+0.1)	3.70 (−0.03)	58.2 (+0.1)	3.73 (0.00)	58.3 (+0.2)	3.72 (−0.01)
5-B		3.73		3.75 (−0.02)		3.79 (+0.06)		3.72 (−0.01)
6-A	31.6	2.28	31.6 (0.0)	2.27 (−0.01)	31.6 (0.0)	2.31 (+0.03)	31.7 (+0.1)	2.28 (0.00)
6-B		2.37		2.38 (+0.01)		2.42 (+0.05)		2.39 (+0.02)
7	83.2	4.72	83.4 (+0.2)	-	83.4 (+0.2)	4.75 (+0.03)	83.4 (+0.2)	4.72 (0.00)
7a	72.2	4.79	72.2 (0.0)	4.79 (0.00)	72.2 (0.0)	4.82 (+0.03)	72.3 (+0.1)	4.78 (−0.01)
NMe	36.5	2.79	36.4 (−0.1)	2.79 (0.00)	36.4 (−0.1)	2.83 (+0.04)	36.5 (0.0)	2.79 (0.00)

^{1}H and ^{13}C NMR Data Comparison for Loline

	Ref 1		37	
No.	δ_C (75 MHz)	δ_H (300 MHz)	δ_C (125 MHz)	δ_H (500 MHz)
1	67.8	3.03	68.3 (+0.5)	3.34 (+0.31)
2	73.4	3.71	74.1 (+0.7)	4.03 (+0.32)
3-A	60.6	2.10	61.2 (+0.6)	2.42 (+0.32)
3-B		3.10		3.41 (+0.31)
5-A	54.0	2.62	54.7 (+0.7)	2.93 (+0.31)
5-B		2.77		3.08 (+0.31)
6-A	33.5	1.65	34.1 (+0.6)	1.97 (+0.32)
6-B		1.74		2.05 (+0.31)
7	81.0	4.10	81.8 (+0.8)	4.41 (+0.31)
7a	69.0	2.84	69.6 (+0.5)	3.15 (+0.31)
NMe	34.7	2.19	35.2 (+0.5)	2.49 (+0.30)

Notes

Data are reported in $CDCl_3$ (except for loline dihydrochloride, for which data are reported in D_2O). Good agreement between data sets was observed in all cases; note, however, that in most instances a systematic error between the data sets was observed, consistent with an error in the referencing of the spectra of the natural products. It was not possible to confirm the reference frequency for the NMR spectra of the natural materials. Reference frequencies employed in this study were: $CHCl_3$, δ_H 7.26; $CDCl_3$, δ_C 77.16. Midpoints of all multiplets have been reported. Values of $\Delta\delta_X$ are given in parentheses [$\Delta\delta_X = \delta_X$ (synthetic) − δ_X (natural)], for ease of comparison. *Signals associated with the diastereotopic C(6)H_2 protons were reported as overlapping in this case; the associated $\Delta\delta_H$ value therefore refers to the midpoint between the two distinct signals reported for the natural product.
